# Geometric and Electronic Engineering of Hydrogen Peroxide Production Electrocatalysts

**DOI:** 10.1007/s40820-026-02189-6

**Published:** 2026-05-13

**Authors:** Chang Zhang, Min Song, Huiyao Qi, Hongshang Hu, Lilong Zhang, Houfeng Zhang, Lipiao Bao, Huiying Yang, Jian Zhang, Xing Lu

**Affiliations:** 1 School of Chemistry and Chemical Engineering, Hainan University, Haikou, 570228 People’s Republic of China; 2 School of Materials Science and Engineering, Huazhong University of Science and Technology, Wuhan, 430074 People’s Republic of China; 3 School of Chemistry and Chemical Engineering, Huazhong University of Science and Technology, Wuhan, 430074 People’s Republic of China; 4 College of Design and Engineering, National University of Singapore, 21 Lower Kent Ridge Road, Singapore, 119077 Singapore

**Keywords:** Geometric and electronic structures, Electrocatalysts, Two-electron oxygen reduction reaction, Activity, Selectivity

## Abstract

Selective synthesis of hydrogen peroxide (H_2_O_2_) by two-electron oxygen reduction reaction (2e^–^ ORR) has emerged as an attractive alternative to the complex and energy-consuming anthraquinone method. Electrocatalysts play a crucial role in this process, as they determine catalytic activity and selectivity, which are strongly related to their geometric and electronic structures. However, the complexity of the composition and structure of the catalytically active site poses a significant challenge to understanding the relationship between the geometric/electronic structures and ORR activity/selectivity of the electrocatalyst. Herein, we systematically summarize and discuss the latest advances in the geometric and electronic modulations of electrocatalysts toward H_2_O_2_ synthesis. First, the basic principle of adsorption and reduction behaviors of oxygen species on the catalyst surface is introduced, and the influence of geometric and electronic structures is highlighted. Secondly, the recent advances in catalyst design via these two strategies are presented. Thirdly, the features of various electrocatalysts associated with their electrochemical performance are summarized and analyzed. Finally, the current challenges and future directions for the research and development of H_2_O_2_ production are discussed. This review provides insights that contribute to the development of next-generation catalysts for sustainable H_2_O_2_ production, addressing both environmental and energy challenges.

**Supplementary Information:**

The online version contains supplementary material available at 10.1007/s40820-026-02189-6.

## Highlights

- The effects of catalyst geometric/electronic structures on the adsorption configurations and strengths of oxygen reduction intermediates are discussed.
- The strategies for regulating the oxygen reduction reaction activity and selectivity of catalysts are summarized.
- The features of various electrocatalysts associated with their electrochemical performance are analyzed.

**Supplementary Information:**

The online version contains supplementary material available at 10.1007/s40820-026-02189-6.

## Graphical abstract

**Figure:**
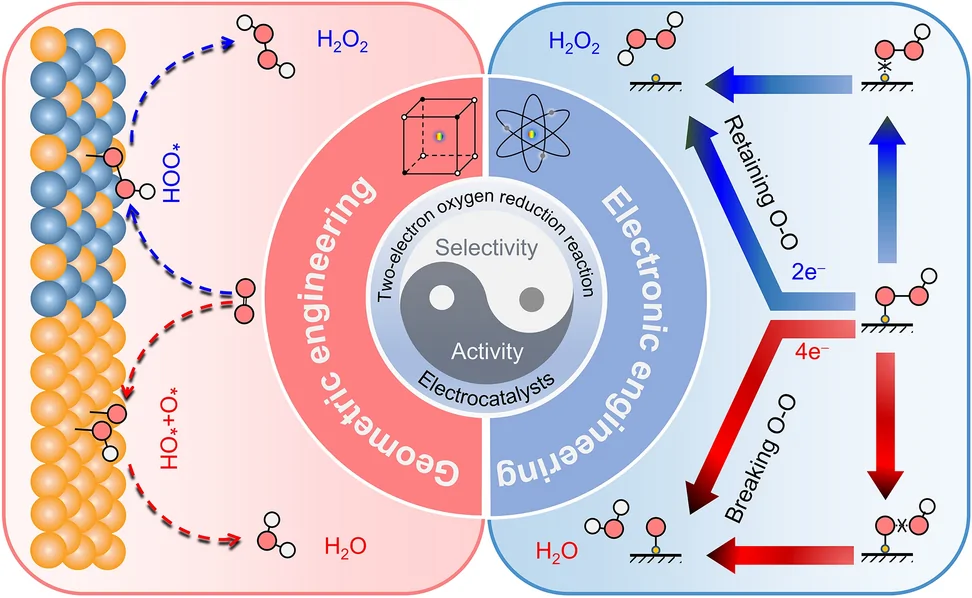

## Introduction

The hydrogen peroxide (H_2_O_2_) production via a two-electron oxygen reduction reaction (2e^–^ ORR) is one of the most investigated electrocatalytic processes [1–6]. This approach offers a green and sustainable alternative, which mitigates the reliance on hazardous substances and energy-intensive traditional anthraquinone industrial route [1–10]. The produced H_2_O_2_ can be applied for pollutant treatment, disinfection, and electrochemical oxidation of organic reactants [11–18]. Additionally, the 2e^–^ ORR can also be coupled with hydrogen or metal oxidation reactions for energy conversion and storage [19–23]. This multi-purpose nature endows 2e^–^ ORR with tremendous application prospects. Moreover, H_2_O_2_ has a vast global market size that reached US$3.61 billion in 2025 [24]. The increasing demand for semiconductors in consumer electronics, coupled with the introduction of new smartphones, creates a positive outlook for the H_2_O_2_ market [24]. This promising outlook provides a powerful driving force for the development of the electrosynthesis of electronic-grade H_2_O_2_. However, the efficiency of this process still suffers from the sluggish ORR kinetics and low 2e^–^ selectivity of catalysts [25–27]. As a result, extensive efforts have been devoted to exploring highly efficient electrocatalysts with high ORR activity and dominant 2e^–^ selectivity.

Early studies reveal that the highly 2e^–^ selective catalysts such as Au [28–30], Hg [31], and pristine carbons [32–34] have low ORR activity, whereas the highly active ORR catalysts such as Pt [35–41] and Pd [2, 42–45] possess low selectivity. To overcome these limitations, researchers have proposed a concept of combining highly selective and highly active elements in homogeneous catalysts to achieve both high selectivity and activity for 2e^–^ ORR. For example, the alloys composed of Pd–Au showed higher 2e^–^ selectivity compared to pure Pd metal and higher ORR activity compared to pure Au metal [46–50]. The reactivity and selectivity of catalysts were found to be dependent on the spacing between active elements, which means the electrocatalytic performance is highly associated with the geometric structure of the catalysts [46, 48]. Notably, the interaction of composition elements affects the electronic states of the catalyst, thus affecting its 2e^–^ ORR performance. For example, alloying Hg with other metals can affect the *OOH adsorption-free energy (ΔG_*OOH_) of the catalysts, which gives a volcanic relationship between ∆G_*OOH_ and the limiting potential of 2e^–^ ORR, with the Pd-Hg alloys located at nearly the top and outperforming other alloys [51]. This suggests that the electronic structure of the catalyst also plays a decisive role in its performance. Therefore, understanding the relationship between the geometric and electronic structure of catalysts and their ORR activity and 2e^–^ selectivity can provide theoretical guidance for the design of efficient catalysts. However, the complexity of the composition and structure of the catalytically active site presents a great challenge.

**Fig. 1:**
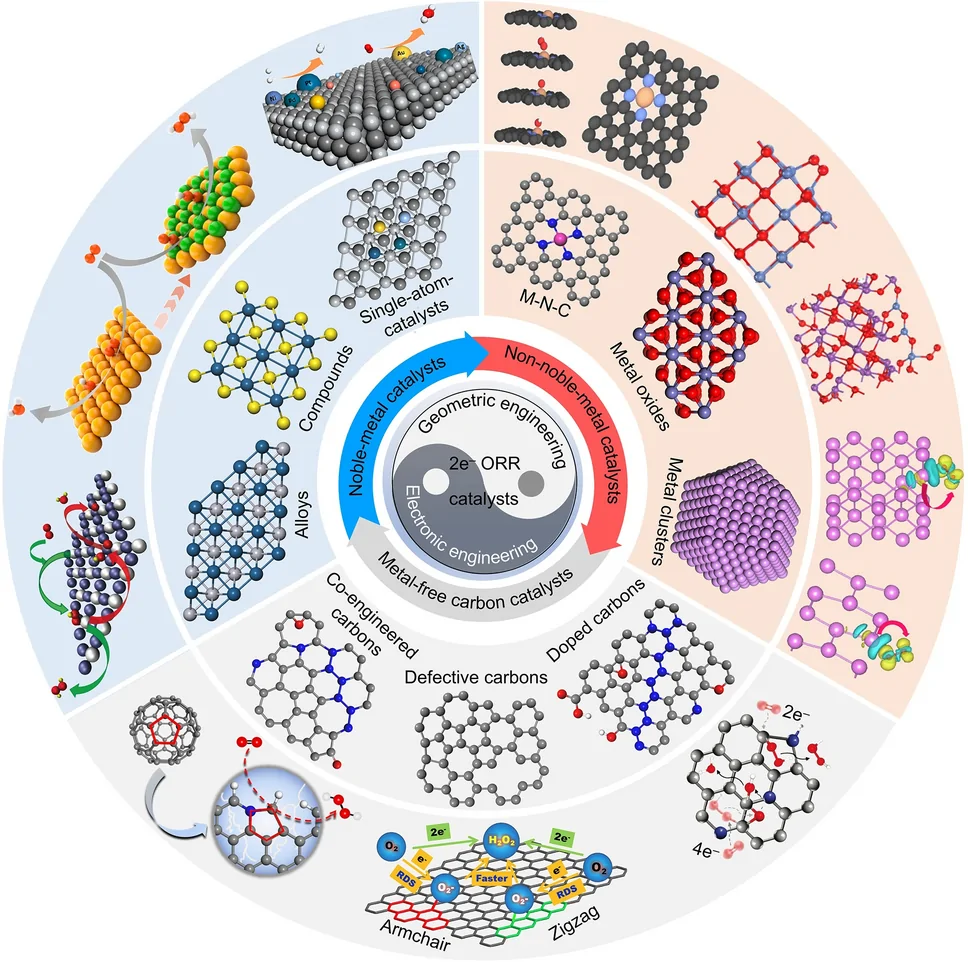
Classification and design principle of 2e^–^ ORR electrocatalysts

At present, with the development of characterization techniques and theoretical understanding, significant progress has been made in the experimental observation and theoretical understanding of highly efficient 2e^–^ ORR catalysts. These advancements have broken down traditional barriers and led to new insights. However, the relationships between the geometric and electronic structures of catalysts and their ORR activity and 2e^–^ selectivity are still controversial. Herein, we focus on the adsorption and reduction behaviors of oxygen species on catalyst surfaces, elucidate the correlation between catalytic performance (i.e., activity and selectivity) and catalyst structural characteristics (including geometric and electronic structures), and systematically categorize recent advances in this field into three major categories of noble-metal-based, non-noble-metal-based, and metal-free carbon catalysts, as shown in Fig. 1. Furthermore, we provide a comprehensive analysis of the properties of these catalysts, highlighting trends in ORR activity and 2e^–^ selectivity. This exploration aims to offer new insights and guide future research toward developing highly efficient 2e^–^ ORR catalysts for sustainable H_2_O_2_ production and beyond.

## Fundamentals

### ORR Pathways

Understanding the mechanistic pathways of ORR is essential for developing efficient catalysts. The ORR can proceed into different pathways, primarily categorized based on the number of electrons transferred and the intermediates formed [2, 43, 52]. The reaction mechanism and products are detailed as follows. All potentials are corrected to the reversible hydrogen electrode (RHE).

One-electron pathway:

Overall reaction:

1 $$\mathrm{O}_2 + \mathrm{H}^+ + \mathrm{e}^- \to \mathrm{OOH}\cdot \qquad \mathrm{E}^0 = -0.13\ \text{V (vs. RHE)}$$

Generally, the first step of ORR is considered to be a proton-coupled electron transfer (PCET) process under acidic conditions:

2 $${\text{* + O}}_{{2}} { } \to {\text{ *O}}_{{2}}$$

3 $${\mathrm{*O}}_{{2}} {\text{ + H}}^{ + } {\text{ + e}}^{ - } { } \to {\text{ *OOH}}$$

On the metal surface with high oxygen affinity, electrons may spontaneously transfer to O_2_ to form superoxygenated species [43].

4 $${\text{* + O}}_{{2}} {\text{ + e}}^{ - } { } \to {\text{ *O}}_{{2}}^{ - }$$

5 $${\mathrm{*O}}_{{\mathrm{2}}} ^{ - } {\text{ + H}}_{{\mathrm{2}}} {\mathrm{O}} \to {\text{*OOH + OH}}^{ - }$$

The intermediates of this pathway are highly active and usually unstable, but it is of great significance for the study of reaction mechanisms and intermediate states.

Two-electron pathway:

Overall reaction:

6 $$\mathrm{O}_2 + 2\mathrm{H}^+ + 2\mathrm{e}^- \to \mathrm{H}_2\mathrm{O}_2 \qquad \mathrm{E}^0 = 0.70\ \text{V (vs. RHE)}$$

In alkaline conditions with pH > 11.6, the product is HO_2_^–^.

Overall reaction:

7 $$\mathrm{O}_2 + \mathrm{H}_2\mathrm{O} + 2\mathrm{e}^- \to \mathrm{HO}_2^- + \mathrm{OH}^- \qquad \mathrm{E}^0 = 0.74\ \text{V (vs. RHE)}$$

Some studies [43, 53, 54] have shown that ORR undergoes a proton–electron uncoupling process under alkaline conditions:

8 $${\text{* + O}}_{{2}} {\text{ + e}}^{ - } { } \to {\text{ *O}}_{{2}}^{ - }$$

9 $${\mathrm{*O}}_{{2}}^{ - } {\text{ + H}}_{{2}} {\text{O }} \to {\text{ *OOH + OH}}^{ - }$$

10 $${\text{*OOH + e}}^{ - } { } \to {\text{ * + HO}}_{{2}}^{ - }$$

The 2e^–^ ORR products may undergo self-decomposition.

In acidic conditions:

11 $${\mathrm{2H}}_{{2}} {\mathrm{O}}_{{2}} { } \to {\text{ 2H}}_{{2}} {\text{O + O}}_{{2}}$$

12 $${\mathrm{H}}_{{\mathrm{2}}} {\mathrm{O}}_{{\mathrm{2}}} \to {\mathrm{2OH}\cdot}$$

In alkali conditions:

13 $${\mathrm{2HO}}_{{2}}^{ - } { } \to {\text{ 2OH}}^{ - } {\text{ + O}}_{{2}}$$

14 $${\mathrm{HO}}_{{\mathrm{2}}} ^{ - } {\text{ + H}}_{{\mathrm{2}}} {\mathrm{O}} \to {\mathrm{OH}}^{ - } {\text{ + 2OH}\cdot}$$

Three-electron pathway:

Overall reaction:

15 $$\mathrm{O}_2 + 3\mathrm{H}^+ + 3\mathrm{e}^- \to \mathrm{H}_2\mathrm{O} + \mathrm{OH}\cdot \qquad \mathrm{E}^0 = 0.73\ \text{V (vs. RHE)}$$

The generated hydroxyl radical (OH‧) is usually regarded as a strong oxidizing agent, which can degrade organic pollutants and destroy complex molecular structures. Thus, the three-electron pathway plays a crucial role in specific environmental catalytic and degradation processes [52].

Four-electron pathway:

Overall reaction:

16 $$\mathrm{O}_2 + 4\mathrm{H}^+ + 4\mathrm{e}^- \to 2\mathrm{H}_2\mathrm{O} \qquad \mathrm{E}^0 = 1.23\ \text{V (vs. RHE)}$$

According to the adsorption configuration of O_2_ on the catalyst surface, four-electron (4e^–^) ORR can be divided into two pathways under acidic conditions:

Associative pathway:

17 $${\text{* + O}}_{{2}} { } \to {\text{ *O}}_{{2}}$$

18 $${\mathrm{*O}}_{{2}} {\text{ + H}}^{ + } {\text{ + e}}^{ - } { } \to {\text{ *OOH}}$$

19 $${\text{*OOH + H}}^{ + } {\text{ + e}}^{ - } { } \to {\text{ *O + H}}_{{2}} {\mathrm{O}}$$

20 $${\text{*O + H}}^{ + } {\text{ + e}}^{ - } { } \to {\text{ *OH}}$$

21 $${\text{*OH + H}}^{ + } {\text{ + e}}^{ - } { } \to {\text{ * + H}}_{{2}} {\mathrm{O}}$$

Dissociative pathway:

22 $${\mathrm{O}}_{{2}} { + 2* } \to {\text{ 2*O}}$$

23 $${\text{2*O + 2H}}^{ + } {\text{ + 2e}}^{ - } { } \to {\text{ 2*OH}}$$

24 $${\text{2*OH + 2H}}^{ + } {\text{ + 2e}}^{ - } { } \to {\text{ 2* + 2H}}_{{2}} {\mathrm{O}}$$

In alkaline conditions, the product becomes OH^–^.

Overall reaction:

25 $$\mathrm{O}_2 + 2\mathrm{H}_2\mathrm{O} + 4\mathrm{e}^- \to 4\mathrm{OH}^- \qquad \mathrm{E}^0 = 1.23\ \text{V (vs. RHE)}$$

Except for the direct 4e^–^ pathway, the 2e^–^ ORR products may be reactants for further reduction, which are considered as an indirect “2e^–^ + 2e^–^” pathway.

In acidic conditions:

26 $${\text{* + O}}_{{2}} {\text{ + 2H}}^{ + } {\text{ + 2e}}^{ - } { } \to {\text{ * + H}}_{{2}} {\mathrm{O}}_{{2}}$$

27 $${\text{* + H}}_{{2}} {\mathrm{O}}_{{2}} {\text{ + 2H}}^{ + } {\text{ + 2e}}^{ - } { } \to {\text{ 2H}}_{{2}} {\mathrm{O}}$$

In alkaline conditions:

28 $${\text{* + O}}_{{2}} {\text{ + 2e}}^{ - } {\text{ + H}}_{{2}} {\text{O }} \to {\text{ * + HO}}_{{2}}^{ - } {\text{ + OH}}^{ - }$$

29 $${\text{* + HO}}_{{2}}^{ - } {\text{ + 2e}}^{ - } {\text{ + H}}_{{2}} {\text{O }} \to {\text{ * + 3OH}}^{ - }$$

On the one hand, the specific pathway and mechanism of ORR are related to the properties of the surface and interface [55–59]. In acidic conditions, the inner-Helmholtz plane (IHP) is constructed by solvent water dipoles, chemisorbed oxygen molecules (*O_2_), and specifically adsorbed hydroxyl ions (*OH^–^) from water dissociation, the outer-Helmholtz plane (OHP) is occupied by solvated oxygen molecules, electrolyte anions, and hydronium ions (H_3_O^+^) [56]. The inner-sphere mechanism with a proton-coupled electron transfer process is favored in environments where the catalyst surface can strongly adsorb O_2_ and intermediates [56]. In alkaline conditions, the adsorbed *OH^–^ in IHP comes from the electrolyte, and the H_3_O^+^ in OHP is replaced by alkali metal ion (e.g., Na^+^), in which the outer-sphere mechanism with proton–electron uncoupling process becomes more favorable due to the lower overpotential and the stabilization of intermediates by *OH^–^, leading to a higher yield of peroxide [56]. Especially, when the upper turning potential (UTP) of cyclic voltammetry is below the potential of zero charge (PZC), negative charge accumulates on the electrode surface, attracting alkali metal ions to the interface and repelling H^+^ ions [57]. As a result, the crucial intermediate (*OOH) is stabilized, lowering the energy barrier of the reactions, which increases the kinetics and the 2e^–^ selectivity of ORR [57]. However, the UTP for typical ORR is positive compared to the PZC. Besides, the H_2_O_2_ is more stable and applicable in acidic conditions than in alkaline conditions. In this context, the pH and interface of electrolytes conducive to the 2e^–^ ORR are not suitable for practical applications.

On the other hand, the pathway of ORR is strongly dependent on the geometric and electronic structures of the catalyst. For the targeted production of H_2_O_2_, the key lies in preserving the O–O bond against cleavage during the reduction process. First, the adsorption configuration of O_2_ on the catalyst surface plays a decisive role. The dual sites favor the dissociative pathway toward H_2_O formation, whereas isolated single sites facilitate the associative pathway with the formation of *OOH, which is the critical intermediate for H_2_O_2_ generation. Therefore, rationally regulating the geometric structure of the catalyst to ensure the isolation of active sites is highly favorable for suppressing the competing reaction. Second, excessively strong adsorption on active sites tends to induce *O–OH bond breaking and subsequent 4e^–^ ORR to H_2_O. By tuning the electronic structure of the catalyst, such as the d-band center, the adsorption strength toward key intermediates can be regulated, promoting *OOH desorption and protonation to form H_2_O_2_. Furthermore, it is essential to prevent further reduction of the generated H_2_O_2_. Given that O_2_ is paramagnetic while the product H_2_O_2_ is diamagnetic, modulating the spin state of the catalyst enables selective adsorption of O_2_ and repulsion of H_2_O_2_, thereby effectively inhibiting the hydrogen peroxide reduction reaction.

### Geometric Effects

Geometric effects involve the strategic manipulation of the physical dimensions and surface characteristics of catalysts, such as cluster size, which play a vital role in regulating catalytic performance. In the context of the 2e^–^ ORR, the core focus lies on the intrinsic differences between single-atom and cluster active sites in the O_2_ adsorption configurations. Thus, our discussion in this work focuses on the spacing of active sites in modulating the O_2_ adsorption behavior and its correlation with ORR selectivity.

**Fig. 2:**
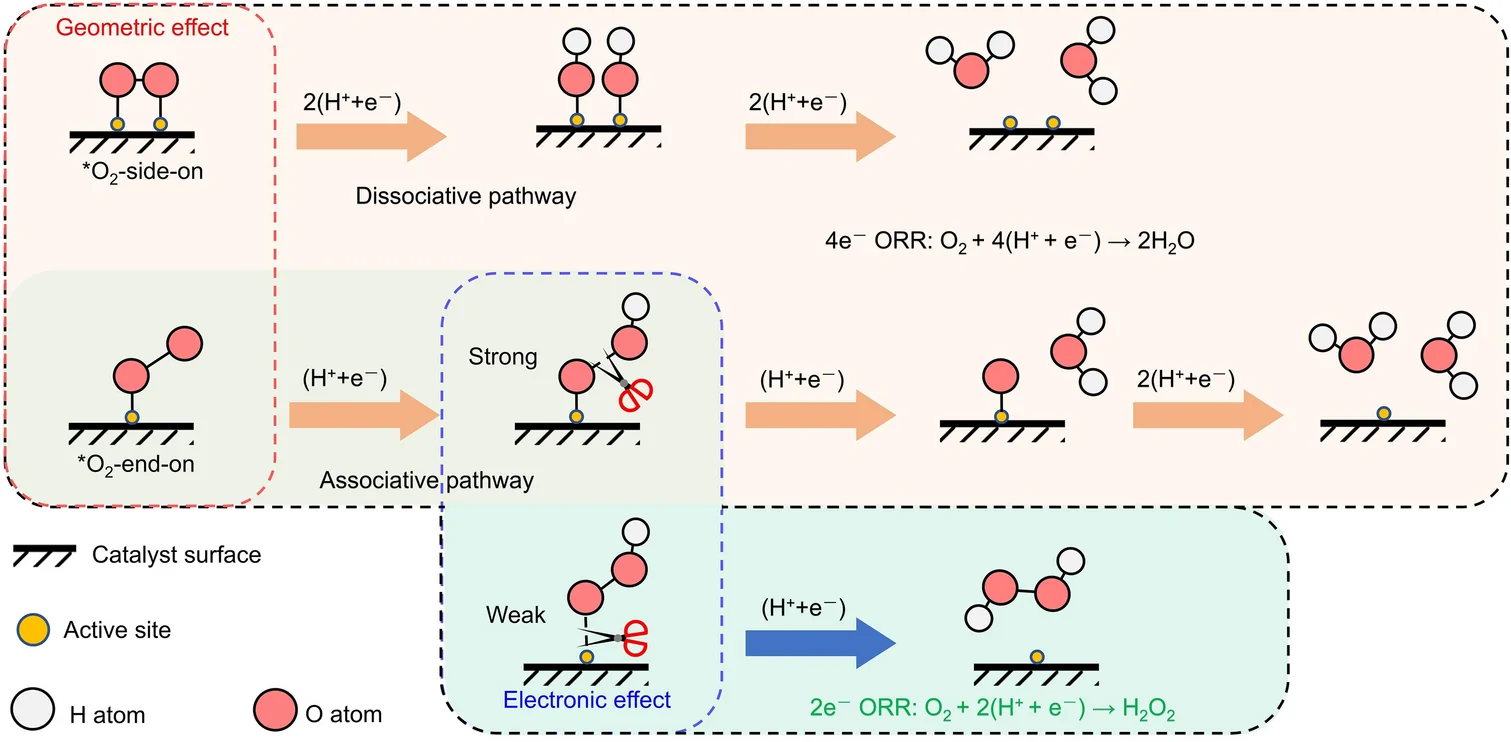
Geometric and electronic effects on oxygen adsorption and reduction behaviors at different catalyst surfaces

When active sites are close to each other, as illustrated in Fig. 2, O_2_ molecules typically adsorb in a “side-on” (Yeager-mode) configuration. This adsorption mode elevates the tendency of O–O bond cleavage to form *O intermediates, thus ultimately facilitating H_2_O production [10, 60, 61]. In contrast, isolated active sites favor the “end-on” (Pauling-mode) adsorption of O_2_, which enables the electrochemical protonation of O_2_ into the *OOH intermediate. The stability of the *OOH intermediate is closely correlated with its adsorption strength on the catalyst surface, with weaker adsorption promoting its reduction to H_2_O_2_, while stronger adsorption may lead to further dissociation to H_2_O [10, 13, 60–63]. Notably, the adsorption strength of such reaction intermediates is highly related to the electronic structure of the catalysts (see below).

**Fig. 3:**
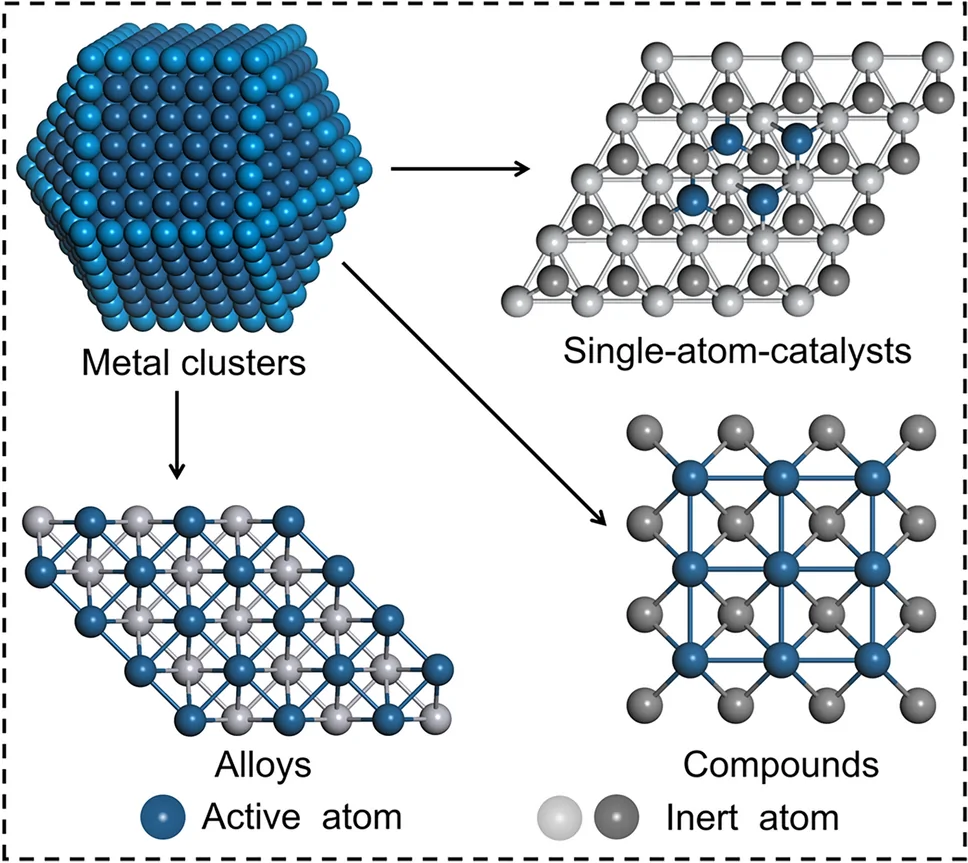
Geometric engineering of electrocatalysts for isolating active sites

As discussed above, isolated active sites are favorable for H_2_O_2_ electrosynthesis, as they stabilize the associative reaction pathway. Common strategies to construct isolated active sites include the fabrication of alloy catalysts, compounds, and single-atom catalysts, as shown in Fig. 3. The preparation method of a material has a significant impact on its geometric structure. For example, adjusting the proportion of active metals in precursors avoids the formation of contiguous active ensembles. Schiffrin et al. [46] synthesized Au_1-x_Pd_x_ nanoalloys with Pd content ≤ 8%, achieving atomic dispersion of Pd, while excess Pd (x > 15%) led to cluster formation. Besides, controlling the reaction conditions inhibits migration and aggregation. Geyer et al. [64] synthesized PtP_2_ nanocrystals via a colloidal hot-injection method, where the phosphorus precursor was rapidly injected into the platinum precursor, avoiding Pt aggregation and widening Pt atomic spacing. In contrast, thermal reduction of Pt precursors without P precursor injection yielded metallic Pt nanocrystals. Moreover, designing supports with strong anchoring capabilities stabilizes active atoms. Choi et al. [65] used high sulfur content zeolite-templated carbon (17 wt% S) to anchor Pt, forming isolated Pt sites. In contrast, the low sulfur content support and non-sulfur support lack sufficient sulfur-containing anchoring sites to strongly interact with Pt atoms, leading to Pt aggregation.

### Electronic Effects

Electronic effects refer to the strategic modification of the electronic properties of catalysts, such as the d-band center, charge distribution, and spin states of the catalysts, to optimize their adsorption strength in specific reactions [41, 54, 66–73].

#### Catalysts with d Electrons

**Fig. 4:**
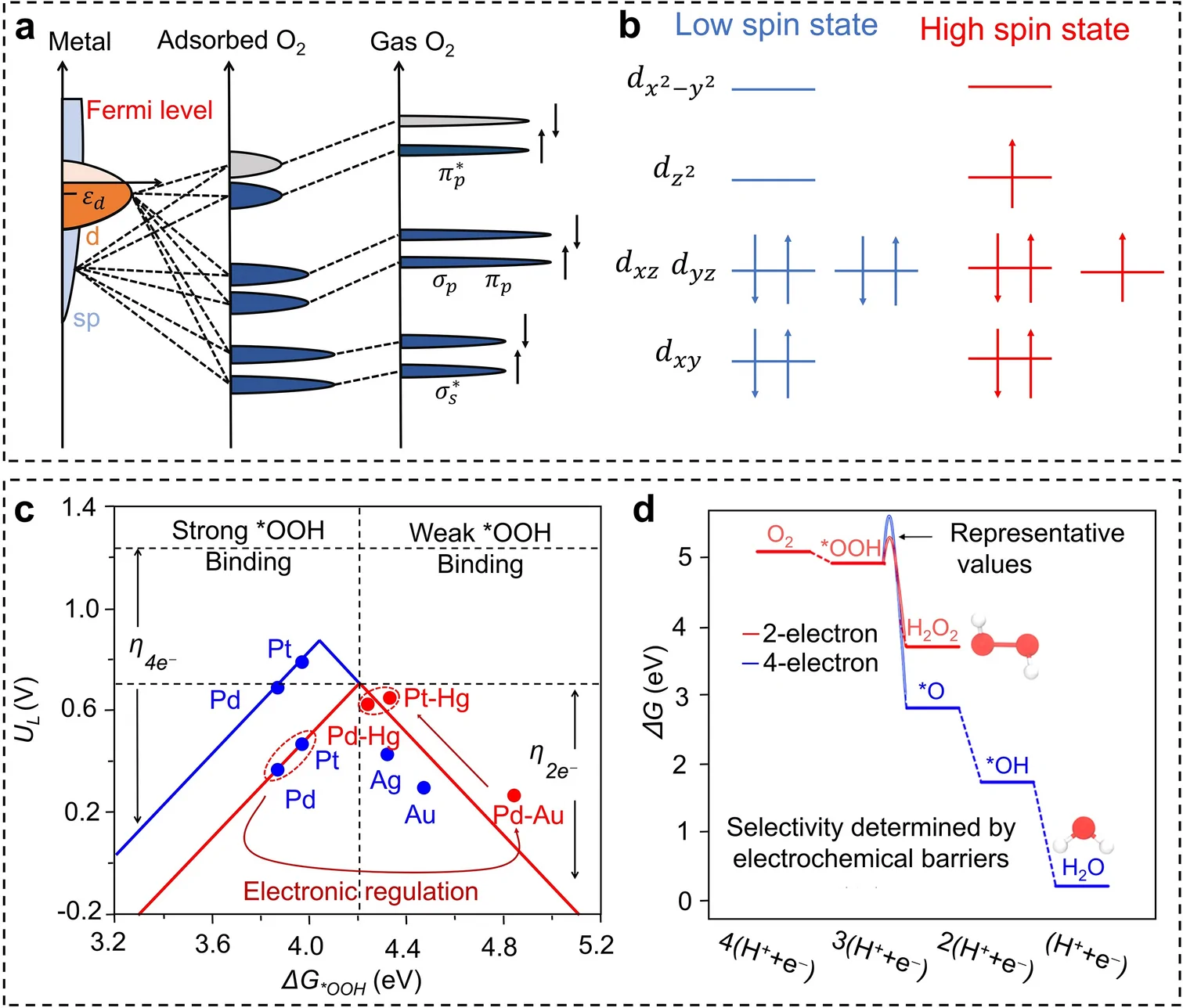
Electronic engineering. **a** Molecular orbital model for O_2_ adsorption on a transition metal surface that exhibits the typical sp- and d-bands of a transition metal (leftmost) that cause shifts and broadening of the O_2_ molecular orbitals (rightmost) arising from interaction with the surface (adsorbed O_2_, middle) [43]. Copyright 2018, American Chemical Society. **b** 3d electron configuration of low-spin-state and high-spin-state CoN_4_ with adsorption of *OOH [85]. Copyright 2022, American Chemical Society. **c** Theoretical volcano plot for the 2e^–^ (red) and 4e^–^ (blue) ORR, with the limiting potential (*U*_L_) as a function of *OOH adsorption-free energy (∆G_*OOH_) [51, 86]. Copyright 2013, Springer Nature and 2014 American Chemical Society. **d** Free-energy diagrams for the 2e^–^ (red) and 4e^–^ (blue) ORR on Au(111), with the electrochemical barriers from *OOH to H_2_O_2_ or *O are for illustration purposes [87]. Copyright 2018, American Chemical Society

For the transition metals with d electrons, the adsorption strength to O_2_ rests with the filling of the antibonding states owing to their approach to the Fermi level [41, 74–76]. As shown in Fig. 4a, the electronic states of transition metal surfaces are classified into *sp*-bands and *d*-bands. Given that transition metals possess similar *sp*-bands, the difference in adsorption behavior across transition metal surfaces is mainly governed by the coupling between the adsorbate valence states and the metal *d*-states. To simplify the description, the widely adopted d-band center theory was introduced, where the d-band center (ε_d_) is defined as the average energy of the *d*-band (Fig. 4a). The upshift/downshift of the ε_d_ results in the decreased/increased filling of antibonding, which causes stronger/weaker adsorption [54, 66–68, 77–80]. Thus, adjusting the ε_d_ of the catalysts can regulate the ORR pathway. Take the active site for example, the electron acceptor inert sites can attract electrons from the active site and cause the downshift of the ε_d_, avoiding the break of O–O bond and being conducive to H_2_O_2_ formation. According to the crystal-field theory [81, 82], as shown in Fig. 4b, the bonding of the transition metal to the coordination atom is accompanied by energy level splitting, the original d-orbitals of the metal splitting into e_g_ high-energy orbitals (${d}_{{z}^{2}}$ as well as ${d}_{{x}^{2}-{y}^{2}}$) and t_2g_ low-energy orbitals (${d}_{xy}$,${d}_{xz,}$ and ${d}_{yz}$), in which the ${d}_{{x}^{2}-{y}^{2}}$ and ${d}_{xy}$ orbitals are asymmetric with the bonding orbitals of O_2_ [83]. As a result, the metal with a high-spin state has more orbitals occupied by an unpaired electron with a single spin, which promotes the transfer of local spin current and causes strong adsorption of O_2_ [80]. In addition, a high-spin state was found to stabilize the *OOH intermediate owing to its higher capacitance than the low-spin state when they have the same PZC [84].

The relationship between adsorption strength and ORR selectivity can be described by a volcano plot displayed with limiting potential (*U*_*L*_, defined as the lowest potential where all the reaction steps are downhill in free energy) as a function of intermediate adsorption-free energy (e.g., ∆G_*OOH_), as shown in Fig. 4c. The vertical axis *U*_*L*_ acts as a descriptor for ORR activity, with a more positive value corresponding to a higher catalytic activity. The horizontal axis ∆G_*OOH_ free energy characterizes the adsorption strength of the *OOH intermediate on active sites, with a larger value representing a weaker spontaneous adsorption tendency. Notably, two horizontal dash lines parallel to the x-axis correspond to *U*_*L*_ = 0.7 V and *U*_*L*_ = 1.23 V, which denote the standard potentials of the 2e⁻ ORR (E^0^ = 0.7 V vs. RHE) and 4e⁻ ORR (E^0^ = 1.23 V vs. RHE), respectively. The potential differences between the catalyst and these two standard potential lines are defined as the overpotentials (*η*) for the 2e⁻ ORR and 4e⁻ ORR. Besides, a vertical dash line corresponding to ∆G_*OOH_ = 4.22 eV, which is parallel to the y-axis, bisects the 2e⁻ ORR volcano plot (red line) into two regions. The right side of the volcano corresponds to weak adsorption between catalysts and oxygen species, where the O–O bond is relatively stronger and favors the 2e^–^ pathway, but the kinetics is sluggish and the overpotential is huge. The left side of the volcano exhibits strong adsorption, resulting in weaker O–O bonds that are prone to form H_2_O. However, over-strong adsorption hinders desorption, also leading to huge overpotential. The apex of the 2e⁻ ORR volcano represents the ideal catalyst, which features* U*_*L*_ = 0.7 V and ∆G_*OOH_ = 4.22 eV. In contrast, the apex of the 4e⁻ ORR volcano plot (blue) cannot reach 1.23 V, which stems from the unfavorable scaling relationship between the adsorption energies of reaction intermediates [87]. Notably, the right-hand sides of the volcano plots for the 2e⁻ and 4e⁻ ORR are identical. This is because the first step of ORR *O_2_ + H⁺ + e⁻ → *OOH*,* which follows the relationship ΔG_*OOH_ = 4.92 – *ΔG*_*1*_, where *ΔG*_*1*_ denotes the Gibbs free energy of the first elementary step. Importantly, the selectivity of the catalysts is also determined by the kinetics, as shown in Fig. 4d. Although the dissociation of *OOH to *O is more thermodynamically favorable than the formation of H_2_O_2_, the electrochemical barriers are higher. This accounts for why the thermodynamic trend is inconsistent with the experimentally observed phenomenon and highlights the key role of kinetic parameters in determining the selectivity of the reaction.

#### Catalysts with s or p Electrons

**Fig. 5:**
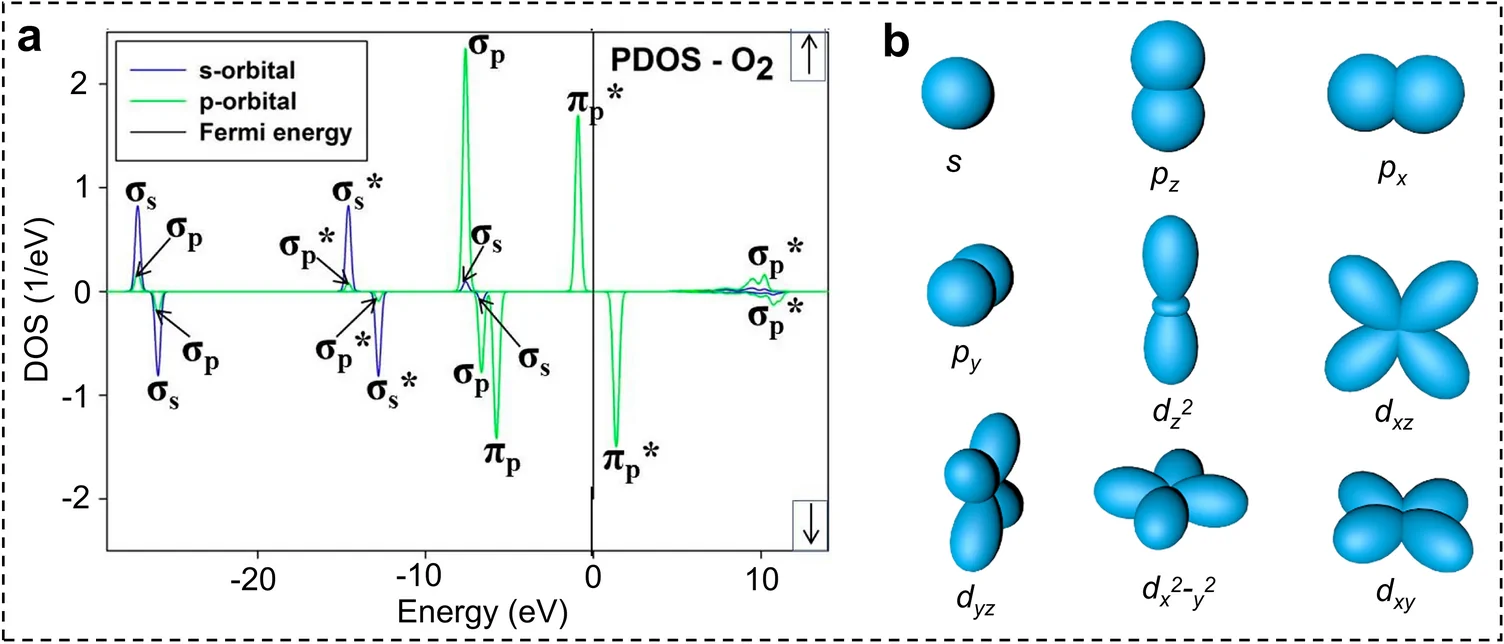
**a** DOS and molecular orbital diagram for gas-phase O_2_ [92]. Copyright 2015, MDPI. **b** Different atomic orbital (*s, p, d*) diagrams

For the main-group elements with the outermost s or p electrons (such as Al, Sn, Ge, Ga, and Pb), the interaction strength between the catalyst surface and O_2_ is typically too strong, which is unfavorable for the electrosynthesis of H_2_O_2_ [88–92]. As shown in Fig. 5a, gas-phase O_2_ features two singly occupied π* orbitals, leading to a triplet spin state and magnetic character [92]. When O_2_ adsorbs on a metal surface, its valence orbitals shift to new energies and become occupied if positioned below the Fermi level of the combined system. This energy shift is primarily governed by the interaction between O_2_ and the *s/p* electrons of the metal. The electronic engineering methods and principles of *s* and *p*-block metals are broadly similar to those of *d*-block metals that involve introducing electron donors or acceptors to adjust the charge distribution or spin density, thereby modulating the adsorption strength of O_2_. However, the *s/p-*orbitals lack the same degree of spatial overlap and electronic delocalization found in *d*-orbitals, resulting in a limited ability to create ligand field splitting [91]. As shown in Fig. 5b, the structural diversity of *s-* and *p*-orbitals is far inferior to that of *d*-orbitals. We will discuss the mechanisms in detail by which electronic engineering regulates the ORR activity and 2e^–^ selectivity of the main-group elements in the subsequent cases.

## Advanced Electrocatalysts via Geometric and Electronic Engineering

As discussed above, the 2e^–^ ORR performance of electrocatalysts is predominantly governed by their geometric and electronic structures. Geometric engineering enables precise regulation of adsorption configurations, while electronic engineering modulates the adsorption strength of key intermediates.

Building on these fundamental principles, this section systematically summarizes the latest advances in advanced 2e^–^ ORR electrocatalysts, categorized into noble-metal-based, non-noble-metal-based, and metal-free carbon materials. Each category integrates tailored geometric and electronic engineering strategies to achieve synergistic enhancement of catalytic activity and 2e^–^ selectivity. Below, we elaborate on the design strategies, structural characteristics, and electrochemical performances of each catalyst type, highlighting the direct correlation between fundamental structure–performance relationships and practical catalyst development.

### Noble-Metal-Based Catalysts

Noble-metal catalysts (e.g., Pt- and Pd-based materials) have long been regarded as the benchmark for high-performance ORR electrocatalysts due to their exceptional catalytic activity and stability [2, 35–45]. However, pure noble metals either adsorb O_2_ overly strongly or overly weakly, which is not suitable for 2e^–^ ORR [3, 43, 86]. Fortunately, by adjusting the geometric and electronic structure, the adsorption mode and strength of the intermediates on the catalysts can be optimized.

#### Alloys

**Fig. 6:**
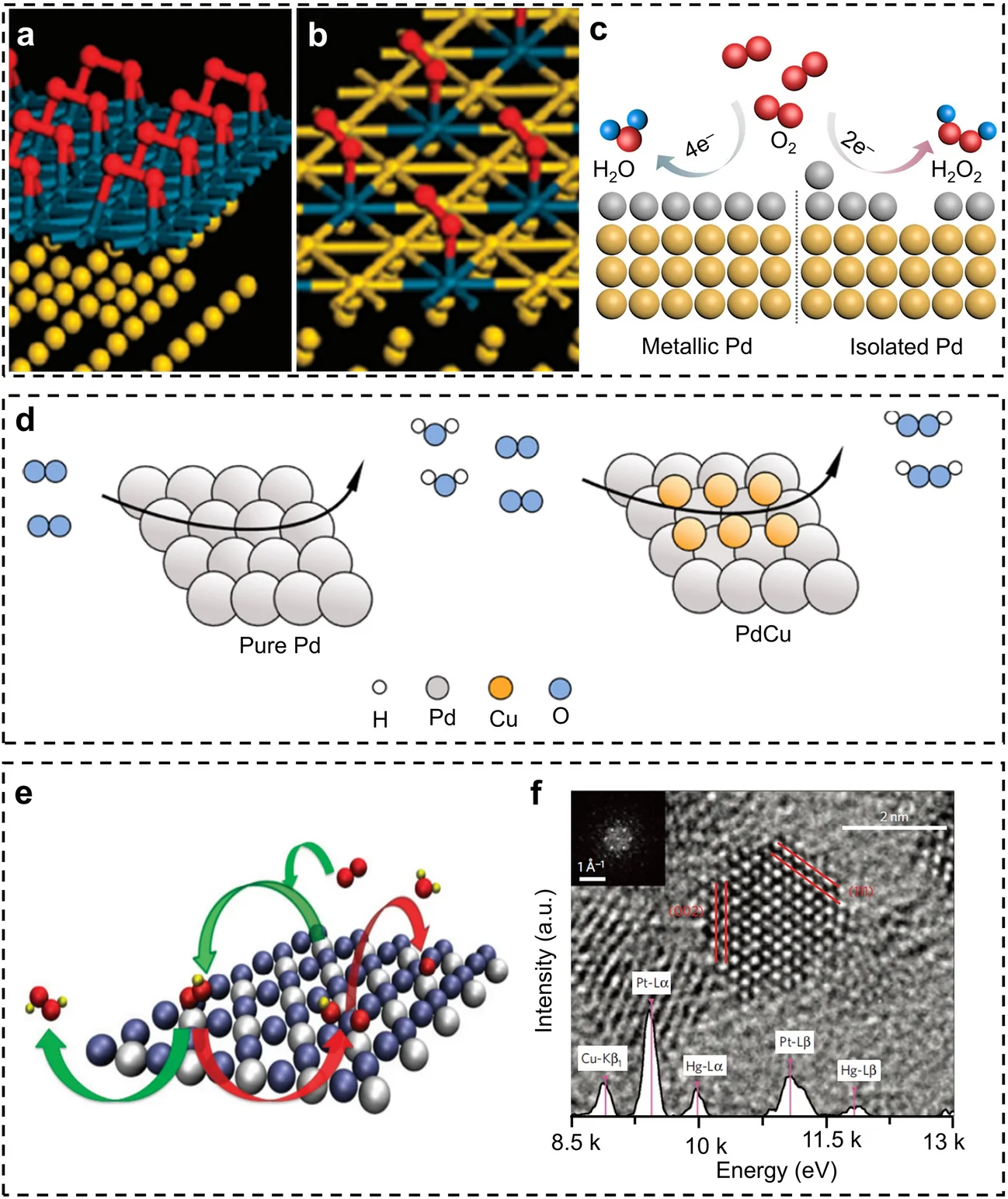
Engineering of noble-metal alloys for 2e^–^ ORR. Adsorption of O_2_ (red) on the surfaces of **a** Pd monolayer (blue) or **b** Pd single atom incorporated in an Au (111) matrix [46]. Copyright 2011, American Chemical Society. **c** Schematic diagrams of the ORR pathway on the surface of metallic or isolated Pd on the Au surface, with the gray, yellow, blue, and red balls representing the Pd, Au, H, and O atoms, respectively [93]. Copyright 2024, American Chemical Society. **d** Illustration of ORR on the pure Pd or PdCu surface [97]. Copyright 2023, American Chemical Society. **e** Representation of ORR on the PtHg_4_ (110) surface [86]. Copyright 2013, Springer Nature. **f** HRTEM image of the individual Pt-Hg nanoalloy, with the corresponding EDS spectrum of the particle superimposed on top [86]. Copyright 2013, Springer Nature

Alloying is an effective strategy for the deliberate arrangement and combination of different metals, which form metallic solid solutions without a fixed atomic ratio while retaining the host lattice structure. Schiffrin and coworkers conducted a density functional theory (DFT) simulation where they placed “guest” transition metal atoms into an Au host matrix to create dilute alloys [46]. The simulation results reveal that O_2_ tends to undergo “side-on” adsorption on the pure Pd surface, whereas it exhibits an inclination for “end-on” adsorption on isolated Pd sites surrounded by Au atoms, as shown in Fig. 6a, b. Consequently, they prepared Au_1-x_Pd_x_ nanoalloys with variable Pd content supported on Vulcan XC-72. Electrochemical test results disclose that the 2e^–^ selectivity approaches nearly 95% when the Pd content is comparatively low (*x* = 0.08), where the Pd atoms are spaced apart from each other. Further increase of Pd content results in a decline in the 2e^–^ selectivity, falling below 10% for *x* = 0.5, where the Pd atoms are in proximity. This article has demonstrated both theoretically and experimentally that the geometric structure of the catalyst exerts a regulatory effect on its ORR performance. Moreover, Wang et al. further explored the systematic compositional design of Au@Pd alloy to optimize the balance between activity and selectivity [93]. As shown in Fig. 6c, they found that metallic Pd boosts 4e^–^ ORR, while isolated Pd sites favor 2e^–^ ORR. DFT calculations suggest enhanced H_2_O_2_ selectivity and ORR activity at Pdn (*n* ≤ 3), and larger Pd clusters are active for 4e^–^ ORR. X-ray absorption fine structure (XAFS) is a powerful atomic-scale characterization technique that reveals the local coordination environment, valence state, and bond distances of target elements in materials [94–96]. They thus applied XAFS to investigate the influence of Pd species in a series of Au@Pd alloys on 2e^–^ ORR performance. The results revealed that a decrease in Pd content in Pd/Au alloys induces a significant negative shift of the peak corresponding to the 1*s* → 5*p* transition in X-ray absorption near-edge structure (XANES), which reflects the expansion of Pd–Pd atomic spacing. Extended X-ray absorption fine structure (EXAFS) characterization identified the Pd_4_ cluster in Au@Pd(15:1) as the optimal structure for the 2e^–^ ORR. Its Pd–Pd coordination number of 2.6 ensures moderate electronic interaction, thus avoiding excessively weak *OOH adsorption, and inhibits O–O bond cleavage via the isolation effect of the Au substrate. Experimental results show Au@Pd NWs with Pd_4_ as the primary structure possess optimal H_2_O_2_ performance in acidic electrolytes, with a high mass activity (7.05 A mg^–1^ at 0.4 V) and selectivity (nearly 95%) in 0.1 M HClO_4_.

Apart from introducing active sites on the surface of inert metals, introducing inert sites on the surface of active metals can also regulate the ORR performance. As shown in Fig. 6d, Kang and coworkers reported that the coverage of Cu on the Pd surface can stabilize the Pd–O bond, resulting in enhanced 2e^–^ selectivity of the Pd site [97]. Notably, simply isolating the active sites with inert sites does not always produce highly efficient catalysts. The optimal active sites for 2e^–^ ORR should have moderate adsorption strength to oxygen, which requires the coordination of different components to regulate the electronic states. As previously mentioned, there is a volcano relationship between the adsorption strength of the catalyst for oxygen species and its ORR performance, and the ∆G_*OOH_ can be employed as a descriptor for the 2e^–^ and 4e^–^ trends. Rossmeisl et al. utilized ∆G_*OOH_ as descriptors to screen the alloys for 2e^–^ ORR based on the concept of the active site isolated by the inert sites [86]. Hg was selected as the inert surrounding component due to its inherent catalytic inactivity and broad stability potential window in acidic electrolytes. Moreover, Hg can be easily electrodeposited on Pt(111) [98], forming an ordered PtHg_4_ lattice structure, where each Pt atom is surrounded by 4 Hg atoms to create isolated active sites, as shown in Fig. 6e. This geometric arrangement favors the “end-on” adsorption of O_2_, thereby facilitating the selective 2e^–^ ORR. In contrast, disordered alloy structures (e.g., Ag₃Pt) readily induce Pt aggregation, which in turn triggers the “side-on” adsorption of O_2_ and thus promotes O–O bond cleavage, favoring the 4e^–^ ORR pathway. Accordingly, they prepared Pt-Hg alloy by electrodeposition of Hg from HgClO_4_ on a modified Pt disk electrode. Electrochemical measurements revealed that Pt-Hg alloy possesses an onset potential where the ring and the disk coincide at approximately 0.6 V, a 2e^–^ selectivity of 96% in the region between 0.2 and 0.4 V, and a hydrogen peroxide current density of 3 mA cm^–2^ in 0.1 M HClO_4_ at room temperature. This represents the state-of-the-art of 2e^–^ ORR catalysts over the past ten years. Furthermore, the authors developed a Pt-Hg/C catalyst through the same electrodeposition method on the commercial Pt/C dropped onto the glassy carbon disk electrode. The high-resolution transmission electron microscopy (HRTEM) image and energy-dispersive X-ray spectroscopy (EDS) spectrum in Fig. 6f show the structure of isolated Pt surrounded by Hg, which is the key factor in achieving excellent 2e^–^ ORR performance. Regarding specific activity (normalized to the surface area of Pt), nanoparticulate Pt-Hg/C demonstrates 4–5 times the activity of polycrystalline Pt-Hg alloy. In the second year following the publication of this article, they further developed a Pd-Hg alloy catalyst based on the same design concept, which also exhibited excellent 2e^–^ ORR performance [51].

#### Compounds

**Fig. 7:**
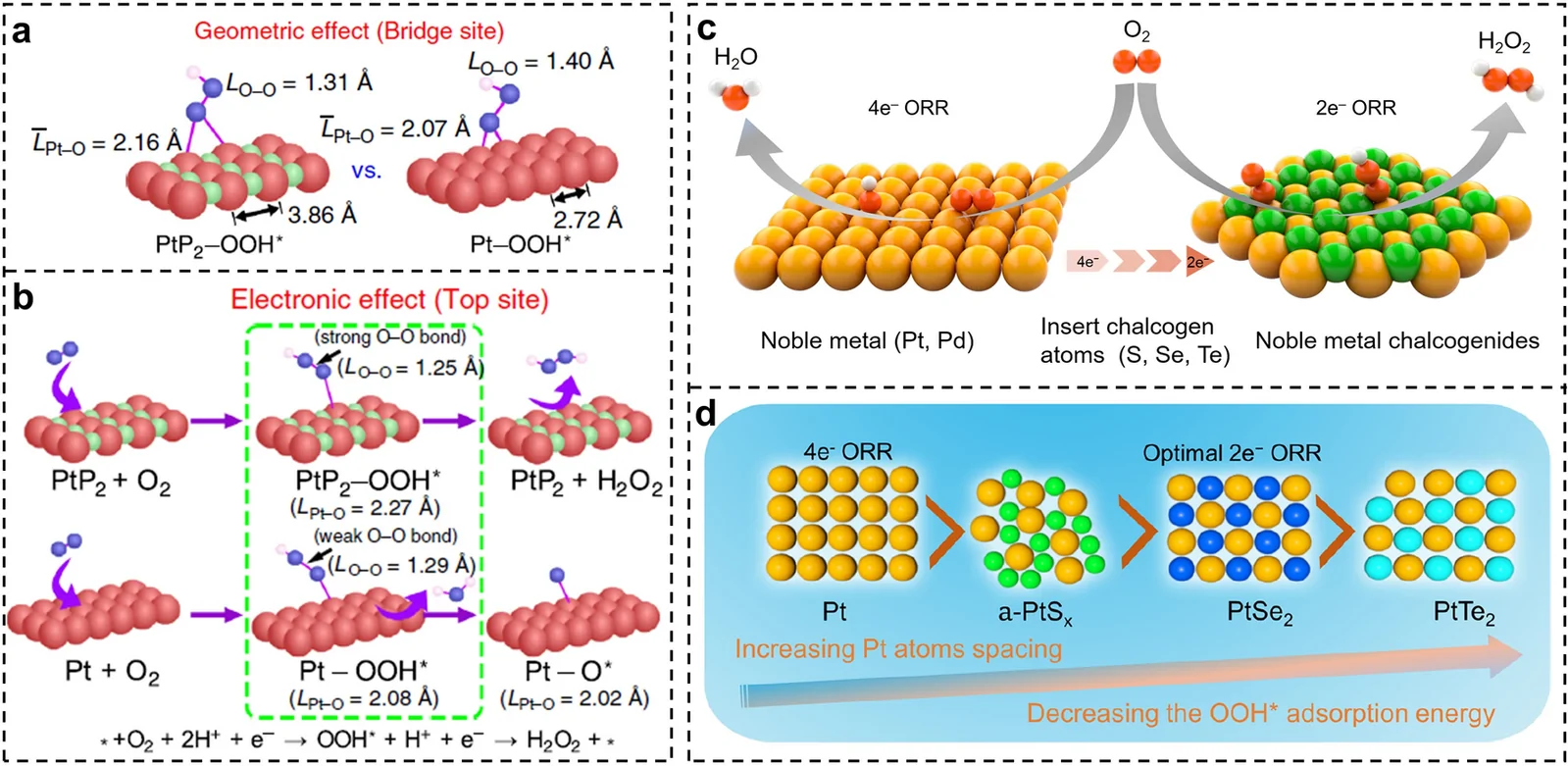
Engineering of noble-metal compounds for 2e^–^ ORR. Schematic diagrams of **a** *OOH adsorption behaviors on the bridge site and **b** ORR intermediates for PtP_2_ and Pt during 2e^–^ ORR and 4e^–^ ORR pathways [64]. Copyright 2020, Springer Nature. Schematic diagrams of **c** ORR pathways on the surface of pure Pt/Pd and Pt/Pd chalcogenides and **d** catalytic properties of Pt chalcogenides [100]. Copyright 2023, American Chemical Society

Taking the stability into consideration, many metals have more negative dissolution potential than oxygen reduction potential and will dissolve during the operating condition, leaving a Pd or Pt shell, resulting in a decreased selectivity for 2e^–^ ORR [62, 99]. In contrast, the majority of nonmetals have excellent resistance to dissolution. In this matter, metal compounds may be potential catalysts for stable H_2_O_2_ electroproduction. They are chemical combinations of a metal with another element in a specific atomic ratio and possess a completely distinct lattice structure, which endows them with enhanced structural and electrochemical stability. Geyer et al. reported an ultrasmall and monodisperse colloidal PtP_2_ nanocompound that achieves 2e^–^ ORR at near zero-overpotential (0.7 V) and exhibits near 100% 2e^–^ selectivity at 0.27 V in 0.1 M HClO_4_ [64]. Experimental results demonstrated that the as-reported PtP_2_ catalyst exhibits exceptional stability during a 120-h chronoamperometric test at a constant potential of 0.4 V, which enables an accumulated neutral H_2_O_2_ concentration of 3.0 wt% in 600 mL. The in situ XAFS characterization revealed that the oxidation state of Pt decreases from the initial + 3.26 to + 2.72 at the early stage of ORR (0.7 V), indicating electron transfer from Pt to reaction intermediates. Subsequently, the oxidation state stabilizes at + 2.25 throughout the reaction process (0.5 ~ 0.3 V). This finding confirms that the presence of P can stabilize Pt at a specific valence state, thereby ensuring the sustainability of H_2_O_2_ production. Furthermore, they employed in situ attenuated total reflection infrared spectroscopy (ATR-IR) to directly identify key intermediates, including adsorbed O_2_, *OOH, and *HOOH, thereby validating the 2e^–^ ORR pathway. Through DFT calculation, they discovered that the insertion of the P can adjust the geometric structure of the catalyst, widen the atomic spacing of Pt, which is conducive to the “end-on” configuration, and thereby weaken the Pt–*OOH bond (Fig. 7a). At the same time, P can regulate the electronic structure of the active Pt center, causing fewer electrons to fill the antibonding orbital of the O–O bond and suppressing the dissociation of *O–OH (Fig. 7b), thereby achieving outstanding 2e^–^ ORR performance. By regulating the elements, the geometric and electronic structures of the catalyst can be adjusted flexibly and conveniently, thereby achieving the regulation of performance. Wang and coworkers constructed a series of Pt/Pd chalcogenide catalysts with isolated active sites [100]. As shown in Fig. 7c, the introduction of chalcogen atoms can turn the O_2_ adsorption configuration from “side-on” to “end-on,” thereby turning the ORR process from 4e^–^ to 2e^–^. DFT calculations indicate that the increase in the radius of interstitial atoms resulted in the increased active center spacing and decreased *OOH adsorption energy (Fig. 7d). This allows for the optimization of the catalysts 2e^–^ ORR performance. Experiment results supported the theoretical predictions that the PtSe_2_ catalysts with moderate adsorption strength have the best ORR activity and 2e^–^ selectivity. In addition, PtSe_2_ showed excellent electrochemical stability with negligible attenuation after 10 k cycles of accelerated durability test (ADT).

#### Single-Atom Catalysts

**Fig. 8:**
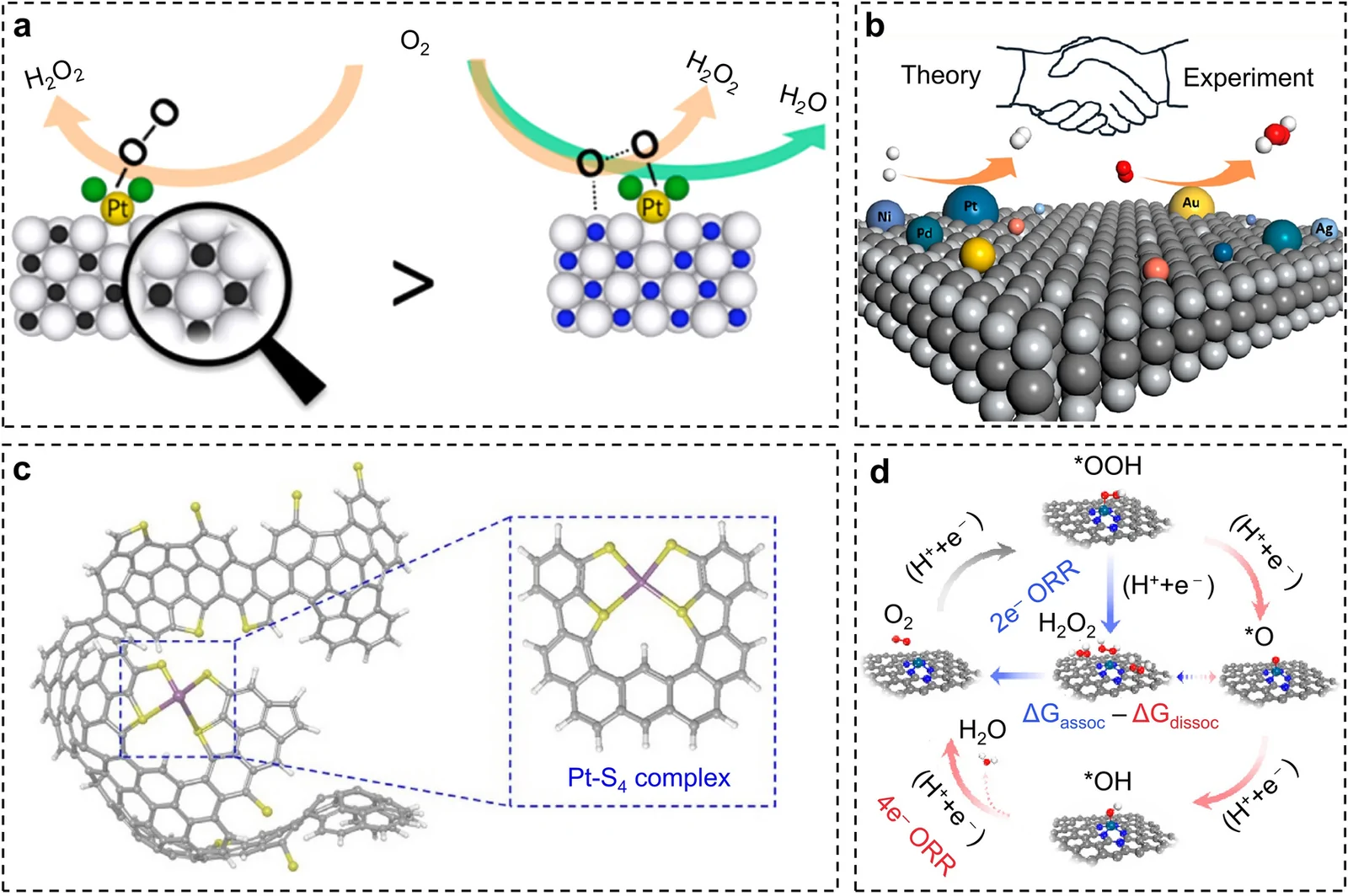
Engineering of noble-metal single-atom catalysts for 2e^–^ ORR. **a** Schematic diagram of the ORR process on the Pt1/TiC and Pt1/TiN [103]. Copyright 2017, American Chemical Society. **b** Schematic diagram of hydrogen evolution reaction and 2e^–^ ORR on the M (M = Ag, Au, Pd, Pt, etc.) located on the modified TiC surface [104]. Copyright 2019, American Chemical Society. **c** Proposed atomic structure of the Pt/HSC [65]. Copyright 2016, Springer Nature. **d** Schematic of the 2e^–^ or 4e^–^ ORR pathway on the Pt − N − C motif with nearly graphitic-N dopants [107]. Copyright 2024, American Chemical Society

Single-atom catalysts (SACs) have intrinsically isolated active sites and are appropriate for serving as 2e^–^ ORR catalysts [101]. Lee et al. found that decreasing the cluster size of Ptn on TiN can improve the 2e^–^ selectivity of the catalyst [102]. The Pt sites (0.2 wt%) atomically dispersed on TiN support show the highest selectivity of approximately 60% for H_2_O_2_ generation. Furthermore, the authors investigated the effect of support on the performance of catalysts. Pt1/TiC shows higher activity, selectivity, and stability for 2e^–^ ORR than Pt1/TiN [103]. DFT calculations revealed that the oxygen species possess a strong affinity to Pt1/TiN, possibly serving as surface poisoning species. In contrast, Pt1/TiC can preserve the O–O bond, thus facilitating 2e^–^ ORR (Fig. 8a). Apart from the support, the type of active metal also affects the performance of the catalysts. Woo and coworkers studied the stability and electrocatalytic activity of a series of metals (M = Cu, Ag, Au, Ni, Pd, and Pt) single atoms located on a Ti/C support (Fig. 8b) [104]. Interestingly, theoretical prediction revealed that most metal (except Pd) atoms tend to aggregate into clusters instead of remaining isolated on the pristine TiC surface, yet their dispersion can be stabilized by carbon vacancy defects. This observation is ascribed to the enhanced surface–metal bonding strength, which results from the suppressed spin magnetic moment of the adsorbed single atoms on the defective TiC surface rather than the pristine counterpart. They further applied ∆G_*OOH_ as descriptors to evaluate the 2e^–^ ORR performance of the catalysts. Au/TiC was predicted as the best catalyst for 2e^–^ ORR. The above theoretical predictions were confirmed by experimental results.

Within the framework of single-atom catalysts, the coordinating atoms serve as reliable anchors for the active elements by their robust interactions with metals, thereby effectively warding off the aggregation of these active components [101, 105, 106]. Choi et al. presented a sulfur-doped zeolite-templated carbon featuring a substantial sulfur content (17 wt%) and highly curved three-dimensional networks of graphene nanoribbons, which could stabilize a high loading of Pt (5 wt%) in the form of highly dispersed isolated sites [65]. The authors demonstrated that the XANES absorption edge of Pt shifts to higher energy with increasing S content in the support, suggesting a higher oxidation state of Pt. They further validated via EXAFS that Pt/ZTC (without S doping) exhibits a strong Pt–Pt coordination peak at 2.75 Å with a coordination number (CN) of 9.2, confirming the presence of Pt in the form of nanoclusters. Pt/LSC (low S content, 4 wt%) shows both Pt–Pt (CN = 3.3) and Pt–S (CN = 2.4) coordination peaks indicating a mixed structure of atomically dispersed Pt and Pt clusters. In contrast, Pt/HSC (high S content, 17 wt%) only presents a Pt–S coordination peak at 2.29 Å (CN = 3.8) with no discernible Pt–Pt coordination signal directly, verifying that Pt atoms are fully isolated by S atoms and stably exist in a single-atom form. Experimental results revealed that the prepared Pt/HSC exhibits an onset potential of 0.71 V with the 2e^–^ selectivity of 90% in 0.1 M HClO_4_, which is far higher than Pt/LSC (55%) and Pt/ZTC (25%). This phenomenon is ascribed to the anchoring effect of S on Pt, which enables the formation of isolated active sites and consequently leads to a selective 2e^–^ ORR pathway rather than the conventional 4e^–^ ORR on the Pt clusters. However, the theoretical analysis revealed that the Pt-S_4_ configuration is thermodynamically more favorable toward the 4e^–^ rather than the 2e^–^ pathway. Further kinetic studies discovered that the second PCET of 4e^–^ ORR is accompanied by a significant reorganization process for breaking the O–O bond, resulting in large electrochemical barriers. Hence, Pt-S_4_ (Fig. 8c) is kinetically more inclined toward the 2e^–^ pathway. Thermodynamics determines the inherent feasibility of ORR pathways, while kinetics regulates the actual reaction rate. As a result, for the Pt–S_4_ site, the low overpotentials favor the thermodynamically preferred 4e^–^ ORR pathway, while high overpotentials prioritize kinetically facile 2e^–^ ORR pathways. This accounts for the phenomenon that the H_2_O_2_ selectivity of the catalyst decreases at potentials close to 0.7 V versus RHE.

In addition to their anchoring function, the coordinating atoms play a crucial role in fine-tuning the electronic structure of the active sites, thereby regulating the ORR performance. Jiang and coworkers reported that the pyrrolic-N coordination of Pt − N_4_ − C motifs, although featuring isolated Pt active sites, favors the O_2_ to H_2_O conversion. Once graphitic-N doping is introduced near Pt − N_4_ − C, the Pt active sites exhibit a remarkable shift in selectivity toward O_2_ to H_2_O_2_ conversion, as shown in Fig. 8d [107]. This is attributed to the enhanced electron donation from Pt to the adjacent graphitic-N dopant as compared to the Pt − N_4_ − C motifs. Undoubtedly, distinct coordination structures can bring diverse properties, and some are inherently favorable for 2e^–^ ORR. Peng et al. reported an ultralow content Pt (0.21 wt%) single atom anchored on the g-C_3_N_4_ nanosheet catalyst (Pt_0.21_/CN) that showed dominant H_2_O_2_ selectivity of 98% in 0.1 M KOH [108]. Combining experiments with DFT calculations, the authors revealed that the Pt single atom is coordinated with 3 N atoms to form a Pt-N_3_ configuration, and the charge near the C-N migrates to the surface of Pt, regulating its *OOH adsorption strength, thus promoting 2e^–^ ORR.

The SACs with coordination structures can further be optimized by increasing the density of active atoms. Li and coworkers synthesized a high-concentration (24.8 at%) single-atomic Pt site located on hollow CuSx catalysts through an ion exchange method [109]. The authors declared that the isolated Pt atom prevents O–O bond cleavage to form *O due to the absence of the *O-favored threefold hollow sites composed of several adjacent Pt atoms. Thus, ORR is limited to generating H_2_O_2_ through a 2e^–^ pathway. Moreover, they claimed that the hollow and porous structure can enhance mass transport and expose more reactive sites. Also, the strong coordination between Pt–S is resistant to oxygen corrosion. Consequently, the h-Pt_1_-CuS_x_ exhibited a dominant 2e^–^ selectivity of 92%-96% over a wide potential range of 0.05–0.7 V, a high H_2_O_2_ generation mass activity of 35 ± 4 A mg_cat_^–1^, and an excellent stability with less than 2% declines after 10 k ADT cycles.

In conclusion, eliminating ensemble active metal to prevent “side-on” adsorption constitutes the main concept for enhancing the 2e^–^ selectivity of the noble-metal catalysts. There are multiple approaches to accomplish this objective, such as alloying, forming compounds, and single-atom catalysts, through isolated active sites via inert sites. Besides, the surface poisoning or covering with inert species to prevent “side-on” adsorption through the steric hindrance effect can also improve the selectivity of 2e^–^ ORR [110–113]. However, the sole geometric regulation is incapable of controlling the adsorption strength between the active site and oxygen species, resulting in random performance of the catalysts. This can be addressed by regulating the electronic structure of the active sites through the interactions between surrounding atoms and active atoms. Therefore, geometric regulation and electronic regulation are complementary and interdependent, which contribute together to boosting the 2e^–^ ORR of the catalysts.

### Non-Noble-Metal-Based Catalysts

Non-noble-metal materials (such as Co–N–C [114, 115], CoS_2_ [116], Bi cluster [90], and Ni-C [117]) represent a promising alternative to noble-metal catalysts for the 2e^–^ ORR owing to their low cost and wide resources, although the catalytic activity and stability of traditional non-noble metals are inferior to those of noble metals (e.g., Pt and Pd) [43]. With the advancements in materials science and surface science, their performance is gradually improving through innovative regulation of the structure and electronic properties. Some non-noble-metal catalysts have demonstrated efficacy approaching or even surpassing that of noble metals. This section delves into the advancements in non-noble-metal catalysts enhanced by geometric and electronic engineering.

#### Transition-Metal Catalysts

Transition-metal catalysts stand at the forefront of research for catalysts due to their remarkable electronic versatility and structural flexibility. Specifically, transition metals typically possess partially filled d-orbitals, which provide a unique ability to facilitate various chemical transformations through versatile coordination geometries and variable oxidation states. Some metal compounds (e.g., NiPS_3_ [118], CoS_2_ [116], CoSe_2_ [119], Ni_3_B [120], etc. [121–123]), metal oxides (e.g., Fe_3_O_4_ [124, 125], Co_3_O_4_ [126], ZnO [127], and so on [128–134]), and metal hydroxide (e.g., Ni(OH)_2_ [135, 136]) have shown excellent performance for 2e^–^ ORR. For example, Zhao et al. revealed that the introduction of cationic vacancies on nickel phosphide (Ni_*2-x*_P-V_Ni_) can regulate the geometric and electronic properties of the catalyst for boosting 2e^–^ ORR performance [137]. As compared to the original nickel phosphide (Ni_3_P), the adsorption mode of the Ni_*2-x*_P-V_Ni_ changed O_2_ adsorption from “side-on” to “end-on,” and regulated the *OOH adsorption energy from over-strong to moderate, thus promoting ORR toward H_2_O_2_ production.

**Fig. 9:**
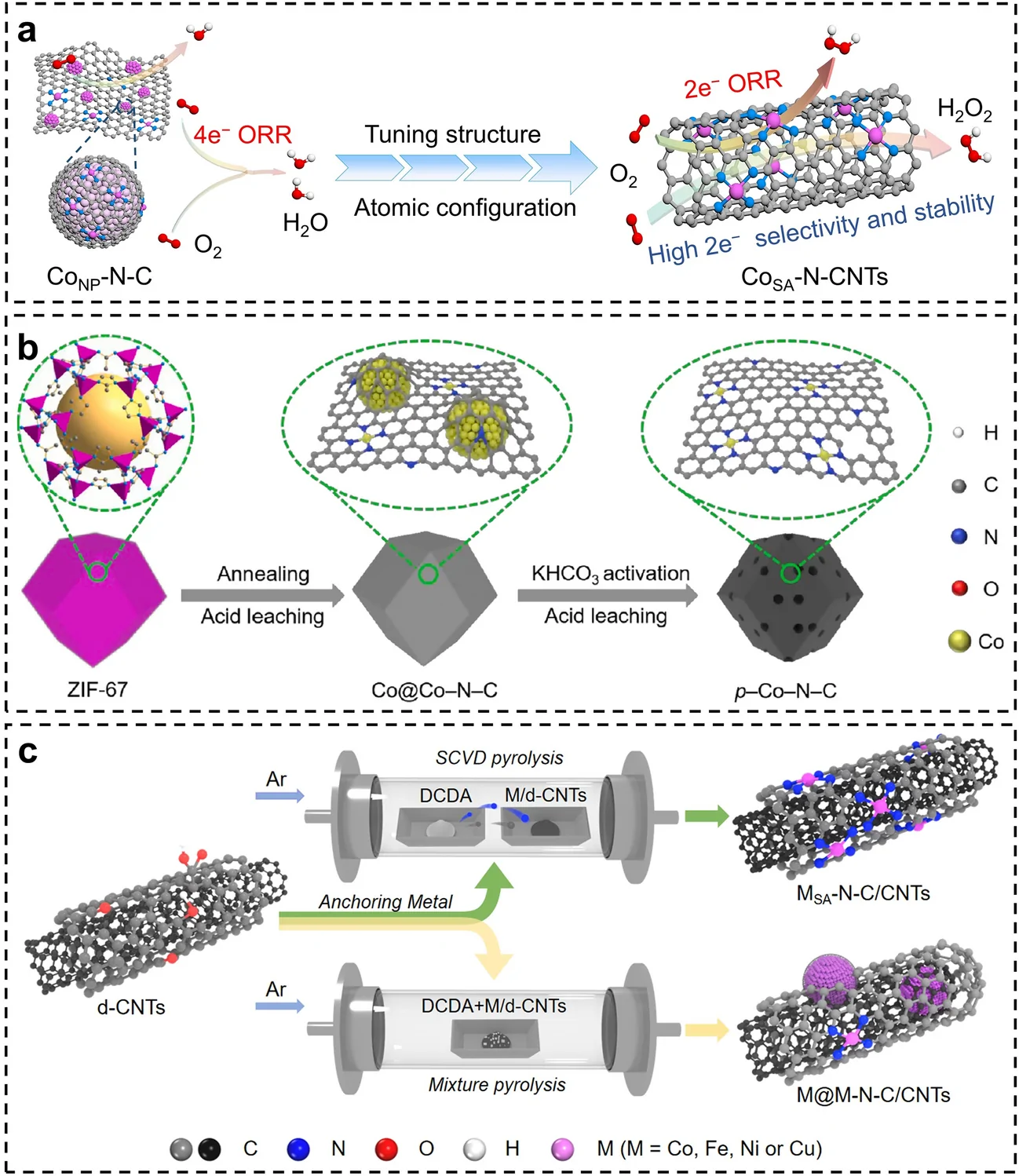
Geometric engineering cases of M–N-C catalysts. **a** Schematic illustration for the ORR pathways of Co_NP_-N–C and Co_SA_-N-CNTs [144]. Copyright 2022, Elsevier Inc. **b** Schematic diagram for the preparation of *p*-Co–N-C [14]. Copyright 2022, Elsevier Inc. **c** Schematic illustration for the SCVD strategy and the mixture pyrolysis strategy for the fabrication of Co_SA_-N–C/CNTs and Co@Co–N-C/CNTs [145]. Copyright 2024, Wiley–VCH

For the bulk transition metals, however, O_2_ adsorption on their surfaces predominantly leads to dissociation, which harms the formation of H_2_O_2_ [43]. In this context, transition metal–nitrogen–carbon (M–N–C) catalysts have attracted significant attention [138–143]. These catalysts possess a well-defined M-N_4_ coordination structure, providing isolated active sites for O_2_ adsorption. Nonetheless, the traditional pyrolysis preparation method tends to induce the agglomeration of metals, which poses a significant challenge to the H_2_O_2_ selectivity of the resultant catalysts. As shown in Fig. 9a, Zhang and Wang et al. reported that encapsulated Co nanoparticles located at N-doped carbon (Co_NP_-N–C) tend to a 4e^–^ ORR to form H_2_O, while the incorporation of atomically dispersed cobalt atoms embedded within nitrogen-doped carbon nanotubes (Co_SA_-N-CNTs) favors 2e^–^ ORR for H_2_O_2_ production [144]. They demonstrated that the Fourier-transform EXAFS (FT-EXAFS) spectrum of Co_SA_-N-CNTs exhibits a strong characteristic peak at 1.45 Å, which corresponds to the typical Co − N coordination bonds, confirming the atomic-scale dispersion of cobalt atoms within this catalyst. In contrast, Co_N__P_-N–C shows an intense characteristic peak at 2.14 Å, which is attributed to Co − Co metallic bonds, indicating the presence of a large number of cobalt nanoparticles formed through Co − Co coordination in the catalyst. The electrochemical results revealed that the Co_NP_-N–C catalyst exhibited a relatively low selectivity of merely 30%, whereas the Co_SA_-N-CNTs catalyst achieved an exceptional H_2_O_2_ selectivity exceeding 95%. Following this, they developed highly dispersed cobalt atoms anchored in porous N-doped carbon (*p*-Co–N–C) via a carbonization-alkalization-acidification strategy, as exhibited in Fig. 9b [14]. The *p*-Co–N–C achieved a high H_2_O_2_ selectivity of over 90% in acidic media, which is three times higher than that of Co nanoparticles encapsulated in N-doped carbon. Very recently, Zhang and Lu et al. designed a coaxial cobalt single-atom catalyst on carbon nanotubes (Co_SA_-N–C/CNTs) by an ingenious separation chemical vapor deposition (SCVD) strategy (Fig. 9c), which achieves higher ORR activity, dominant 2e^–^ selectivity, and superior stability in acid, compared to the counterpart Co nanoparticle catalyst prepared by traditional mixture pyrolysis [145].

**Fig. 10:**
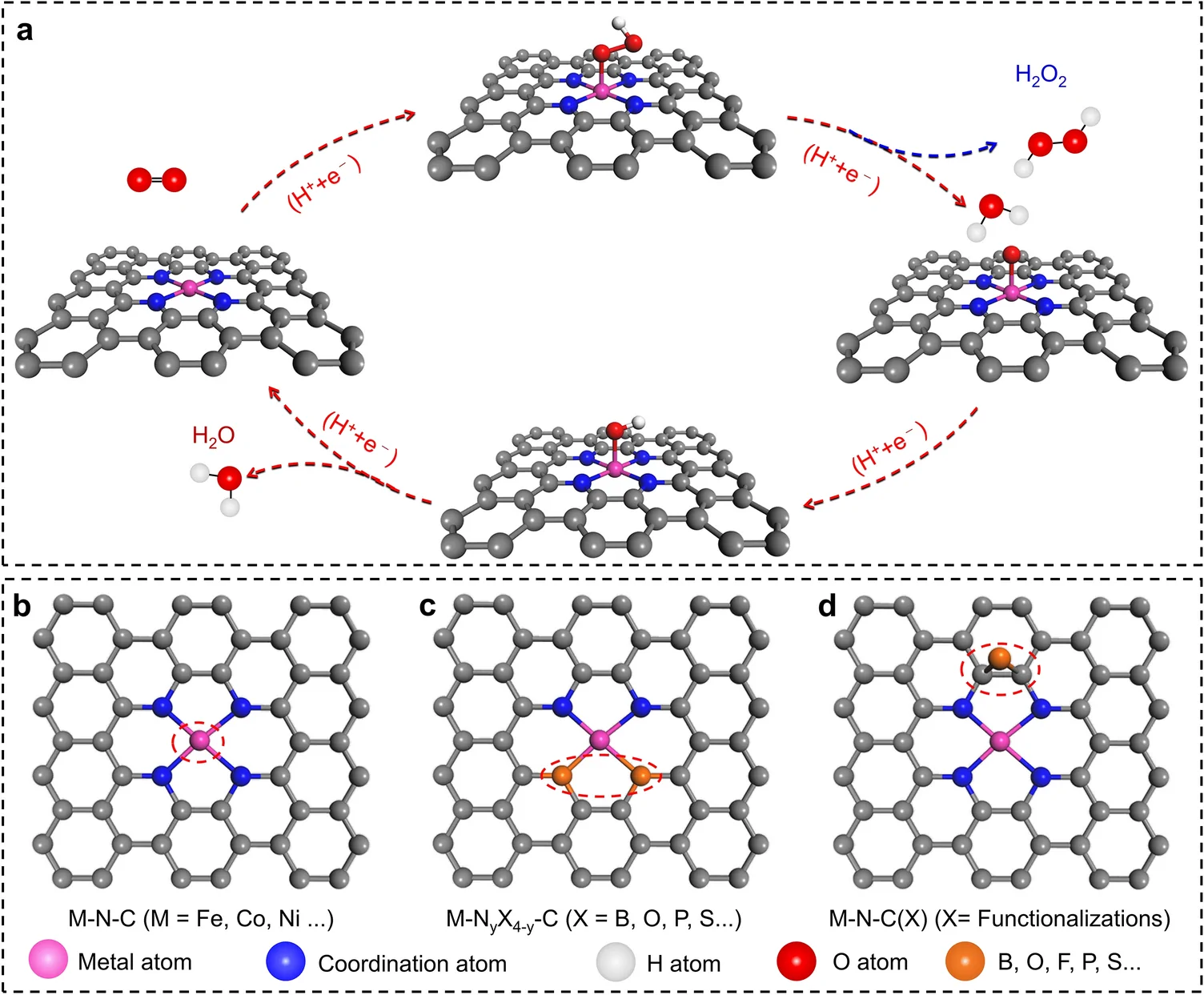
Electronic engineering cases of M–N–C catalysts. Schematic diagram of **a** ORR pathways, **b** central metal regulation, **c** coordination atom regulation, and **d** functionalization regulation of the M–N-C catalysts

Notably, due to the inherently isolated metal sites in M–N–C, O_2_ tends to adsorb in an "end-on" configuration, as shown in Fig. 10a, and its ORR pathway depends on the adsorption strength between the active sites and oxygen species. Therefore, electronic engineering for the regulation of adsorption strength is crucial to adjust the ORR pathway of M–N–C. The electronic regulation of M–N–C is achieved by modifying the central metal (Fig. 10b), coordinating atoms (Fig. 10c), and surrounding functionalizations (Fig. 10d), to fine-tune the adsorption strength of oxygen species at the active sites. Subsequently, we will present cases concerning the design of the electronic structure of M–N–C catalysts to boost their 2e^–^ ORR performance.

**Fig. 11:**
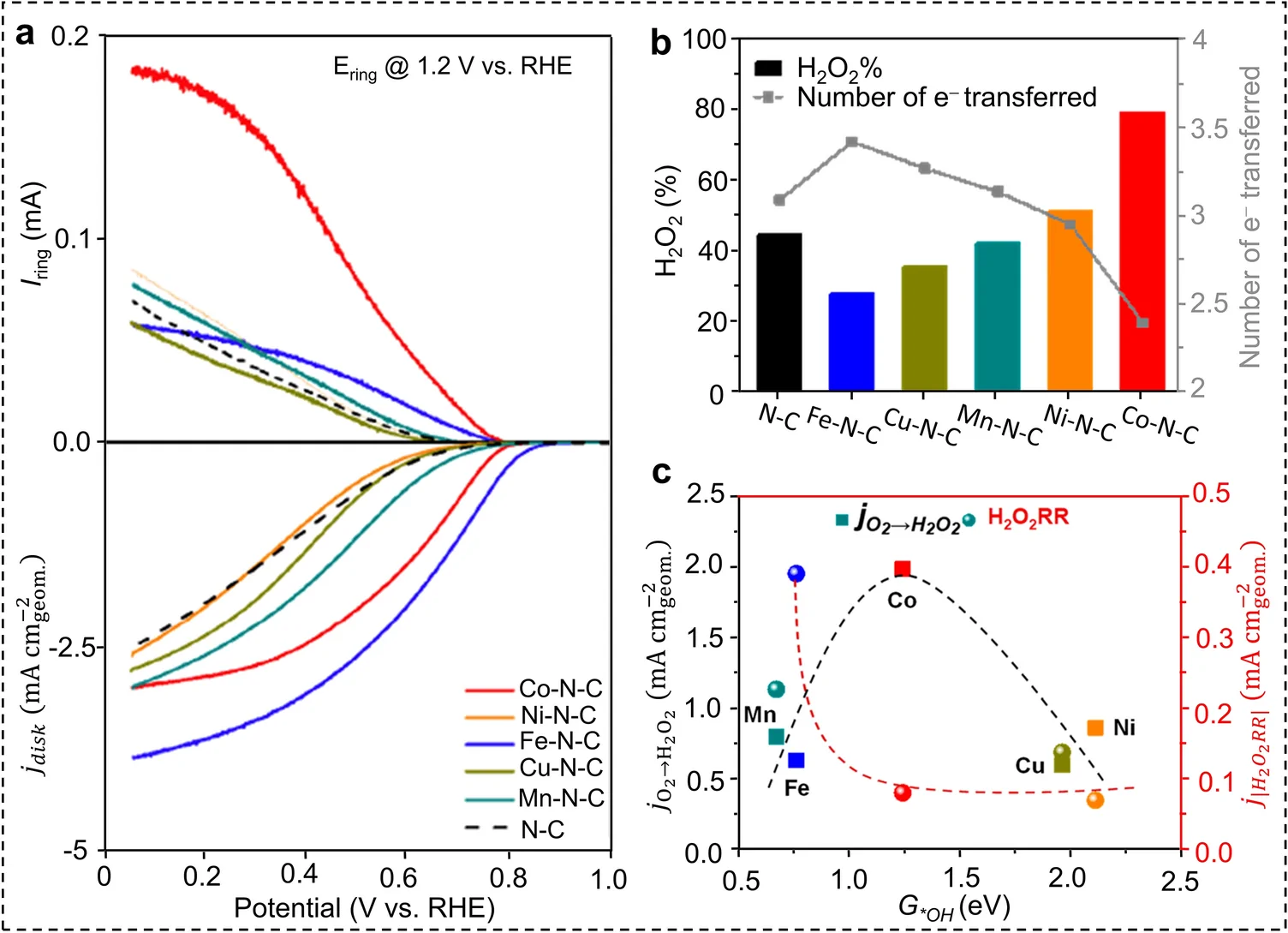
Engineering of metals on the 2e^–^ ORR performance of M–N–C catalysts. **a** LSV curves, **b** H_2_O_2_ selectivity and the number of e^–^ transferred, and **c** the current density of O_2_ to H_2_O_2_ and H_2_O_2_RR of the M–N–C catalysts [114]. Copyright 2019, American Chemical Society

As the active site of M–N–C catalysts, the type of metal directly affects its electrocatalytic properties. Strasser et al. [114] explored the relationship between the nature of 3*d* transition metals within a series of M–N–C (M = Mn, Fe, Co, Ni, and Cu) catalysts and ORR performance. They revealed that Co–N–C showed the highest ring current and H_2_O_2_ selectivity (almost 80%) toward 2e^–^ ORR for H_2_O_2_ production (Fig. 11a, b). They further concluded that, as the atomic number of the metal increases, the current density of O_2_ to H_2_O_2_ of the catalysts showed a volcano relationship, and the H_2_O_2_RR exhibited a sharp drop and then remained steady, and Co–N–C exhibits the optimal electrocatalytic performance for H_2_O_2_ production (Fig. 11c). Liu et al. [115] reached similar conclusions that the Mn–N–C and Fe–N–C bind strongly to the intermediates, resulting in O–O bond breaks to form H_2_O, whereas the Ni–N-C and Cu–N-C bind weakly, leading to less good reactivity. The Co–N–C has moderate binding energy to *OOH intermediates and is most favorable for 2e^–^ ORR.

**Fig. 12:**
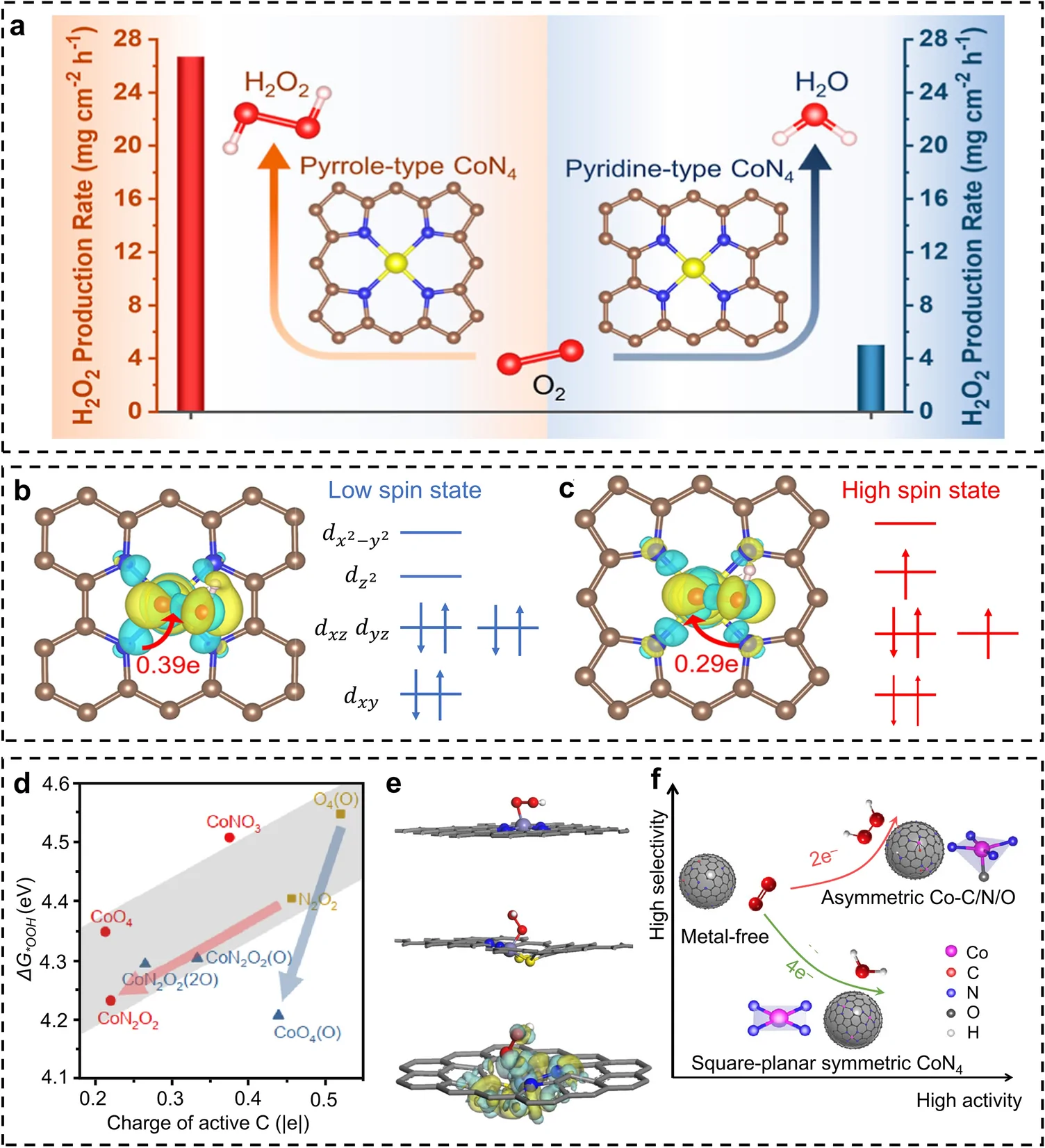
Engineering of coordination atoms on the 2e^–^ ORR performance of M–N–C catalysts. **a** Illustrative diagram of ORR performance of pyrrole-type CoN_4_ and pyridine-type CoN_4_ [85]. Copyright 2022, American Chemical Society. Differential charge distribution and 3d electron configuration of **b** pyridine-type CoN_4_ and **c** pyrrole-type CoN_4_ [85]. Copyright 2022, American Chemical Society. **d** Correlation of *OOH adsorption energies with the charge state of the active site (O-adjacent C atom) [150]. Copyright 2021, American Chemical Society. **e** *OOH adsorption mode on FeN_4_ and FeN_2_S_2_ and electron charge difference of *OOH adsorbed FeN_2_S_2_, with the gray, red, white, blue, yellow, and bluish-gray balls representing C, O, H, N, S, and Fe atoms, respectively [20]. Copyright 2024, Springer Nature. **f** Schematic illustration presents the H_2_O_2_ selectivity and ORR activity of centrosymmetric CoN_4_ and asymmetric Co–C/N/O electrocatalysts [155]. Copyright 2024, Springer Nature

The properties of coordination atoms can affect the electronic structure of the central metal, hence affecting the electrocatalytic performance [105, 138–141, 146–148]. Liu and coworkers reported that pyrrole-type CoN_4_ mainly promotes 2e^–^ ORR for H_2_O_2_ production while the pyridine-type CoN_4_ boosts 4e^–^ ORR (Fig. 12a) [85]. EXAFS fitting results demonstrated that the catalyst dominated by pyrrole-type CoN_4_ features a longer Co–N bond length (2.02 Å) than that of pyridine-type CoN_4_ (1.90 Å). This longer bond leads to weaker electronic interaction between Co and the *OOH intermediate, thereby achieving a high H_2_O_2_ selectivity of 94%. Theoretical calculations reveal that the pyrrole-type CoN_4_ site exhibits a less prominent electron transfer and a high-spin state as compared to pyridine-type CoN_4_ (Fig. 12b, c). As we mentioned previously, a high-spin state in metals can promote the transfer of local spin current and stabilize the *OOH intermediate, thus conducive to 2e^–^ ORR [84]. Yang et al. also reported that the increased Co spin state promotes the desorption of *OOH while preventing the cleavage of O–O bond, thus promoting the 2e^–^ ORR selectivity [149].

Qiao et al. disclosed that, compared to N coordination, O coordination can downshift the d-band center and Fermi level of the Co atom [150]. They revealed via wavelet transform XAFS (WT-XAFS) that the Co–O coordination bond is longer (2.01 Å) than the Co–N bond (1.89 Å), which weakens the binding interaction between Co and the adsorbed *OOH intermediate. Consequently, the active site was believed to migrate to the O-adjacent carbon atom due to the sluggishness of Co. Notably, the higher charge of active C caused the higher value of ∆G_*OOH_, indicating the weaker *OOH adsorption strength. Theoretical calculations revealed that CoN_2_O_2_ has optimal *OOH adsorption strength (Fig. 12d). Coincidentally, Zhao and Wang et al. revealed that the coordination of adjacent oxygen atoms alters the charge distribution of FeN_5−x_O_x_ sites, leading to a shift in the reactive sites from Fe atoms to the carbon atoms adjacent to oxygen. In the case of FeN_3_O_2_ sites, the carbon atom adjacent to oxygen plays a crucial role in binding the *OOH intermediate, thereby promoting the efficient production of H_2_O_2_ [151]. Waterhouse and Wang et al. demonstrated that substituting Co–N bonds with Co–O bonds can weaken the adsorption strength of *OOH, thereby enhancing the 2e^–^ selectivity, although it leads to a significant decrease in ORR activity [152]. Wang et al. reported that Fe–O–C, in sharp contrast to the well-known Fe–N–C for 4e^–^ ORR, is responsible for the 2e^–^ ORR pathway [153]. Zhang et al. constructed Ni-N_2_O_2_/C catalysts that showed excellent 2e^–^ ORR performance with 96% selectivity in 0.1 M KOH [154]. As a congeneric element, S possesses the same electronic regulatory effect as O. Meanwhile, because of its larger radius, it can further regulate the geometric structure of the catalyst, resulting in the distortion and deformation of chemical bonds. Li and coworkers disclosed that the replacement of two S atoms with N atoms in the FeN_4_ configuration will cause symmetry-breaking and electron redistribution (Fig. 12e). These geometric and electronic regulations lead to the strengthening of the O–O bond and the weakening of the Fe–O bond, thus being conducive to 2e^–^ ORR. Qiao and coworkers reported an O, S-dual coordinated Mo SACs that can catalyze ORR toward the 2e^–^ pathway with high selectivity above 95% in 0.1 M KOH [140].

In addition to in-plane coordination, axial coordination can also adjust the electronic structure of metal atoms, thereby regulating the catalytic performance. He and coworkers prepared a CoNCB catalyst with a configuration of Co–C/N/O (Fig. 12f), delivering an onset potential of 0.76 V and a 2e^–^ selectivity over 95%^.^[155]. Their computational studies observed a positive correlation between the enhanced ∆G_*OOH_ and the off-center distance of the Co atom. Moreover, they designed a series of CoNx-ACNT catalysts to correlate ORR selectivity with Co–N atomic coordination in another work [156]. The CoN_4+4_-ACNT catalyst exhibits the best-performing 2e^–^ ORR with an onset potential of 0.86 V and the potential of 0.82 V to reach the ring current density of 1 mA cm^–2^ in 0.1 M KOH, approaching the theoretical thermodynamic limit. Chen et al. revealed that the O axial coordination can modify the local electronic structure of the Co center in porphyrin (PC) molecules, which promotes its ∆G_*OOH_ closest to the peak of the volcano [157]. The CoPc-OCNT catalyst with the O-modified pyrrole-type CoN_4_ configuration achieves high ORR activity and 2e^–^ selectivity in both alkaline and neutral electrolytes, verifying the theoretical prediction. Gao et al. developed a Co-N_5_ site with an asymmetric electronic configuration, where breaking the symmetry of Co–N coordination promotes O_2_ activation and optimizes *OOH adsorption by disrupting the linear scaling relationship of intermediate binding, thereby enabling efficient H_2_O_2_ production and biomass upgrading [158]. Liang et al. demonstrated that the axial coordination structure (axial-OH) modulates the steric hindrance around the Ni-centers, thereby effectively enhancing its *OOH binding energy close to the optimum value (− 4.23 eV) [159]. Thus, the one-dimensional metal–organic framework Ni-tetra-aminobenzene catalyst exhibits excellent 2e^–^ ORR performance under both neutral and alkaline conditions. In another work, they constructed a -NH_2_ group axial coordinated Fe-based metal–organic frameworks anchored on aminated carbon nanotubes (Fe-BDC@CNT-NH_2_) to enhance H_2_O_2_ production [160]. They identified that, through the analysis and fitting XANES and Fourier-transformed EXAFS, the Fe species in Fe-BDC@CNT-NH_2_ exhibit a relatively low valence state (close to + 2) and form an Fe-NO_4_ coordination structure. This axial coordination structure and the low-energy state of Fe not only strengthen the adsorption capability of Fe sites toward *OH radicals, thereby effectively passivating the Fe sites, but also promote the 2e^–^ ORR performance of adjacent carbon sites.

**Fig. 13:**
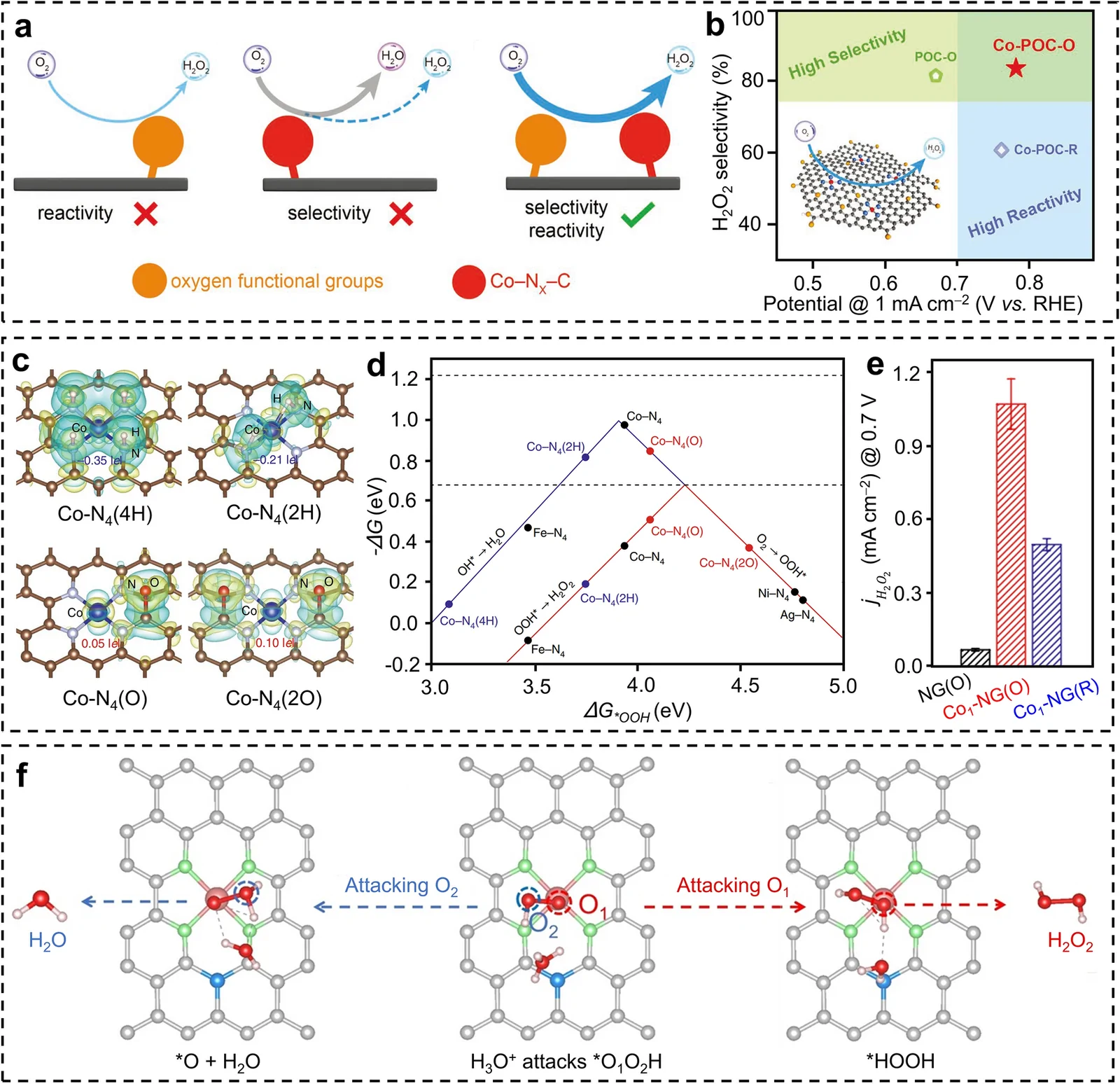
Engineering of functionalization at the carbon host on the 2e^–^ ORR performance of M–N–C catalysts. **a** Schematic illustration of the synergistic strategy involving atomic Co-Nx–C sites and oxygen functional groups for 2e^–^ ORR on non-noble-metal electrocatalysts [161]. Copyright 2019, Wiley–VCH. **b** Comparison of performance in terms of reactivity and selectivity for 2e^–^ ORR on Co-POC-O, Co-POC-R, and POC-O electrocatalysts, with the inset in panel **b** showing the mechanism scheme for the synergistic 2e^–^ ORR, and the black, blue, yellow, and red colors, respectively, correspond to C, N, O, and Co atoms [161]. Copyright 2019, Wiley–VCH. **c** Differential charge distribution of CoN_4_/graphene after 4*H, 2*H, *O, or 2*O was adsorbed near the cobalt atom, with the yellow and cyan isosurfaces (± 0.003 Bohr^−3^) indicating the electron gain and electron loss, respectively [4]. Copyright 2020, Springer Nature. **d** Catalytic activity volcanoes for the production of H_2_O (blue) and H_2_O_2_ (red) through the ORR (bottom panel), with the black data points represent M–N_4_/graphenes (M = Co, Ni, Fe, and Ag), which are utilized to construct the activity volcanoes, with the blue and red data points, respectively, represent CoN_4_/graphene with 4*H/2*H and *O/2*O adsorbed near the cobalt atom [4]. Copyright 2020, Springer Nature. **e** Comparison of H_2_O_2_ current (at 0.7 V) for NG(O), Co_1_-NG(O), and Co_1_-NG(R) [4]. Copyright 2020, Springer Nature. **f** Schematic diagram of the different attacking manner of H_3_O^+^ to *O_1_O_2_H intermediate and resultant product [164]. Copyright 2024, Wiley–VCH

The functionalization at the carbon host will cause the charge redistribution of the carbon host, thus regulating the electronic structure of the catalysts. As illustrated in Fig. 13a, Zhang et al. proposed that the Co-N_x_-C sites and oxygen functional groups contribute to the reactivity and selectivity for 2e^–^ ORR, respectively [161]. Control experiments confirmed that POC-O lacking Co-N_x_-C sites had low reactivity, and Co-POC-R lacking oxygen functional groups had low selectivity. As a combination, Co-POC-O catalysts showed both high reactivity and selectivity for 2e^–^ ORR (Fig. 13b). Hyeon et al. stated that the electronic state of Co in the CoN_4_ moiety turned more positive upon adsorption of electrophilic O atoms, but became more negative when electron donor H atoms were adsorbed (Fig. 13c) [4]. As a result (Fig. 13d), the *OOH adsorption energy of CoN_4_ slightly increases when an electrophilic O atom is adsorbed, approaching the optimal value of 4.22 eV for 2e^–^ ORR. It can be further increased when two O atoms are adsorbed, or decreased when electron donor H atoms are adsorbed. These predictions were further supported by experiments. The in situ O K-edge XAFS of Co_1_-NG(O) exhibits stronger and sharper characteristic peaks at 535 and 540.4 eV, corresponding to the transitions of the O 1* s* core level to the antibonding π* and σ* states of C–O bonds, respectively. This result directly confirms that the C–O–C epoxy groups retained under mild reduction conditions are adjacent to the CoN_4_ sites. In contrast, for Co_1_-NG(R), the aforementioned characteristic peaks are significantly weakened due to the desorption of C–O–C groups caused by high-temperature treatment. Additionally, the Co L-edge XAFS of Co_1_-NG(O) shows a downshift, which corresponds to the enhanced unoccupied state of the Co 3d-orbital in the low-energy region. This indicates that the electron density of Co atoms in Co_1_-NG(O) is lower than that in Co_1_-NG(R), verifying that the electron-withdrawing effect of the surrounding C–O–C functional groups renders the Co atoms electron-deficient. As a result, as shown in Fig. 13e, the Co_1_-NG(O) showed the highest current density for H_2_O_2_ generation, much surpassing the NG(O) and Co_1_-NG(R). These discoveries contribute to the comprehension of the essential relationship between the functionalization at the carbon host and the electrocatalytic performance of M–N–C catalysts.

Defects or edges can also alter the charge distribution of the carbon matrix, thereby regulating the ORR performance of the catalysts. Zhang and coworkers reported that the edge-hosted atomic CoN_4_ sites are more favorable for 2e^–^ ORR than the basal-plane-hosted ones [162]. Besides, Shi et al. discovered via in situ XAFS that the edge-hosted architecture adapts dynamic oxo-adsorption and valence state shuttling between Co^(2−δ)+^ and Co^2+^, as opposed to the rigid in-plane embedded Co_1_-N_x_ counterpart [163]. Theoretical calculations revealed that the synergistic interplay between the in situ reconstructed Co_1_-N_2_-oxo and peripheral oxygen groups causes near-optimal *OOH adsorption and markedly raises the activation barrier for its dissociation, giving rise to a strong acidic ORR activity and 2e^–^ selectivity.

The functionalization can also regulate the local environment of the active center to adjust the catalytic performance. Liu et al. discovered that boron heteroatoms in the second coordination sphere of CoN_4_ (Co_1_-NBC) heighten the proton affinity on the catalyst surface, facilitating proton attack on the former oxygen of *OOH and thus promoting H_2_O_2_ production (Fig. 13f) [164]. As a result, Co_1_-NBC manifests prominent 2e^–^ ORR activity and selectivity in acid, with an onset potential of 0.724 V vs. RHE and 94% H_2_O_2_ selectivity in 0.1 M HClO_4_. Cao et al. reported the steric effect on ORR via Co porphyrin atropisomers in tetrahydrofuran with decamethylferrocene [165]. They revealed that the ORR rate of the catalyst is positively correlated with the steric hindrance. The highest kinetics is achieved when the four groups are in the same direction. Yang and coworkers developed a strategy to specifically regulate oxygen functional groups (OFGs) to enhance H_2_O_2_ selectivity up to 92% in acidic conditions using cobalt porphyrin molecules assembled with reduced graphene oxide [166]. Their research revealed that different OFGs represent distinct interactions on the Co center via either remote (C–O–C and –COOH) or vicinal (–OH) interaction modes, thereby inducing different degrees of electron deficiency around the Co center. The optimal OFG is determined to be the C–O–C group. These distinct interaction mechanisms result in contrasting effects on the promotion of 2e^–^ ORR selectivity. Moreover, Xia et al. presented an effective strategy for containing sulfonic acid groups that anchor active Fe sites [167]. This modification was proved to facilitate the retention of Fe units and protect against radical damage for maintaining well-crafted three-phase microenvironments. Consequently, the modified catalysts achieved a high Faraday efficiency exceeding 90% for H_2_O_2_ electroproduction via 2e^–^ ORR.

#### Main-Group-Metal Catalysts

Main-group-metal catalysts represent a unique and increasingly researched category. These catalysts are more abundant and less toxic compared to transition metals. However, main-group metals typically have filled s-orbitals and partially filled p-orbitals, leading to less flexibility in coordination and oxidation states. These characteristics result in fewer applications for catalytic processes, particularly for ORR, where variable electronic states and coordination flexibility are beneficial. Thus, the precise control of main-group metals for optimizing their 2e^–^ ORR performance has been challenging until recent materials advancements.

**Fig. 14:**
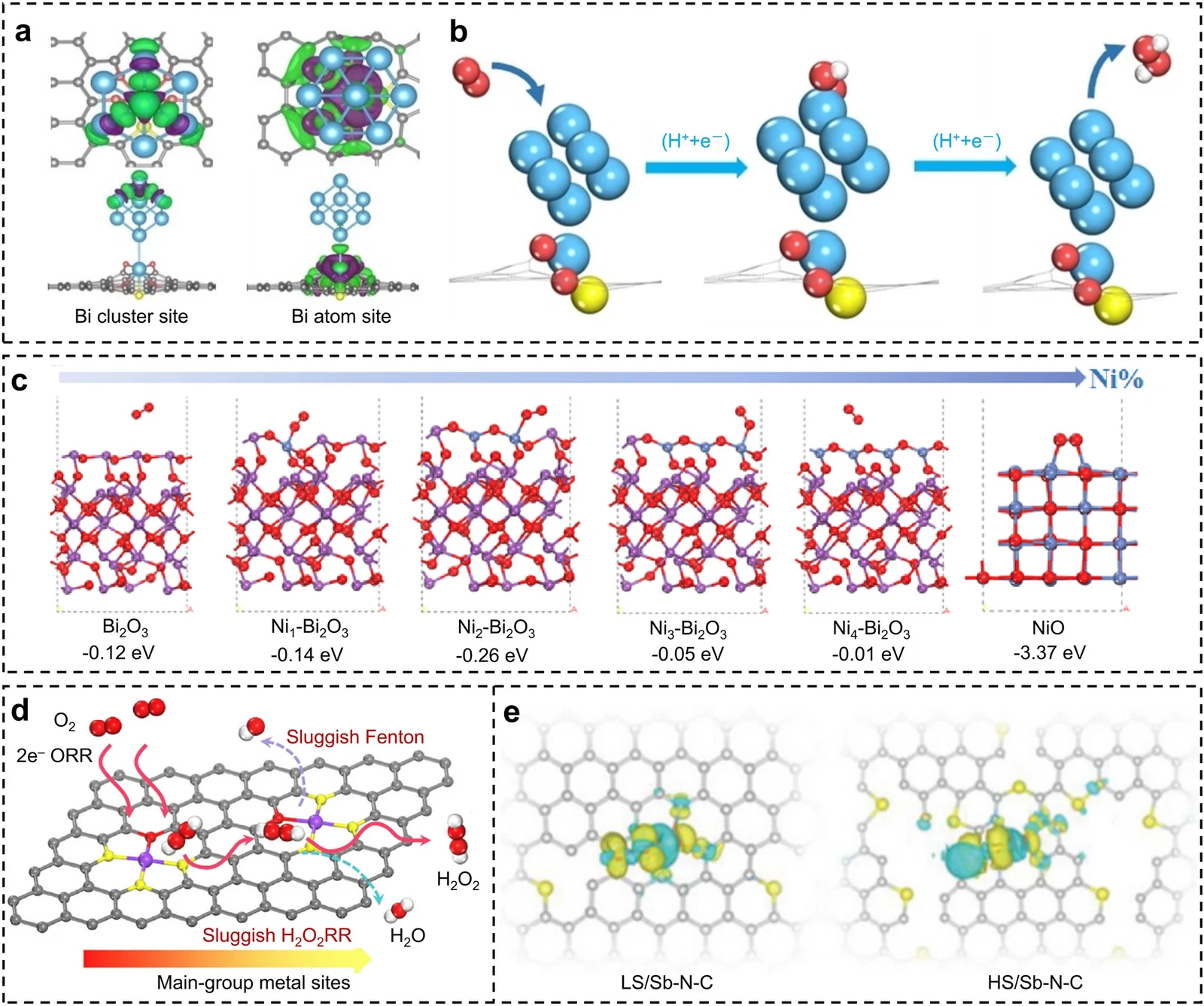
Engineering of main-group-metal catalysts for 2e^–^ ORR. **a** Electron charge difference of Bi cluster sites (left) and Bi single-atom site (right) [89]. Copyright 2023, Wiley–VCH. **b** Diagram of the optimal models for 2e^–^ ORR reaction step at Bi cluster site of BiOS_SA_/Bi_clu_, with the water blue, yellow, red, gray, white balls represent Bi, S, O, C, and H atoms, respectively [89]. Copyright 2023, Wiley–VCH. **c** Optimized structures of the O_2_ molecule on Bi_2_O_3_, Ni_X_-Bi_2_O_3_, and NiO, with the purple, blue, and red balls representing Bi, Ni, and O atoms, and the values are representative of the O_2_ physisorption energy [91]. Copyright 2023, Royal Society of Chemistry. **d** Schematics of the electrochemical cumulative H_2_O_2_ production on the main-group metal catalysts [173]. Copyright 2023, Springer Nature. **e** Electron charge difference of LS/Sb-N–C and HS/Sb-N–C, with the yellow and cyan representing the electron gain and loss, respectively [88]. Copyright 2024, Wiley–VCH

Li et al. disclosed that metallic Bi nanoparticles and the single-atomic-site Bi demonstrate outstanding performance for 2e^–^ and 4e^–^ ORR, respectively [90]. They performed crystal orbital Hamilton population (COHP) analysis and discovered that the absolute values of the integrated COHP of O–O bonds from adsorbed *OOH hybridization up to the Fermi level on the Bi-102 surface are greater than those of the other main-group p-block metals. This suggests a stronger O–O binding and a higher *OOH reduction barrier on Bi, resulting in a higher performance for 2e^–^ ORR. The Chen group's work also reported and explained this phenomenon. They presented a main-group Bi catalyst, BiOS_SA_/Bi_clu_, in which single-atomic Bi sites coordinated with O and S coexist with Bi nanoclusters. This catalyst enables highly selective ORR via the 2e^–^ pathway [89]. Poisoning experiments and theoretical calculations reveal that the catalytically active sites of BiOS_SA_/Bi_clu_ are on the Bi clusters rather than the Bi single-atom sites. The electron charge difference in Fig. 14a indicates that the electron gain or loss of the cluster site is balanced, while electrons are transferred from the single-atom site to the coordinated nonmetallic atoms. Moreover, the oxygen species are adsorbed on the top of the cluster, which has an appropriate ∆G_*OOH_ and exhibits the lowest overpotential for 2e^–^ ORR (Fig. 14b).

Regarding Bi catalysts, Wang et al. prepared a series of BiNiO_x_ with various crystalline surfaces by changing the Bi content [91]. Among them, the BiNiO_*x*__−4_, which has a moderate Bi content, shows the highest H_2_O_2_ selectivity, reaching 93.2% in 0.1 M KOH. Through DFT calculations, they demonstrated that the O_2_ adsorption configuration and strength can be flexibly adjusted by modulating Bi content, thereby regulating the ORR performance for H_2_O_2_ production. As shown in Fig. 14c, the O_2_ is “end-on” adsorbed on the Ni_3_-Bi_2_O_3_ surface with moderate strength (-0.05 eV). In contrast, it is over-strongly “side-on” adsorbed on the Bi_2_O_3_ (– 0.12 eV) and NiO (– 3.37 eV). Besides, Peng et al. constructed BiNi alloys supported on carbon nanosheets by a hydrothermal pyrolysis method [168]. The BiNi/C exhibited a high selectivity of ~ 98% with an onset potential of 0.76 V versus RHE in 0.1 M KOH. Theoretical calculations revealed that in BiNi alloys, the Bi atoms are geometrically isolated and create an electron-deficient shell around the Ni sites. This unique structure weakens the adsorption of *OOH, thus enhancing the selectivity of the 2e^–^ ORR. Additionally, Wang and coworkers developed Sn-BiOCl nanosheet catalysts to accelerate 2e^–^ ORR, achieving an impressive yield rate of 10,628 mg L^–1^ h^–1^ in 0.5 M Na_2_SO_4_ [169]. They revealed that the introduction of Sn elements modulates the electronic structure and morphological features of BiOCl, enhancing *OOH adsorption and activation, thereby promoting the 2e^–^ ORR process. Zhu and coworkers prepared oxygen-vacancy-enriched Bi_2_O_3_ nanorods (Ov-Bi_2_O_3_-EO) via electrochemically oxidatively reconstructing the Bi-MOF nanorod precursor, acting as efficient dual electrocatalysts for anodic and cathodic reactions, enabling concurrent H_2_O_2_ production at both electrodes with 150% Faradaic efficiency [170]. They revealed that oxygen vacancies are key to optimizing intermediate adsorption in selective 2e^–^ pathways, thus enhancing the electrocatalytic activity and selectivity.

Li et al. developed an approach for accurately synthesizing an optimal catalyst for 2e^–^ ORR based on a heteroatom-modified main-group-metal–organic framework [171]. Their first-principles calculations revealed that the adjustment of the coordination atom and functionalization at the carbon host can modify the electronic state of the In center to regulate the *OOH adsorption strength. The In-N_3_SB(B-S) model showed the optimal adsorption energy, which was favorable for 2e^–^ ORR. Guided by theoretical predictions, they prepared a main-group-metal In anchored through hollow carbon rods with N, S-dual first, and B second coordination (In SAs/NSBC). The In SAs/NSBC exhibits a 2e^–^ selectivity of above 95% within a broad pH range. In another study, an In single atom decorated N, O mesoporous carbon with P dopants occupied the second coordination sphere catalyst (In-N_2_O_2_-P-mC) was developed for efficient electrosynthesis of H_2_O_2_ in neutral media [172]. Comprehensive analysis revealed that the P dopants regulate the electronic state of the In site, moderating its *OOH binding strength for an enhanced 2e^–^ ORR kinetic.

Yu and coworkers reported a main-group Pb SAC with S and O super-coordination [173]. As shown in Fig. 14d, the main-group metal site can boost 2e^–^ ORR, meanwhile suppressing H_2_O_2_RR and Fenton reactions. Theoretical simulations revealed that the S and O super-coordination guides a portion of electrons from the main-group Pb sites to the coordinated oxygen atoms, thereby optimizing the *OOH binding energy and enhancing the ORR activity and 2e^–^ selectivity. In a recent study, Guo et al. reported *p*-block Sn single-site catalysts with oxygen-modified Sn sites, where O–Sn interactions regulate the local electronic structure for efficient, scalable H_2_O_2_ synthesis [174]. Using Sn_1_/C(O) as the cathodic catalyst, the electrolyser delivers 300 mA cm^–2^ at a cell voltage of 1.17 V, achieving an energy efficiency of 43% and stable operation for over 200 h. Fei et al. reported that the main-group Sb-N–C SAC with high sulfur content (HS/Sb-N–C) followed a 2e^–^ pathway with high selectivity (96.8%), while the sister catalyst with low sulfur content (LS/Sb-N–C) directed a 4e^–^ pathway [88]. The electron charge difference pattern in Fig. 14e indicated that a more significant electron transfer occurred from the Sb-N_4_ of HS/Sb-N–C to the *OOH intermediate compared to LS/Sb-N–C. This suggests that the additional charge might potentially occupy the antibonding orbitals of the *OOH intermediate, leading to weakened absorption.

### Metal-Free Carbon Catalysts

Metal-free carbon catalysts have emerged as a versatile alternative in the field of 2e^–^ ORR [175–185]. Their unique structural and electronic properties make them ideal candidates for achieving enhanced ORR activity through geometric and electronic engineering [5, 24, 27, 54, 186–190]. This section explores the advances in carbon catalysts, focusing on how strategic engineering has led to significant improvements in their 2e^–^ ORR performance.

#### Pristine Carbons

**Fig. 15:**
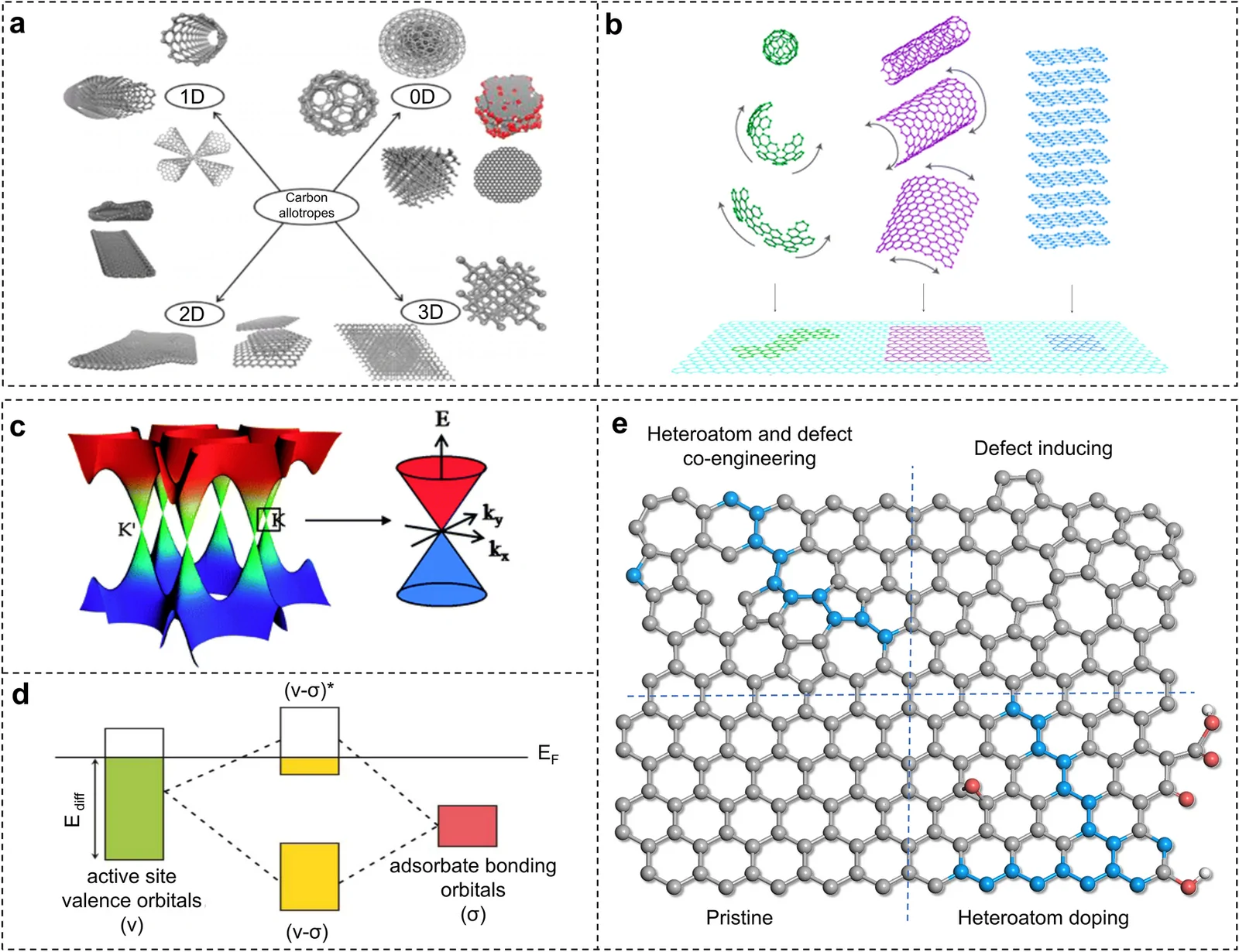
The nature of carbons. **a** Schematic illustration of the geometric structure diversity of carbon materials [191]. Copyright 2015, American Chemical Society. **b** 2D building material for carbon materials of all other dimensionalities [192]. Copyright 2007, Springer Nature. **c** Electronic band structure of single-layer graphene [195]. Copyright 2009, Royal Society of Chemistry. **d** Scheme of orbital hybridization in the valence band from active sites and the bonding orbital of adsorbates, with the E_F_ referring to the energy of the highest valence orbital of the entire graphene cluster [200]. Copyright 2014, American Chemical Society. **e** Schematic diagram of the engineering of carbon materials

According to the hybrid orbital theory, carbon atoms can form covalent bonds with other carbon atoms or nonmetallic elements in various hybridizations such as *sp*, *sp*^2^, or *sp*^3^. This enables them to constitute a broad range of structures, ranging from zero-dimensional (0D) to three-dimensional (3D), as shown in Fig. 15a [191]. Despite the structural diversity, carbon materials have a unity. For example, 0D fullerenes, 1D carbon nanotubes, and 3D graphite can all be regarded as resulting from the wrapping, rolling, or stacking of parts of a 2D honeycomb lattice (Fig. 15b) [192]. This flat monolayer of carbon atoms tightly packed into a 2D matrix is named graphene, which has been widely used for describing the properties of various carbon materials [192–195]. Graphene is a unique zero-band-gap semimetal material whose Fermi surface is located at six points where the conduction band intersects the valence band, as shown in Fig. 15c [195]. This special band structure gives graphene unique and excellent electrical performance, such as alterable electronic structures (Fig. 15d) [196–198]. The pristine graphene interacts more weakly with oxygen species owing to its electrical neutrality, which has low electrocatalytic reactivity for ORR [199]. Through methods such as heteroatom doping, defect inducing, and their coengineering, as shown in Fig. 15e, the pristine carbon can be modified to regulate the geometric and electronic structures, thereby improving the catalytic ORR performance.

#### Heteroatom-Doped Carbons

Heteroatom doping is the replacement of partial carbon atoms in the carbon lattice or modification of the carbon edges by other atoms. The different electronegativity and size will induce the regulation of electronic and geometric structures, leading to the regulation of electrocatalytic properties [201–207]. According to the relative electronegativity of heteroatoms and carbon, doping can be divided into p-type doping and n-type doping.

In p-type doping, electron-deficient heteroatoms such as B are introduced into the carbon lattice, which consequently causes a downshift of the Fermi level of the carbon framework [25, 208–211]. This will cause further weak adsorption of reaction intermediates on carbon; hence, the active site migrates to the heteroatoms. Wang and coworkers prepared a series of doped carbon and found that the B-doped carbons show enhanced H_2_O_2_ selectivity than pure carbon [210]. They revealed through DFT calculations that oxygen species tend to be adsorbed at the B site rather than the carbon sites. They further performed ab initio molecular dynamics (AIMD) to model the reaction kinetics and found that the breaking of the *–OOH bond has a lower free-energy barrier than the breaking of the *O–OH bond at the B site, leading to the formation of H_2_O_2_.

**Fig. 16:**
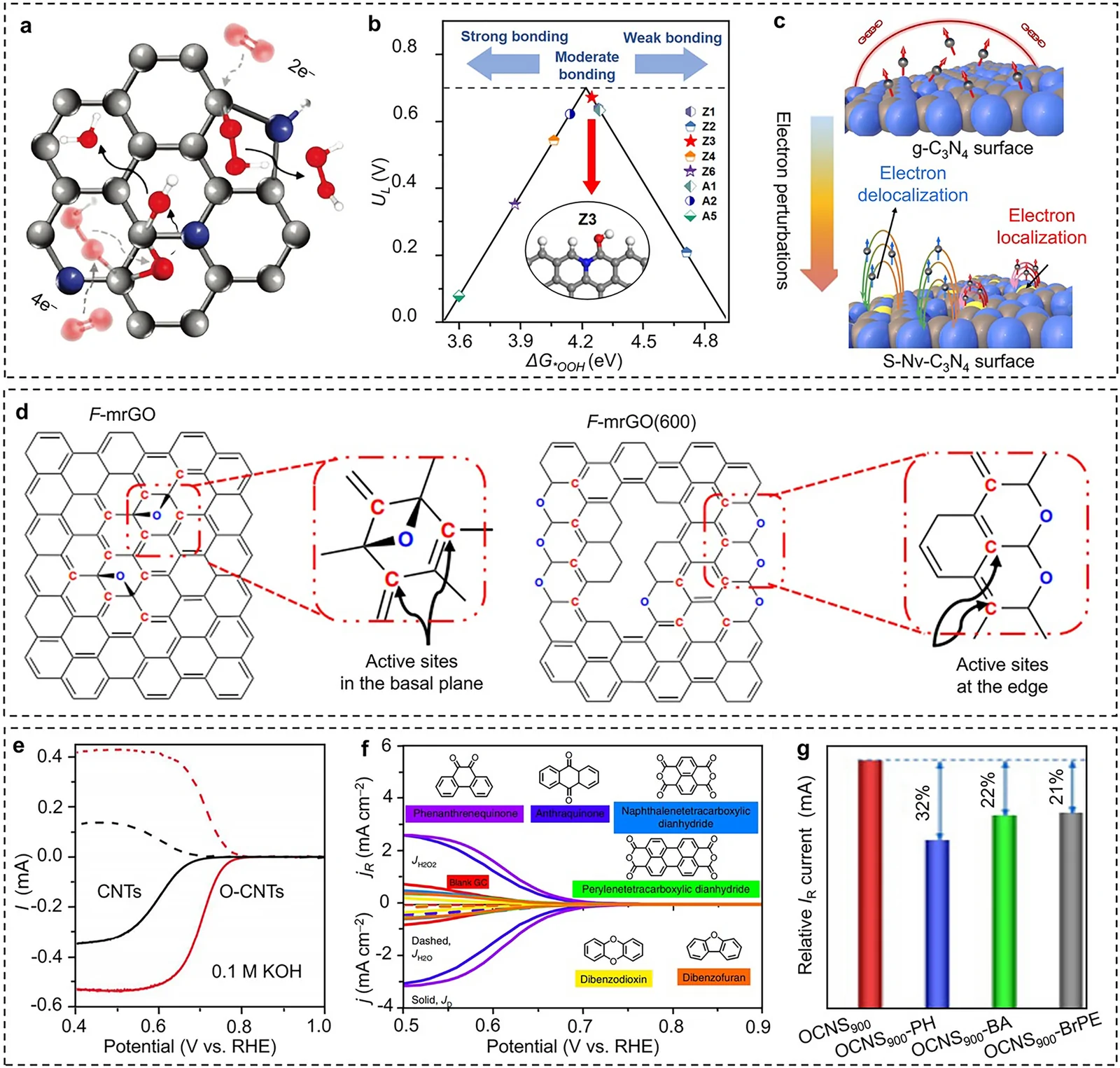
Engineering of heteroatom-doped carbon materials for 2e^–^ ORR. **a** Schematic diagram of 2e^–^ and 4e^–^ ORR pathways on different nitrogen configurations [213]. Copyright 2020, Wiley–VCH. **b** Volcano plots with the limiting potential plotted as a function of ΔG_*OOH_ [215]. Copyright 2024, Wiley–VCH. **c** Schematic diagram of in-plane electron redistribution [216]. Copyright 2024, Wiley–VCH. **d** Schemes of proposed active sites on *F*-mrGO and* F*-mrGO(600) [6]. Copyright 2018, Springer Nature. **e** ORR polarization curves of the O-CNTs and CNTs [5]. Copyright 2018, Springer Nature. **f** ORR polarization curves of standalone molecules [186]. Copyright 2020, Springer Nature. **g** Relative *I*_R_ at 0.55 V (vs. RHE) current for different catalysts [219]. Copyright 2021, Wiley–VCH

For n-type doping, electron-rich heteroatoms (e.g., N, O, P, S) are incorporated into the carbon lattice, which induces an upshift of the carbon matrix Fermi level. Nakamura et al. characterized the ORR active site of N-doped carbon materials [212]. They revealed that carbon atoms located adjacent to pyridinic-N were proved to be turned to Lewis basicity and be the active sites of N-doped carbon for ORR. However, the lone pairs from the pyridinic-N were claimed to promote the charge transfer from the π orbital to the antibonding orbitals in O_2_, leading to the weakening of O–O bond thus conducive to the breaking of O–O bond to form H_2_O [213, 214]. Through a simple adjustment of the N dopants categories, the ORR pathway can be precisely regulated. Qiao and coworkers found that, unlike the typical 4e^–^ active sites generated by pyridinic-N dopants, pyrrolic-N dopants produce the highly selective 2e^–^ ORR active sites (Fig. 16a) [213]. They precisely regulated the content of pyrrolic-N by adjusting the mass ratio of the precursor materials. Remarkably, the catalyst with a high amount of pyrrolic-N exhibits high H_2_O_2_ selectivity over 95% in 0.1 M KOH. Besides, the adsorption properties of the active sites can be regulated more flexibly through the synergistic effect of multiple element dopants. As shown in Fig. 16b, Lu et al. reported that individual graphitic-N dopants exhibit weak bonding to *OOH. When it cooperates with hydroxyl (–OH) groups, the *OOH adsorption strength of active sites is enhanced to a moderate degree that facilitates the reaction of *OOH intermediate with proton to form H_2_O_2_ [215]. In addition, Zhang and coworkers discovered that the introduction of S on the g-C_3_N_4_ surface brings ferromagnetic perturbation to carbon active centers, triggering electron delocalization and optimizing *OOH adsorption for H_2_O_2_ production (Fig. 16c) [216]. Wang group reported a N, B, O-codoped carbon nanosheet assembled by nitrogen-doped carbon quantum dot (N-CQD) via a hydrothermal-ball milling tandem strategy. Their DFT calculations revealed that the N-B-O cross-linker formed via dehydration of N-CQDs triggers substantial electronic redistribution across the structure, thereby boosting the activation of inner carbon atoms and electron transfer efficiency. In situ ATR-FTIR and Raman characterizations demonstrated that N, B, O-codoped carbon nanosheets strengthen O_2_ adsorption and stabilize key intermediates (*OOH, *HOOH), which boost the 2e^–^ ORR performance [217]. As a congener element, P shares the same valence electron configuration as N and can also serve as an effective dopant for carbon-based materials. Xia et al. synthesized phosphorus-doped carbon nanotubes (P-CNTs) via a hydrothermal method [221]. This catalyst exhibits excellent performance in the H_2_O_2_ electrosynthesis in acidic and neutral media, achieving a cumulative H_2_O_2_ concentration of 1291.3 mg L^–1^ h^–1^ with a faradaic efficiency of 88.5%. Zhou et al. reported a P-doped carbon-based catalyst via the carbonization of ethylenediaminetetramethylene phosphonic acid [222]. This catalyst exhibits exceptional 2e^–^ ORR performance in Cl^–^-bearing electrolytes such as seawater. Theoretical calculations revealed that the introduction of P enables preferential adsorption of Cl^–^ on the catalyst surface, thereby promoting the reduction of O_2_ to H_2_O_2_.

Similar to N dopants, O can also turn the adjacent carbon atoms to Lewis basicity, generating an active site for ORR [27, 177]. McCloskey presented a selectively and efficiently metal-free O-doped carbon electrocatalyst for 2e^–^ ORR, obtained via a scalable and mild thermal reduction of graphene oxide [6]. As shown in Fig. 16d, spectroscopic structural characterization and in situ electrochemical Raman offer robust evidence that the C–O–C located at the graphene sheet edge are the most active sites for peroxide production. Coincidentally, Cui et al. reported a series of O-functionalized carbon materials prepared through a nitric acid treatment [5]. Typically, as shown in Fig. 16e, oxidized carbon nanotubes (O-CNTs) exhibit enhanced ORR activity and H_2_O_2_ selectivity compared to pristine CNTs. Through DFT calculation and controlled experiments, they further discovered that –C–O–C and –COOM functional groups are active and selective for 2e^–^ ORR. Baek et al. constructed graphitic nanoplatelets with dangling edge sites as the model catalysts and decorated them with targeted functional groups to identify the highly active oxygenated groups in O-doped carbon materials for 2e^–^ ORR [186]. They revealed through experiments combined with theoretical calculations that the quinone (–C=O) group is the most active and selective. They further tested the ORR performance of several standalone molecules with different groups, as shown in Fig. 16f, verifying this conclusion. Joo et al. [218] and Liu et al. [219] reported a method of molecular-specific blocking, which is capable of shielding specific oxygen functional groups, thereby identifying their catalytic activities. They all indicated that the 2e^–^ ORR activity of the catalyst decreased to a greater extent after blocking the carbonyl (–C=O) groups, thus identifying it as the active center (Fig. 16g). Jiang and coworkers revealed that aldehyde (–CHO) group functionalized carbon quantum dots (CQDs) presented highest H_2_O_2_ electrosynthesis efficiency surpassing the hydroxyl (–OH), and carboxyl (–COOH) groups. Their experimental and theoretical results highlight the critical role of electron-withdrawing groups in accelerating charge transfer kinetics, thus boosting H_2_O_2_ electrosynthesis efficiency [220].

Except for electronic redistribution, dopants can also cause structural distortion of the carbon skeleton due to the difference in atomic size between heteroatoms and carbons. For instance, the S atom has a similar electronegativity to carbon, but the doped carbon nanomaterials also show enhanced ORR activity and 2e^–^ selectivity compared to the pristine ones [26, 223–225]. Lu et al. demonstrated that S can induce carbon framework structural distortion, thereby optimizing the *OOH adsorption strength and enhancing the performance of H_2_O_2_ electrosynthesis [26]. Vasudevan et al. synthesized a sulfur-doped carbon chain network nanomaterial via a flame soot deposition method. Endowed by sulfur doping, this material exhibits exceptional electrocatalytic activity and stability for the 2e^–^ ORR in acidic media [223]. Tong et al. constructed a Zn–air battery integrated system using the p-type semiconductor polyterthiophene as a photo-enhanced photoelectrocatalyst for the 2e^–^ ORR. This system achieves the integration of electricity and H_2_O_2_ cogeneration with wastewater treatment, delivering an H_2_O_2_ yield of 34.8 mg L^–1^ h^–1^ and a rhodamine B (RhB) removal rate approaching 100% [224]. Che et al. reported a hollow porous carbon sphere-sulfur composite for efficient H_2_O_2_ production in alkaline solution [225]. Their experimental results revealed that, upon S incorporation, the H_2_O_2_ selectivity is enhanced from 20% to over 70%, accompanied by a H_2_O_2_ yield rate of 183.99 mmol·g_cat_^–1^ and a corresponding Faradaic efficiency of 70%. Theoretical calculations demonstrated that the formation of S–S bonds remarkably reduces the overpotential, thereby leading to a substantial enhancement in electrocatalytic performance.

#### Defect-Induced Carbons

While heteroatom doping efficiently modulates the electronic structure and intermediate adsorption behavior of carbon materials, its regulatory capacity is inherently constrained by the type, content, and coordination configuration of dopant atoms. In contrast, defect-rich carbon materials with intrinsic structural defects serve as a versatile alternative. Through precise tailoring of defect types and densities, these materials enable flexible modulation of charge distribution and surface physicochemical properties, thus opening up new avenues for optimizing electrocatalytic performance.

Compared to in-plane carbon atoms, the carbon atoms adjacent to edge carbons were found that have a higher charge density, which is conducive to proceeding with ORR [27, 226]. Lin and coworkers disclosed the nature of carbon edges for H_2_O_2_ production [227]. They revealed that all armchair defects, zigzag defects, and armchair/zigzag composite defects were proved to shrink the HOMO and LUMO energies of the molecules for boosting 2e^–^ ORR performance. By using time-resolved infrared spectroscopy and simulation calculations, they observed that the key intermediate equilibrated more quickly at the zigzag edges compared to the armchair edge.

**Fig. 17:**
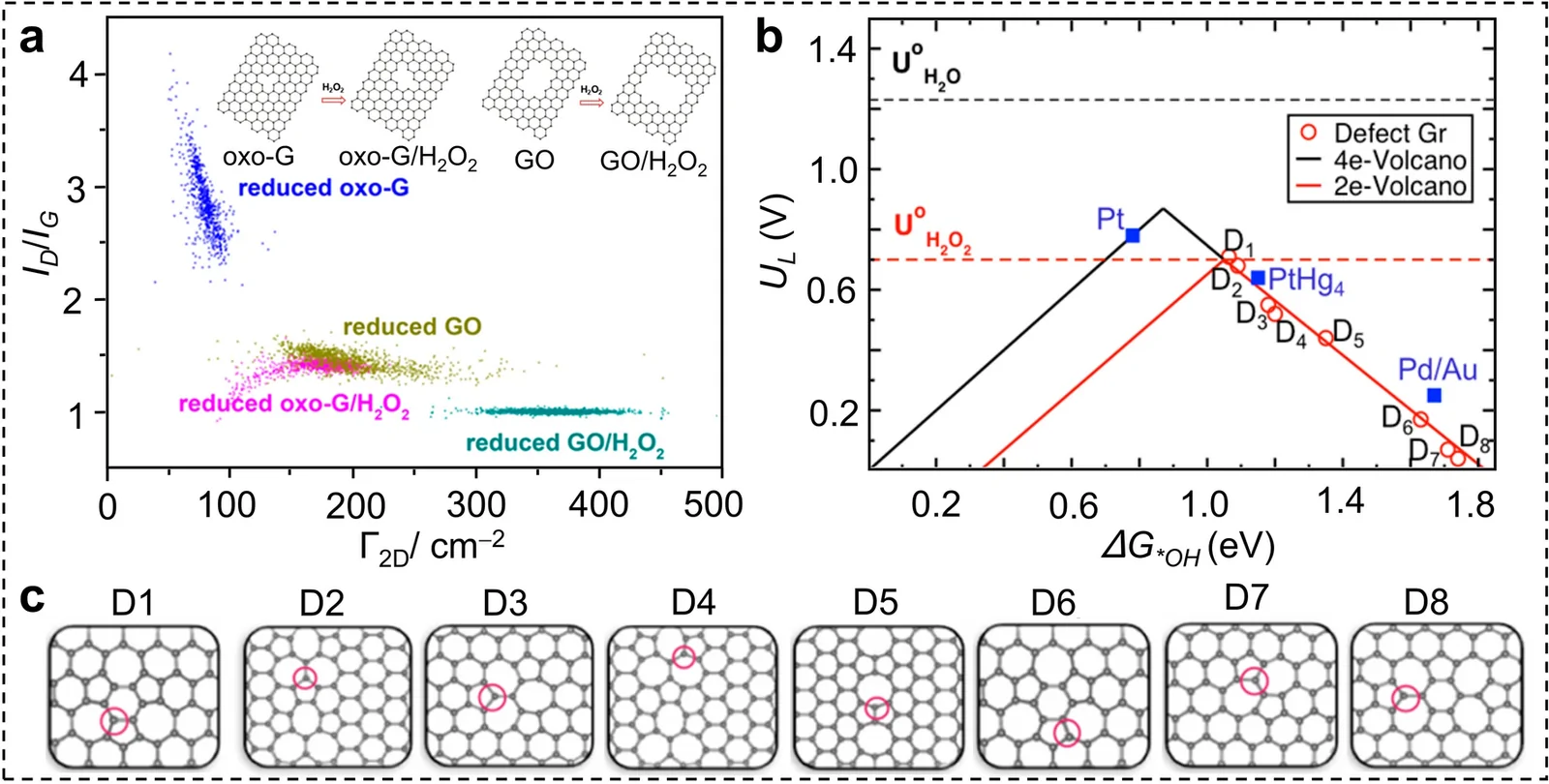
Engineering of defect-induced carbon materials for 2e^–^ ORR. **a** Statistical Raman spectroscopy (SRS) of samples deposited on a 300 nm SiO_2_/Si wafer. This plot demonstrates the functionalization of the reduction of oxo-G, oxo-G/H_2_O_2_, GO, and GO/H_2_O_2_ by hydroiodic acid and trifluoroacetic acid. Γ_2_D represents the full width at half-maximum of the 2D band. The inset shows the scheme of the preparation procedure for GO precursors with different densities of in-plane lattice defects [228]. Copyright 2019, American Chemical Society. **b** Two-electron (red) and four-electron (black) ORR-related volcano plots with the limiting potential plotted as a function of ΔG_*OH_ at **c** different carbon defect type configurations [178]. Copyright 2018, American Chemical Society

In-plane carbon lattice defects can also promote the redistribution of electronic structures. Eigler and Strasser et al. reported various graphene precursors with different in-plane carbon lattice defects from oxo-functionalized graphene (oxo-G) and graphene oxide (GO) with H_2_O_2_ hydrothermal treatment [228]. As shown in Fig. 17a, statistical Raman spectroscopy analysis revealed that the in-plane carbon lattice defect density increased in the following order: oxo-G, oxo-G/H_2_O_2_, GO, GO/H_2_O_2_. Furthermore, they prepared nitrogen-doped graphene materials by subjecting those graphene precursors to hydrothermal treatment with ammonium hydroxide. The electrochemical measurement results reveal that the nitrogen-doped graphene derived from oxo-G, which had the lowest in-plane carbon lattice defects, displayed the highest H_2_O_2_ selectivity of over 82% in 0.1 M KOH. Bao et al. studied the effect of pore size on the electrocatalytic performance of carbon materials [178]. Both MicroC and MesoC showed activity and selectivity toward 2e^–^ ORR, while the highly oriented pyrolytic graphite (HOPG) exhibited non-activity for oxygen reduction. Furthermore, they used DFT calculations to uncover the critical role of defects, including point defects such as 5–8-5, 555–777, 555–6-777, 5555–6-777 double vacancies, and line defects 555–777, 55–8-55 defects, as well as topological defects such as 55–77 Stone–Wales defects, pentagon, hexagon, heptagon, and octagon defects (Fig. 17b, c). The majority of them are naturally selective for the 2e^–^ pathway toward H_2_O_2_ from O_2_, and the 555–777 defect is located near the top of the volcano, suggesting its optimal adsorption strength for efficient 2e^–^ ORR.

#### Heteroatom and Defect-co-Engineered Carbons

In practice, the heteroatoms and defects in carbon materials usually coexist or are even interdependent with each other, meaning that the geometric and electronic structures of the carbon matrix are influenced by both of them. The performance of the catalyst can be regulated by the combination of various heteroatoms and defects [229]. Theoretically, this two-way regulation is more flexible and varied than the single one. Moreover, the optimal design of nanocarbon via favorable heteroatom dopants at the specific defect sites can achieve higher ORR activity [199].

**Fig. 18:**
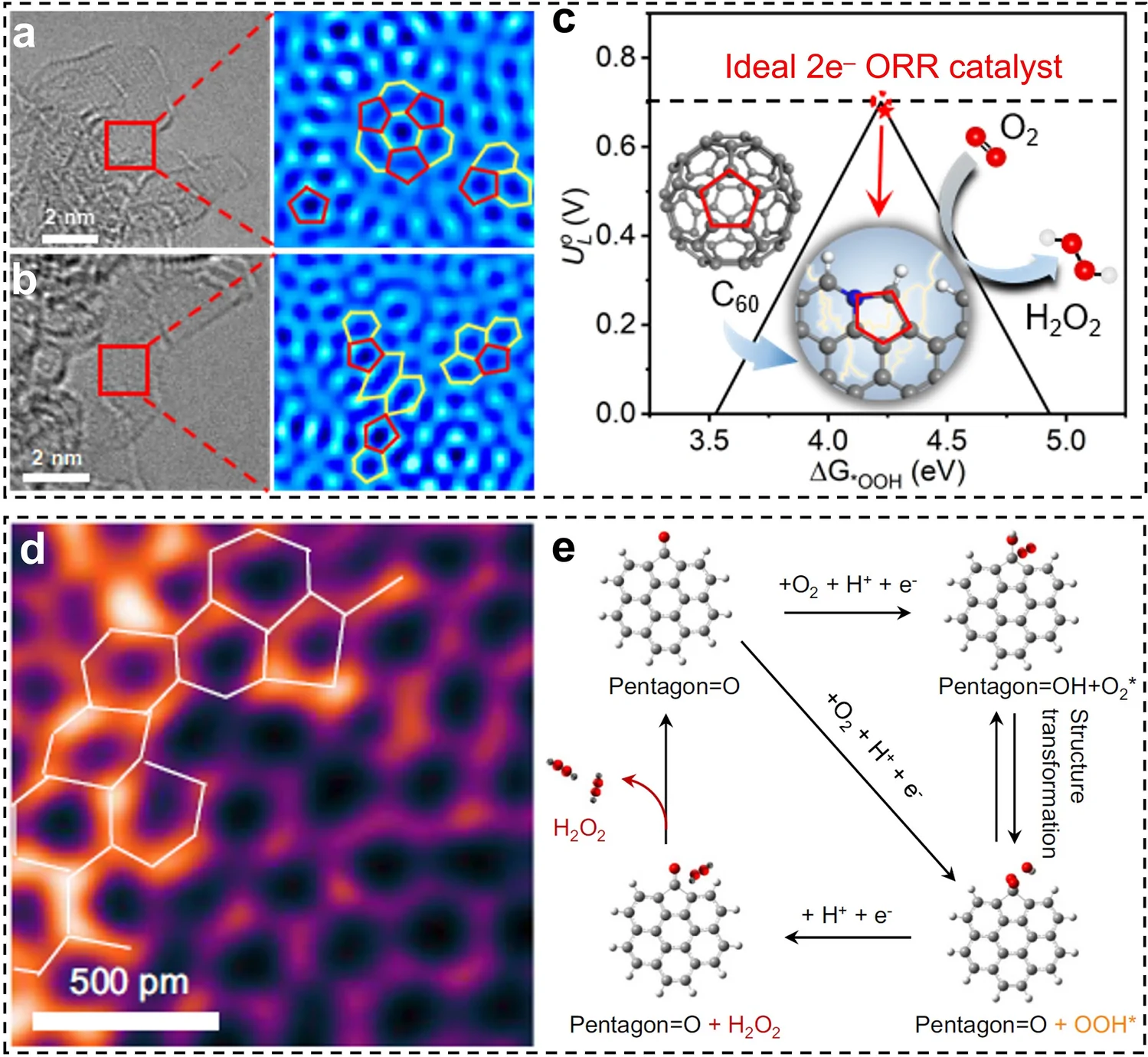
Engineering of heteroatom and defect coengineered carbon materials for 2e^–^ ORR. **a** and **b** AC-STEM and the corresponding fitting images of PD/N–C [230]. Copyright 2023, American Chemical Society. **c** Volcano of limiting potential plotted as a function of ΔG_*OOH_ [230]. Copyright 2023, American Chemical Society. **d** iDPC-STEM image and **e** Schematic diagram of possible electrocatalytic mechanism of O-DG-30. The gray, red, and white balls represent C, O, and H atoms, respectively. Reproduced with permission [231]. Copyright 2023 Springer Nature

Lu et al. designed and synthesized a pentagonal defect-rich nitrogen-doped metal-free nanocarbon (PD/N–C) by creatively using fullerenes with intrinsic five-membered rings as the precursor and the following ammonia treatment [230]. They observed atomic-level pentagonal defects by applying AC-STEM and corresponding Fourier-transform fitting results (Fig. 18a, b). The authors revealed that the intensity of the π* peak in the C K-edge NEXAFS of PD/N–C is significantly lower than that of nitrogen-doped graphene nanosheets. This indicates that the unpaired electrons generated by the unsaturated-coordinated carbon atoms of topological defects induce the weakening of conjugated carbon–carbon bonds. As a result, the PD/N–C achieve a triple-high ORR activity (onset potential of 0.6 V with a current density of 3.0 mA cm^–2^ @ 0.1 V), 2e^–^ selectivity (up to 98%), and stability (over 10 days) in 0.1 M HClO_4_, representing the best performance among all the metal-free carbon electrocatalysts for 2e^–^ ORR in acids. They revealed that neither pentagonal defects nor N dopants alone but together regulate the geometric and electronic structures, resulting in the favorable *OOH adsorption energy that boosts the acidic O_2_ reduction to H_2_O_2_ production (Fig. 18c). Yao et al. investigated the dynamic active site of O-doped defective graphene via the controllable manipulation of the defect density and the type of O groups on carbons [231]. They revealed through DFT calculations that the formation energies of the O groups at the edge sites are generally lower than those of the in-plane sites. Inspired by this, they fabricated a series of defective graphene featuring different defect densities through Ar plasma treatment and subsequent H_2_O_2_ hydrothermal treatment for introducing the O groups. They carried out an integrated differential phase contrast scanning transmission electron microscopy (iDPC-STEM) to present the atomic-level structure characterization of the as-prepared O-DG-30 sample. As shown in Fig. 18d, the clear oxygen species (bright white color) and some pentagon defects on carbon edges were directly identified. Furthermore, their ex situ X-ray photoelectron spectroscopy results disclosed the redistribution of O groups during electrocatalytic activation and identified the carbonyl groups located at the pentagon defects as the main active species in the catalysts (Fig. 18e).

### Chapter Summary

**Fig. 19:**
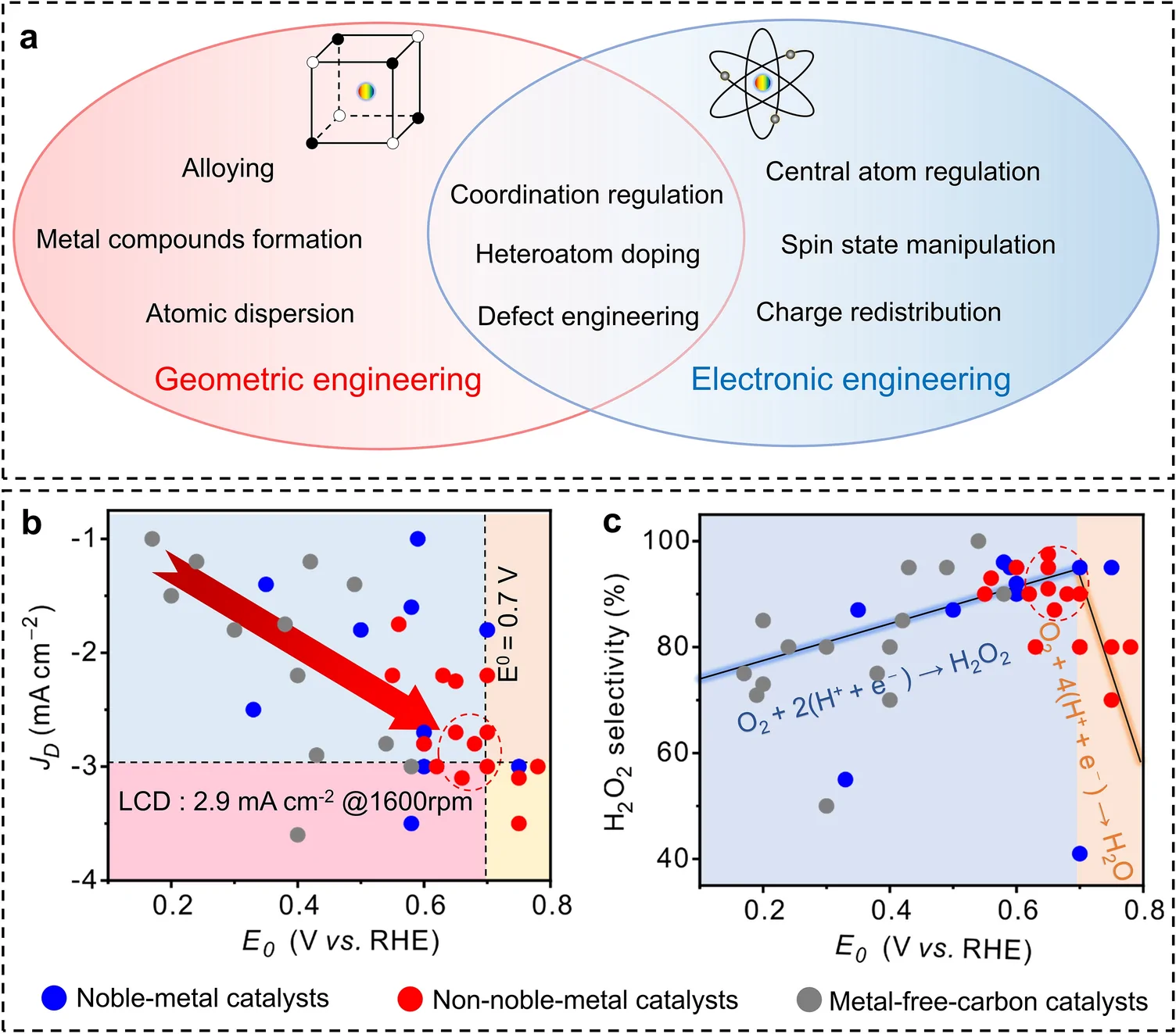
Rules of 2e^–^ ORR catalysts. **a** Schematic illustration of the relationship between geometric engineering and electronic engineering for electrocatalysts toward hydrogen peroxide electrosynthesis.** b** Relationships between *J*_*D*_ and *E*_*0*_ as well as **c** 2e^–^ selectivity and *E*_*0*_ of various advanced catalysts

This paper provides an overview of the cases where the geometric and electronic structure of various materials is regulated to enhance their 2e^–^ ORR capabilities. As summarized in Fig. 19a, geometric engineering mainly involves the regulation of the spatial arrangement and structural configuration of active sites, such as alloying, metal compounds formation, and atomic dispersion, to promote the “end-on” adsorption of O_2_. In contrast, electronic engineering mainly focuses on tuning the intrinsic electronic properties of active centers, including central atom regulation, spin-state manipulation, and charge redistribution, thereby modulating the adsorption strength of intermediates. Importantly, strategies such as coordination regulation, defect engineering, and heteroatom doping often simultaneously alter both the local atomic configuration and the electronic structure of active sites. This overlap indicates that geometric engineering and electronic engineering are better understood as complementary and coupled regulation dimensions in catalyst design. These two strategies work synergistically to accelerate the reaction kinetics without breaking the O–O bond, thereby balancing 2e^–^ ORR activity and selectivity for efficient H_2_O_2_ production.

Herein, we would like to summarize the electrochemical performance of these advanced catalysts to reveal the nature of ideal materials for 2e^–^ ORR. Because H_2_O_2_ tends to self-decompose under alkaline conditions that are not conducive to storage, transport, and application, we focus on the performance of the catalysts in an acidic medium. As shown in Fig. 19b, the disk current density at 0.1 V (*J*_*D*_ @ 0.1 V) of these catalysts increases with the increase of onset potential (*E*_*0*_), indicating that the high oxygen reduction current density of the catalyst is usually accompanied by enhanced ORR activity. However, the theoretical limiting current density (LCD) for 2e^–^ ORR, calculated by the Levich equation under the condition of 1600 rpm and room temperature, is 2.9 mA cm^–2^. Thus, the catalysts that have a higher current density than this value must have a lower H_2_O_2_ selectivity, as we can see in Fig. 19c. Besides, the H_2_O_2_ selectivity of the catalysts increases first with the increase of the *E*_*0*_ and then decreases when the *E*_*0*_ is greater than 0.7 V. It should be noted that the *J*_*D*_ @ 0.1 V of these catalysts (*E*_*0*_ > 0.7 V) is usually greater than 2.9 mA cm^–2^, indicating the 4e^–^ ORR has occurred. The theoretical standard electrode potential for 2e^–^ ORR is 0.7 V. Therefore, the ideal electrocatalytic properties for H_2_O_2_ production are around *E*_*0*_ of 0.7 V and LCD of 2.9 mA cm^–2^. From Tables S1-S3, we can find that the carbon catalysts have a low ORR activity under an acidic medium. Although some noble-metal catalysts exhibit decent performance, their high cost and scarce resources hinder their commercial application. The non-noble-metal catalysts not only show excellent ORR activity and high H_2_O_2_ selectivity but also have low prices and abundant reserves that are considered the best option for 2e^–^ ORR.

## Conclusions and Outlooks

In conclusion, this review provides a comprehensive perspective on how the geometric and electronic structures of electrocatalysts jointly govern ORR activity and 2e^–^ selectivity. We have discussed that the geometric configuration of active sites primarily determines the adsorption mode of oxygen species, whereas the electronic structure regulates the binding strength and conversion behavior of key intermediates. These two factors are intrinsically coupled and together dictate whether the reaction proceeds through the 2e^–^ or 4e^–^ ORR pathway. In addition, we summarized recent progress in the rational design of noble-metal-based, non-noble-metal-based, and carbon-based catalysts through geometric and electronic engineering. Overall, this review highlights that the targeted modulation of catalyst geometry and electronic structure is a powerful route toward highly selective and efficient 2e^–^ ORR electrocatalysis, and it provides a useful framework for the future development of sustainable H_2_O_2_ electrosynthesis technologies.

Despite the rapid progress in this field, several key challenges remain in establishing a clear and general structure–performance relationship for 2e^–^ ORR catalysts. More importantly, future studies should not only focus on identifying these limitations, but also on developing feasible research strategies that can be implemented at the current stage. In this regard, the following directions deserve particular attention.

1. Catalyst design. The intricate composition and structure of catalytically active sites are one of the most prominent obstacles to exploring the structure–performance relationships of the 2e^–^ ORR catalysts. Conventional materials preparation methods, such as pyrolysis and hydrothermal synthesis, involve vigorous reaction processes. These processes trigger unpredictable and disorderly alterations in the structure of the products. Therefore, developing precise synthesis methods for model catalysts with exact geometric and electronic structures is desirable.
2. Structure characteristics. The challenge of catalyst characterization does not simply arise from the lack of advanced techniques, but from the difficulty of correlating structural information with the dynamic catalytic process. Although high-resolution microscopy and spectroscopy have significantly improved our understanding of catalyst structures, future efforts should focus more on establishing quantitative and reaction-relevant descriptors. At the current stage, a feasible strategy is to integrate ex situ characterization with in situ/operando techniques and electrochemical analysis to track the structural evolution of catalysts under working conditions. More importantly, instead of relying on a single characterization result, combining multiple complementary methods with carefully designed control experiments can provide more convincing evidence for active-site identification. This is especially important for catalysts that undergo reconstruction, local coordination changes, or interfacial adsorption reorganization during ORR.
3. Mechanism investigations. Conventional DFT calculations still face inherent limitations in fully rationalizing catalytic behaviors. Most theoretical models rely heavily on the adsorption energy of *OOH as the sole descriptor, yet they often neglect the dynamic evolution of interfacial structures, solvent effects, electrolyte interactions, and the real stabilization of short-lived intermediates under operating conditions. Consequently, catalysts located at the summit of the 2e^–^ ORR volcano plot based on static DFT computations frequently display considerable 4e^–^ ORR activity in practice, indicating a notable discrepancy between theoretical prediction and experimental performance. Moreover, a large number of different atomic environments may provide a wide range of binding energies for ORR intermediates, far exceeding the capabilities of human-led solutions. We believe that the rise of artificial intelligence may change this situation, which prompts the expansion of automated, high-throughput material synthesis and screening capabilities, as well as the utilization of data-hungry machine learning to accelerate the research and development cycle. In addition, the development and application of virtual simulation provide a powerful tool for studying the effect of pore structure on the mass transfer and microenvironment of the catalyst interface.
4. Performance assessment. The limitations of current testing methods represent a significant obstacle to accurately evaluating electrocatalyst performance. Rotating ring disk electrode (RRDE) and gas diffusion electrode (GDE) techniques are widely used, but each has drawbacks: RRDE provides a fast and accessible method to test activity and selectivity but fails to address key questions, such as the H_2_O_2_ yield rate, while GDE offers a testing environment closer to practical applications but is more complex. Additionally, most catalysts are assessed within a three-electrode system, which ensures precise potential and current control but involves greater complexity, whereas the simpler two-electrode system is prone to solution voltage drops and electrode polarization. To overcome these limitations, employing multi-system measurements is recommended for a more comprehensive evaluation of catalyst properties. Furthermore, balancing catalytic activity, selectivity, and stability remains an essential consideration for advancing electrocatalytic research.

In summary, future advances in 2e^–^ ORR electrocatalysis will rely not only on more precise characterization but also on controllable catalyst synthesis, multi-scale mechanistic analysis, standardized performance evaluation, and device-level optimization. By integrating these efforts, the field is expected to move from phenomenological observations toward predictive catalyst design and practical application.

## Supplementary Information

Below is the link to the electronic supplementary material.

Supplementary file1 (DOCX 123 KB)
