# Supplementary material for: Geometric and Electronic Engineering of Hydrogen Peroxide Production Electrocatalysts
Source: Nanomicro Lett. 2026 May 13;18:368. doi: 10.1007/s40820-026-02189-6 (PMC13172137; doi:10.1007/s40820-026-02189-6)
Supplement: Supplementary file 1 — Supplementary file1 (DOCX 123 KB) [file 40820_2026_2189_MOESM1_ESM.docx]

Supporting Information

Geometric and Electronic Engineering of Hydrogen Peroxide Production Electrocatalysts

Chang Zhang^1,2^, Min Song^3^, Huiyao Qi^3^, Hongshang Hu^3^, Lilong Zhang^1^, Houfeng Zhang^1^, Lipiao Bao^1,2^, Huiying Yang^4^, Jian Zhang^1,*^, and Xing Lu^1,2,*^

^1^ School of Chemistry and Chemical Engineering, Hainan University, Haikou, 570228, PR China

^2^ School of Materials Science and Engineering, Huazhong University of Science and Technology, Wuhan, 430074, PR China

^3^ School of Chemistry and Chemical Engineering, Huazhong University of Science and Technology, Wuhan, 430074, PR China

^4^ College of Design and Engineering, National University of Singapore, 21 Lower Kent Ridge Road, 119077, Singapore

*Corresponding author. E-mail: zhangjian7@hainanu.edu.cn (J. Zhang); lux@hust.edu.cn (X. Lu)

**Table S1.** Electrochemical H_2_O_2_ production performance of the various noble–metal–based electrocatalysts, where the *E_0_* is the onset potential.

| Catalyst | RRDE | | | | | Electrochemical reactor | | | Ref. |
| --- | --- | --- | --- | --- | --- | --- | --- | --- | --- |
|  | Electrolyte | | E_0_ (V vs. RHE) | J_disk_ @ 0.1 V （mA cm^–2^） | Selectivity @ 0.1 V（%） | Electrolyte | H_2_O_2_ yield rate | Faraday efficiency （%） |  |
| Au/C | 0.1 M HClO_4_ | 0.15 | | –1.0 | ~75 |  |  |  | [1] |
| PtHg_4_ | 0.1 M HClO_4_ | 0.58 | | –3.5 | 90 |  |  |  | [2] |
| C(Pt)/C–3h | 0.1 M HClO_4_ | 0.70 | | –1.8 | 41 |  |  |  | [3] |
| Pd–Hg | 0.1 M HClO_4_ | 0.75 | | –3.0 @ 0.2 V | 75 @ 0.2 V |  |  |  | [4] |
| 0.35% Pt/TiN | 0.1 M HClO_4_ | 0.33 | | –2.5 | 62 |  |  |  | [5] |
| Pt/HSC | 0.1 M HClO_4_ | 0.58 | | –1.6 | 94 | 0.1 M HClO_4_ | 97.5 μmol h^–1^ cm^–2^ |  | [6] |
| Pd^δ+^–OCNT | 0.1 M HClO_4_ | 0.60 | | –2.7 | 90 | 0.1 M HClO_4_ | 1701mmol g_cat_^–1^ h^–1^ | 87 | [7] |
| Au_0.92_Pd_0.08_/C | 0.1 M HClO_4_ | 0.59 | | –1.0 | 95 |  |  |  | [8] |
| Au/TiC | 0.1 M HClO_4_ | 0.35 | | –1.4 | 89 |  |  |  | [9] |
| Pd_1.0 μM_/GC | 0.1 M HClO4 | 0.6 | | –2.7 | 95 |  |  |  | [10] |
| PtP_2_ NCs | 0.1 M HClO_4_ | 0.60 | | –3.0 | 92 | Neutral water | 2.26 μmol h^–1^ cm^–2^ (512 mg L^–1^ h^–1^) | 78.8 | [11] |
| Pd/GNR–Cl | 0.1 M HClO_4_ | 0.70 | | –1.8 | 86 |  |  |  | [12] |
| Pd_0.157_–NC | 0.1 M KOH | 0.80 | | –3.2 | 88 | 0.1 M KOH | 21 mmol g_cat_^–1^ h^–1^ |  | [13] |
| Pd–Se–B | 0.1 M KPi | 0.70 | | –3.15 | 70 @ 0.3 V | 0.1 M KPi |  | 70 | [14] |
| Pt_0.21_/CN | 0.1 M KOH | 0.81 | | –3.0 | 95 @ 0.2 V | 0.1 M KOH | 767 mmol g_cat_^–1^ h^–1^ | 98 | [15] |
| PdCu NWs/C | 0.1 M HClO_4_ | 0.6 | | –2.9 | 80 |  |  |  | [16] |
| PtSe_2_/C | 0.1 M HClO_4_ | 0.6 | | –2.5 | 91 |  |  |  | [17] |
| Au@Pd(15:1) | 0.1 M HClO_4_ | 0.69 | | -2.9 | 95 |  |  |  | [18] |
| Pt-N-CNT_Gly | 0.1 M HClO_4_ | 0.48 | | -2.2 | 85 | 0.05 M Na_2_SO_4 +_ 0.05 M H_2_SO_4_ | 14705 mmol g_cat_^–1^ h^–1^ | 93 | [19] |
| PdSe2 | 0.05 M NaPi | 0.6 | | -2.2 | 70 | 0.5 M NaPi | 7933 mmol g_cat_^–1^ h^–1^ | 50 | [20] |

**Table S2.** Electrochemical H_2_O_2_ production performance of the various non–noble–metal–based electrocatalysts, where the *E_0_* is the onset potential.

| Catalyst | RRDE | | | | Electrochemical reactor | | | Ref. |
| --- | --- | --- | --- | --- | --- | --- | --- | --- |
|  | Electrolyte | E_0_ (V vs. RHE) | j_disk_ @ 0.1 V (mA cm^–2^) | Selectivity (%) | Electrolyte | H_2_O_2_ yield rate | Faraday efficiency (%) |  |
| Fe_3_O_4_/Graphene | 1 M KOH | 0.80 | –1. 8 @ 0.3 V | 65 |  |  |  | [21] |
| Mn–Ru oxide | 0.1 M KOH | 0.79 | –2.75 | 99 |  |  |  | [22] |
| Co–N–C | 0.5 M H_2_SO_4_ | 0.78 | –3.0 | 80 | 0.1 M KOH | 4.33 mol g_cat_^–1^ h^–1^ (147 mg h^–1^) | 68 | [23] |
|  | 0.1 M K_2_SO_4_ | 0.56 | –3.7 | 55 |  |  |  |  |
|  | 0.1 M KOH | 0.86 | –3.7 | 62 |  |  |  |  |
| Fe–CNT | 0.1 M KOH | 0.82 | –3.4 @ 0.4 V | 85 @ 0.4 V | 0.1 M PBS | 460.9 mg L^–1^ h^–1^ | 90.8 | [24] |
|  | 0.1 M PBS | 0.55 | –3.3 | 80 |  |  |  |  |
| Ni–SA/G–0 | 0.1 M KOH | 0.74 | –3.0 | 94 |  |  |  | [25] |
| Ni–N_2_O_2_/C | 0.1 M KOH | 0.70 | –2.8 | 90 | 0.1 M KOH | 4020 mg L^–1^ h^–1^ | 91 | [26] |
| Co–POC–O | 0.1 M KOH | 0.85 | –2.3 @ 0.4 V | 80 @ 0.5 V | 0.1 M KOH | 813 mg L^–1^ h^–1^ | 64.1 | [27] |
| CoS_2_ | 0.05 M H_2_SO_4_ | 0.75 | –3.5 | 20 | 0.05 M H_2_SO_4_ | 148 mg L^–1^ h^–1^ | 43 | [28] |
| Mo_1_/OSG–H | 0.1 M KOH | 0.78 | –2.8 @ 0.3 V | 95 @ 0.3 V |  |  |  | [29] |
| Co–NC | 0.1 M HClO_4_ | 0.7 | –3.0 | 78 | 0.1 M HClO_4_ | 275 mmol g_cat_^–1^ h^–1^ | 83 | [30] |
| Co_1_–NG(O) | 0.1 M HClO_4_ | 0.68 | –2.2 | 50 @ 0.2 V | 0.1 M KOH | 418 mmol g_cat_^–1^ h^–1^ (947 mg L^–1^ h^–1^) |  | [31] |
|  | 0.1 M PBS | 0.68 | –2.7 | 65 @ 0.2 V |  |  |  |  |
|  | 0.1 M KOH | 0.81 | –2.8 | 80 |  |  |  |  |
| 1% Co SG | 0.1 M Na_2_SO_4_ | 0.8 | –2.8 @ 0.2V | 70 @ 0.2 V |  |  |  | [32] |
| Ni_3_B | 0.1 M KOH | 0.7 | –1.7 | 88 | 0.1 M KOH | 1.19 mg L^–1^ h^–1^ |  | [33] |
| CoNOC | 0.1 M HClO_4_ | 0.57 | –2.8 | 95 | 0.1 M HClO_4_ | 590 mmol g_cat_^–1^ h^–1^ | 95 | [34] |
| Ni MOF NSs–6 | 0.1 M KOH | 0.78 | –3.2 @ 0.2 V | 95 @ 0.2 V | 0.1 M KOH | 80 mmol g_cat_^–1^ h^–1^ |  | [35] |
| Co–N SAC_Dp_ | 0.1 M HClO_4_ | 0.66 | –3.1 | 90 | 0.1 M HClO_4_ | 26.7 mg cm^–2^ h^–1^  (267 mg L^–1^ h^–1^) | 84 | [36] |
| CoNCF–HNO_3_ | 0.05 M H_2_SO_4_ | 0.65 | –2.25 | 83 |  |  |  | [37] |
| Co–N/HPC | 0.1 M HClO_4_ | 0.63 | –2.2 | 78 | 0.1 M KOH | 1.72 mol g_cat_^–1^ h^–1^  (835 mg L^–1^ h^–1^) | 92.3 | [38] |
|  | 0.1 M KOH | 0.8 | –2.7 @ 0.2 V | 95 @ 0.2 V |  |  |  |  |
| p–Co–N–C | 0.5 M H_2_SO_4_ | 0.65 | –2.7 | 90 | 0.5 M H_2_SO_4_ | 2460.8 mg L^–1^ h^–1^ | 88 | [39] |
| HE–CoN@CNTs | 0.1 M HClO_4_ | 0.65 | –2.7 @ 0.2 V | 90 @ 0.3 V | 0.1 M HClO_4_ | 90 mg L^–1^ h^–1^ | 95 | [40] |
| o–CoSe2 | 0.05 M H_2_SO_4_ | 0.75 | –4.2 | 45 |  | 12.91 μmol h^–1^  (120 mg L^–1^ h^–1^) | 83 | [41] |
| MoTe_2_ | 0.5 M H_2_SO_4_ | 0.56 | –1.75 | 90 |  |  |  | [42] |
| NiS_2_ | 0.05 M H_2_SO_4_ | 0.55 | –2.2 | 83 | 0.05 M H_2_SO_4_ | 109 mg L^–1^ h^–1^ | 67 | [43] |
| PFC–72–Co | 0.1 M HClO_4_ | 0.68 | –2.8 | 88 | 0.5 M H_2_SO_4_ |  | 85 | [44] |
| MesoC–Co | 0.1 M HClO_4_ | 0.7 | –2.7 | 62 | 0.1 M HClO_4_ | 7.2 mmol L^–1^ day^–1^ (10.2 mg L^–1^ h^–1^) | 80 | [45] |
| In SAs/NSBC | 0.1 M KOH | 0.8 | –3.2 @ 0.2 V | 90 @ 0.4 V | 0.1 M KOH | 6.49 mol g_cat_^–1^ h^–1^ (1030 mg L^–1^ h^–1^) | 77.3 | [46] |
|  | 0.1 M PBS | 0.52 | –2.6 | 94 | 0.1 M Na_2_SO_4_ | 6.71 mol g_cat_^–1^ h^–1^ (1066 mg L^–1^ h^–1^) | 80.0 |  |
| P–Co@C–700 | 0.1 M HClO_4_ | 0.6 | –3 | 85 | 0.1 M HClO_4_ | 57 mmol g_cat_^–1^ h^–1^ (203.7 mg L^–1^ h^–1^) | 90 | [47] |
| Ni_2–x_P–V_Ni_ | 0.1 M KOH | 0.78 | –3 @ 0.2 V | 95 @ 0.2 V | 0.5 M H_2_SO_4_ | 28.6 mg L^–1^ h^–1^ | 96.2 | [48] |
|  |  |  |  |  | 0.1 M PBS | 38.8 mg L^–1^ h^–1^ | 91.5 |  |
|  |  |  |  |  | 0.1 M KOH | 51 mg L^–1^ h^–1^ | 99 |  |
| CNT–D–O–Fe | 0.1 M KOH | 0.823 | –3 @ 0.4 V | 87 @ 0.4 V |  |  |  | [49] |
| CoNOC | 0.1 M HClO_4_ | 0.6 | –3.5 | 75 | 0.1 M HClO_4_ | 124.7 mM g_cat_^–1^ h^–1^ (13.3 mg L^–1^ h^–1^) | 92 | [50] |
| L–ZnO | 0.6 M K_2_SO_4_ | 0.38 | –0.7 | 88 | 0.6 M K_2_SO_4_ | 624.15 mg cm^–2^ h^–1^ | 98.5 | [51] |
| CoSDC–2 | 0.1 M KOH | 0.8 | –3 | 98 | 0.1 M KOH | 5.58 mmol g_cat_^–1^ h^–1^ |  | [52] |
| BiOS_SA_/Bi_clu_ | 0.1 M KOH | 0.78 | –3.5 @ 0.2 V | 85 @ 0.4 V | 0.1 M KOH | 11.5 mg cm^–2^ h^–1^ | 90 | [53] |
| P–hcp Ni | 0.1 M Na_2_SO_4_ | 0.42 | –2.6 | 97 | 0.1 M Na_2_SO_4_ | 4255.9 mmol g_cat_^–1^ h^–1^ |  | [54] |
|  | 0.1 M PBS | 0.48 | –3 | 90 | Pure water | 4870.5 mmol g_cat_^–1^ h^–1^ |  |  |
|  | 0.1 M KOH | 0.72 | –2.4 @ 0.2 V | 87 @ 0.2 V | 0.1 M KOH | 7402.3 mmol g_cat_^–1^ h^–1^ | 94.6 |  |
| BiNiO_x–4_ | 0.1 M KOH | 0.67 | –2.25 | 93.2 @ 0.4 V | 0.1 M KOH | 39.35 mmol L^–1^ (1337.9 mg L^–1^ h^–1^) | 35 | [55] |
|  | 0.5 M Na_2_SO_4_ |  |  | 96.5 @ 0.3 V | 0.5 M Na_2_SO_4_ | 111.5 mmol L^–1^  (3791 mg L^–1^ h^–1^) | 60 |  |
|  | 0.5 M H_2_SO_4_ |  |  | 87.8 | 0.5 M H_2_SO_4_ | 63.5 mmol L^–1^  (2159 mg L^–1^ h^–1^) | 35 |  |
| Bi@CNR | 0.1 M KOH | 0.77 | –2.8 @ 0.4 V | 94 @ 0.45 V |  |  |  | [56] |
| CoN_4+4_–ACNT | 0.1 M KOH | 0.86 | –4.6 @ 0.2 V | 26 @ 0.2V | 0.1 M KOH | 2.2 mg cm^–2^ h^–1^ | 98 | [57] |
| Co–N/O–C–800 | 0.1 M KOH | 0.85 | –2.8 @ 0.2 V | 62 @ 0.2 V | 0.1 M KOH | 850 mg L^–1^ h^–1^ |  | [58] |
| CoPc–OCNT | 0.1 M K_2_SO_4_ | 0.64 | –3.5 | 88 | 0.1 M K_2_SO_4_ | 62 mM h^–1^  (2108 mg L^–1^ h^–1^) | 98 | [59] |
|  | 0.1 M KOH | 0.78 | –3.4 @ 0.3 V | 88 @ 0.3 V | 1 M KOH | 11527 mmol g_cat_^–1^ h^–1^ |  |  |
|  | 0.01 M KOH | 0.81 | –3 @ 0.2 V | 98 |  |  | 85 |  |
| Co–O/C | 0.05 M H_2_SO_4_ | 0.65 | –2.8 | 69 | 0.05 M H_2_SO_4_ | 110.2 mmol g_cat_^–1^ h^–1^ | 80 | [60] |
| Sb–NSCF | 0.1 M KOH | 0.76 | –3 @ 0.4 V | 90 @ 0.4 V | 1 M KOH | 7.46 mol g_cat_^–1^ h^–1^ (845 mg L^–1^ h^–1^) | 80 | [61] |
| CoN_4_–Pc | 0.1 M KOH | 0.81 | –3 | 93 | 1 M KOH | 11.2 mol g_cat_^–1^ h^–1^ | 80 | [62] |
| Pb SA/OSC | 0.1 M KOH | 0.80 | –2.7 @0.2 V | 90 | 1 M KOH | 6.9 mM cm^–2^ h^–1^ | 92.7 | [63] |
| Meso-FeNSC | 0.1 M PBS | 0.5 | -2.9 | 87.5 |  |  |  | [64] |
| HS/Sb-N-C | 0.1 M KOH | 0.72 | -2.8 | 95 |  |  |  | [65] |
| CoNCB | 0.1 M PBS | 0.76 | -4 | 95 | 1 M PBS | 4.72 mol g_cat_ h^–1^ cm^–2^ |  | [66] |
| Co_1_-NBC | 0.1 M HClO_4_ | 0.72 | -3 | 94 | 0.5 M H_2_SO_4_ | 207.2 mg cm^–2^ h^–1^ | 86 | [67] |
| Fe-n-CNTs | 0.1 M KOH | 0.7 | -2.5 @ 0.2 V | 90 | 1 M KOH | 336.1 mmol h^–1^ | 94.8 | [68] |
| Co/Mo-MCHS | 0.1 M HClO_4_ | 0.7 | -3.2 | 90 | 0.1 M HClO_4_ | 14 mg h^–1^ | 90.6 | [69] |

**Table S3.** Electrochemical H_2_O_2_ production performance performance of the various carbon electrocatalysts, where the *E_0_* is the onset potential.

| Catalyst | RRDE | | | | Electrochemical reactor | | | Ref. |
| --- | --- | --- | --- | --- | --- | --- | --- | --- |
|  | Electrolyte | E_0_ (V vs. RHE) | j_disk_ @ 0.1 V （mA cm^–2^） | Selectivity @ 0.1 V（%） | Electrolyte | H_2_O_2_ yield rate | Faraday efficiency (%) |  |
| GNWCNT–OH | 0.5 M HClO_4_ | 0.20 | –0.06 | 73 |  |  |  | [70] |
| RF–AQ–XC72 | 0.1 M H_2_SO_4_ | 0.24 | –1.2 | 82 | 0.1 M H_2_SO_4_ | 21 μmol cm^–2^ h^–1^  (18 mg L^–1^ h^–1^) | 70 | [71] |
| meso–BMP–800 | 0.1 M HClO_4_ | 0.54 | –2.8 | 100 | 0.1 M HClO_4_ | 3.6 mg L^–1^ h^–1^ | 65.2 | [72] |
| 1% TBAQ | 0.1 M H_2_SO_4_ | 0.19 | –1.7 @ –0.4 V | 70.9 | 0.1 M H_2_SO_4_ | 354 mg L^–1^ h^–1^ | 26.3 | [73] |
| HPC–H24 | 0.05 M H_2_SO_4_ | 0.58 | –3.0 | 90 @ 0 V | 0.05 M H_2_SO_4_ | 2249.4 mg L^–1^ h^–1^ | 91.2 | [74] |
| G250 | 0.1 M Na_2_SO_4_  (pH =3) | 0.17 | –1.0 | 82 |  |  |  | [75] |
| N,S–MC–1 | 0.5 M H_2_SO_4_ | 0.565 | –2 | 77 |  |  |  | [76] |
| CMK–3 | 0.1 M KOH | 0.83 | –3.8 @ 0.2 V | 78 @ 0.2 V | 0.1 M KOH | 3108 mg L^–1^ h^–1^ | 100 | [77] |
| NCMK3IL50_800T | 0.5 M H_2_SO_4_ | 0.42 | –1.4 | 95 | 0.5 M H_2_SO_4_ | 159.9 mmol g_cat_^–1^ h^–1^ | 73 | [78] |
|  | 0.1 M K_2_SO_4_ | 0.48 | –3.4 | 62 | 0.1 M K_2_SO_4_ | 547.07 mmol g_cat_^–1^ h^–1^ | 44 |  |
|  | 0.1 M KOH | 0.78 | –3.8 | 60 | 0.1 M KOH | 561.7 mmol g_cat_^–1^ h^–1^ | 63 |  |
| PEI50CMK3_800T | 0.5 M H_2_SO_4_ | 0.4 | –1.2 | 95 | 0.5 M H_2_SO_4_ | 34.1 mmol g_cat_^–1^ h^–1^ | 47 | [79] |
|  | 0.1 M K_2_SO_4_ | 0.46 | –2.6 | 76 | 0.1 M K_2_SO_4_ | 570.1 mmol g_cat_^–1^ h^–1^ | 59 |  |
|  | 0.1 M KOH | 0.8 | –3.5 | 64 | 0.1 M KOH | 345.5 mmol g_cat_^–1^ h^–1^ | 58 |  |
| FPC–800 | 0.05 M H_2_SO_4_ | 0.30 | –2.5 @ –0.3 V | 86 | 0.5 M H_2_SO_4_ | 2739 mg L^–1^ h^–1^ | 83.3 | [80] |
| MesoC | 0.1 M KOH | 0.76 | –4.2 @ 0.2 V | 65 @ 0.2 V |  |  |  | [81] |
| O–CNTs | 0.1 M HClO_4_ | 0.28 | –2.2 @ 0.4 V | 52 | 0.1 M KOH | 3950 mg L^–1^ h^–1^  (111.7 mmol g_cat_^–1^ h^–1)^ |  | [82] |
|  | 0.1 M PBS | 0.48 | –2.7 | 84 @ 0.2 V |  |  |  |  |
|  | 0.1 M KOH | 0.78 | –2.7 @ 0.4 V | 88 @ 0.4 V |  |  |  |  |
| mrGO | 0.1 M KOH | 0.78 | –3 @ 0.4 V | 100 |  |  |  | [83] |
| g–N–CNHs | 0.1 M H_2_SO_4_ | 0.4 | –2.8 @ –0.2 V | 80 | 0.1 M H_2_SO_4_ | 54 mmol g_cat_^–1^ h^–1^ cm^–2^ | 98 | [84] |
|  | 0.1 M PBS | 0.53 | –0.8 @ –0.2 V | 95 | 0.1 M PBS | 42 mmol g_cat_^–1^ h^–1^ cm^–2^ | 90 |  |
|  | 0.1 M NaOH | 0.71 | –2.4 | 40 | 0.1 M NaOH | 73 mmol g_cat_^–1^ h^–1^ cm^–2^ | 63 |  |
| oxo–G/NH_3_.H_2_O | 0.1 M KOH | 0.78 | –3.1 | 84 | 0.1 M KOH | 224.8 mmol g_cat_^–1^ h^–1^ | 43.6 | [85] |
| CNP_C=O,1_ | 0.1 M KOH | 0.82 | –3.2 @ 0.5 V | 89 @ 0.5 V | 0.1 M KOH | 0.2 mM h^–1^  (6.9 mg L^–1^ h^–1^) | 95 | [86] |
| NPC–1000 | 0.05 M H_2_SO_4_ | 0.7 | –3.5 | 92 | 0.05 M H_2_SO_4_ | 46.0 mmol L^–1^ h^–1^  (1564 mg L^–1^ h^–1^) | 87.7 | [87] |
| MCHS–9:1 | 0.5 M H_2_SO_4_ | 0.4 | –2.2 | 70 |  |  |  | [88] |
|  | 0.1 M PBS | 0.62 | –2.8 @ 0.3 V | 90 @ 0.3 V |  |  |  |  |
|  | 0.1 M KOH | 0.82 | –3 @ 0.4 V | 54 @ 0.4 V |  |  |  |  |
| NT–3DFG | 0.1 M KOH | 0.8 | –2.8 @ 0.4 V | 94 @ 0.5 V |  |  |  | [89] |
| CB+CTAB | 0.1 M KOH | 0.78 | –3.3 | 92 |  |  |  | [90] |
| NPC–950 | 0.1 M KOH | 0.73 | –3.1 | 85 | 1 M KOH | 8.53 mol g_cat_^–1^ h^–1^ | 99 | [91] |
| OCNS_900_ | 0.1 M KOH | 0.825 | –3.1 @ 0.5 V | 85 @ 0.5 V | 0.1 M KOH | 17.8 mg L^–1^ h^–1^ | 89.6 | [92] |
| O–GOMC | 0.1 M KOH | 0.82 | 2.5 @ 0.4 V | 75 @ 0.2 V | 0.1 M KOH | 63.8 mg L^–1^ h^–1^ | 99 | [93] |
| B–C | 0.1 M KOH | 0.77 | –3 @ 0.3 V | 84 @ 0.4 V | DI water | 7.36 mmol cm^–2^ h^–1^ (1100 mg L^–1^ h^–1^ ) | 90 | [94] |
| BNZ–6 | 0.05 M Na_2_SO_4_  (pH = 2) | 0.38 | –2.8 @ –0.3 V | 75 @ –0.3 V | 0.05 M H_2_SO_4_ (pH = 2) | 76.9 mg L^–1^ h^–1^ |  | [95] |
| CF–2 | 0.05 M H_2_SO_4_ | 0.43 | –3.4 @ –0.3 V | 92 @ –0.3 V | 0.05 M H_2_SO_4_ | 945.8 mg L^–1^ h^–1^ |  | [96] |
| NBO–GQDs | 0.1 M KOH | 0.80 | –2.8 @ 0.2 V | 90 | 0.1 M KOH | 241.06 mg L^–1^ h^–1^ | 81 | [97] |
| PD/N–C | 0.1 M HClO_4_ | 0.60 | –3.0 | 78 | 0.1 M HClO_4_ | 2922.9 mg L^–1^ h^–1^ | 98.6 | [98] |
| O–DG–30 | 0.1 M KOH | 0.80 | –2.9 @ 0.2 V | 85 | 0.1 M KOH | 42 mg cm^–2^ | 98.4 | [99] |
| COPN–3 | 0.1 M KOH | 0.72 | –2 | 93 | 1 M KOH | 17.67 mol g_cat_^–1^ h^–1^ (13650 mg L^–1^ h^–1^ ) | 93.2 | [100] |
| 14.0 wt% thiophene-S doping | 1 M KOH | 0.86 | -3.2 @ 0.2 V | 92 | 6 M KOH | 117.7 mg mg_cat_^–1^ h^–1^ | 92.8 | [101] |
| Pe-AB | 0.1 M KOH | 0.75 | -2.5 | 80 | 1 M KOH | 10.68 mol g_cat_^–1^ h^–1^ | 95 | [102] |
| S-Nv-C_3_N_4_ | 0.1 M KOH | 0.73 | -2.4 | 95 | 0.1 M KOH + 10 mM EDTA | 4.52 mol g_cat_^–1^ h^–1^ | 80 | [103] |
| NO-DC_700_ | 0.5 M NaCl | 0.58 | -3 | 75 | 0.5 M NaCl | 4997 mg L^–1^ h^–1^ | 96.5 | [104] |
| CBNO | 0.1 M Na_2_SO_4_ | 0.67 | -2.3 | 87 | 1 M Na_2_SO_4_ | 13.4 mol g_cat_^–1^ h^–1^ | 93 | [105] |
| o-CNT-8 | 0.1 M KOH | 0.795 | -2.3 @ 0.4 V | 90 | DI H_2_O | 3.18 mol g_cat_^–1^ h^–1^ | 92 | [106] |
| CNTs/GDY-O | 0.1 M PBS | 0.46 | -2.5 | 68 | 0.1 M PBS |  | 91.8 | [107] |
| S-DNC | 0.1 M KOH | 0.78 | / | 90 | 0.1 M KOH | 690 mg L^–1^ h^–1^ | 100 | [108] |
| B-DC | 0.1 M KOH | 0.78 | / | 98 | 0.1 M KOH | 247 mg L^–1^ h^–1^ | 100 | [109] |
| OFC650 | 0.1 M KOH | 0.82 | -3.6 @ 0.2 V | 94.5 | 0.1 M KOH | 0.141 mol g_cat_^–1^ h^–1^ | 90.8 | [110] |
| G-MrBC | 0.1 M KOH | 0.77 | -3 @ 0.3 V | 95 | 1.0 M KOH | 5.54 mmol h^–1^ | 96% | [111] |

## References

[1] J. S. Jirkovský, M. Halasa, D. J. Schiffrin, Kinetics of electrocatalytic reduction of oxygen and hydrogen peroxide on dispersed gold nanoparticles. Phys. Chem. Chem. Phys. **12**, 8042–8053 (2010) https://doi.org/10.1039/C002416C

[2] S. Siahrostami, A. Verdaguer-Casadevall, M. Karamad, D. Deiana, P. Malacrida et al., Enabling direct H_2_O_2_ production through rational electrocatalyst design. Nat. Mater. **12**, 1137–1143 (2013) https://doi.org/10.1038/nmat3795

[3] C. H. Choi, H. C. Kwon, S. Yook, H. Shin, H. Kim et al., Hydrogen peroxide synthesis via enhanced two-electron oxygen reduction pathway on carbon-coated Pt surface. J. Phys. Chem. C **118**, 30063–30070 (2014) https://doi.org/10.1021/jp5113894

[4] A. Verdaguer-Casadevall, D. Deiana, M. Karamad, S. Siahrostami, P. Malacrida et al., Trends in the electrochemical synthesis of H_2_O_2_: Enhancing activity and selectivity by electrocatalytic site engineering. Nano Lett. **14**, 1603–1608 (2014) https://doi.org/10.1021/nl500037x

[5] S. Yang, J. Kim, Y. J. Tak, A. Soon, H. Lee, Single-atom catalyst of platinum supported on titanium nitride for selective electrochemical reactions. Angew. Chem. Int. Ed. **55**, 2058–2062 (2016) https://doi.org/10.1002/anie.201509241

[6] C. H. Choi, M. Kim, H. C. Kwon, S. J. Cho, S. Yun et al., Tuning selectivity of electrochemical reactions by atomically dispersed platinum catalyst. Nat. Commun. **7**, 10922 (2016) https://doi.org/10.1038/ncomms10922

[7] Q. Chang, P. Zhang, A. H. B. Mostaghimi, X. Zhao, S. R. Denny et al., Promoting H_2_O_2_ production via 2-electron oxygen reduction by coordinating partially oxidized Pd with defect carbon. Nat. Commun. **11**, 2178 (2020) https://doi.org/10.1038/s41467-020-15843-3

[8] J. S. Jirkovský, I. Panas, E. Ahlberg, M. Halasa, S. Romani et al., Single atom hot-spots at Au–Pd nanoalloys for electrocatalytic H_2_O_2_ production. J. Am. Chem. Soc. **133**, 19432–19441 (2011) https://doi.org/10.1021/ja206477z

[9] S. K. Sahoo, Y. Ye, S. Lee, J. Park, H. Lee et al., Rational design of TiC-supported single-atom electrocatalysts for hydrogen evolution and selective oxygen reduction reactions. ACS Energy Lett. **4**, 126–132 (2019) https://doi.org/10.1021/acsenergylett.8b01942

[10] Y. L. Wang, S. Gurses, N. Felvey, A. Boubnov, S. S. Mao et al., In situ deposition of Pd during oxygen reduction yields highly selective and active electrocatalysts for direct H_2_O_2_ production. ACS Catal. **9**, 8453–8463 (2019) https://doi.org/10.1021/acscatal.9b01758

[11] H. Li, P. Wen, D. S. Itanze, Z. D. Hood, S. Adhikari et al., Scalable neutral H_2_O_2_ electrosynthesis by platinum diphosphide nanocrystals by regulating oxygen reduction reaction pathways. Nat. Commun. **11**, 3928 (2020) https://doi.org/10.1038/s41467-020-17584-9

[12] G. V. Fortunato, E. Pizzutilo, E. S. F. Cardoso, M. R. V. Lanza, I. Katsounaros et al., The oxygen reduction reaction on palladium with low metal loadings: The effects of chlorides on the stability and activity towards hydrogen peroxide. J. Catal. **389**, 400–408 (2020) https://doi.org/10.1016/j.jcat.2020.06.019

[13] N. Wang, X. Zhao, R. Zhang, S. Yu, Z. H. Levell et al., Highly selective oxygen reduction to hydrogen peroxide on a carbon-supported single-atom Pd electrocatalyst. ACS Catal. **12**, 4156–4164 (2022) https://doi.org/10.1021/acscatal.1c05633

[14] J. Lee, S. W. Choi, S. Back, H. Jang, Y. J. Sa, Pd₁₇Se₁₅-Pd₃B nanocoral electrocatalyst for selective oxygen reduction to hydrogen peroxide in near-neutral electrolyte. Appl. Catal. B: Environ. **309**, 121265 (2022) https://doi.org/10.1016/j.apcatb.2022.121265

[15] H. Yang, N. Lu, J. Zhang, R. Wang, S. Tian et al., Ultra-low single-atom Pt on g-C₃N₄ for electrochemical hydrogen peroxide production. Carbon Energy **5**, e337 (2023) https://doi.org/10.1002/cey2.337

[16] J. Du, S. Jiang, R. Zhang, P. Wang, C. Ma et al., Generation of Pd–O for promoting electrochemical H_2_O_2_ production. ACS Catal. **13**, 6887–6892 (2023) https://doi.org/10.1021/acscatal.3c00449

[17] M. Song, M. Chen, C. Zhang, J. Zhang, W. Liu et al., Modulating the oxygen reduction selectivity in Pt or Pd chalcogenides via the ensemble effect and electronic effect. ACS Appl. Mater. Interfaces **15**, 31375–31383 (2023) https://doi.org/10.1021/acsami.3c02793

[18] Z. Deng, A. H. B. Mostaghimi, M. Gong, N. Chen, S. Siahrostami et al., Pd 4d orbital overlapping modulation on Au@Pd nanowires for efficient H_2_O_2_ production. J. Am. Chem. Soc. **146**, 2816–2823 (2024) https://doi.org/10.1021/jacs.3c13259

[19] B. Ni, P. Shen, G. Zhang, J. Zhao, H. Ding et al., Second-shell N dopants regulate acidic O_2_ reduction pathways on isolated Pt sites. J. Am. Chem. Soc. **146**, 11181–11192 (2024) https://doi.org/10.1021/jacs.3c14186

[20] R. D. Ross, K. Lee, G. J. Quintana Cintrón, K. Xu, H. Sheng et al., Stable pentagonal layered palladium diselenide enables rapid electrosynthesis of hydrogen peroxide. J. Am. Chem. Soc. **146**, 15718–15729 (2024) https://doi.org/10.1021/jacs.4c00875

[21] W. R. P. Barros, Q. Wei, G. Zhang, S. Sun, M. R. V. Lanza et al., Oxygen reduction to hydrogen peroxide on Fe₃O₄ nanoparticles supported on Printex carbon and graphene. Electrochim. Acta **162**, 263–270 (2015) https://doi.org/10.1016/j.electacta.2015.02.175

[22] M. B. Zakaria, C. Li, M. Pramanik, Y. Tsujimoto, M. Hu et al., Nanoporous Mn-based electrocatalysts through thermal conversion of cyano-bridged coordination polymers toward ultra-high efficiency hydrogen peroxide production. J. Mater. Chem. A **4**, 9266–9274 (2016) https://doi.org/10.1039/C6TA01470D

[23] Y. Sun, L. Silvioli, N. R. Sahraie, W. Ju, J. Li et al., Activity–selectivity trends in the electrochemical production of hydrogen peroxide over single-site Metal–Nitrogen–Carbon catalysts. J. Am. Chem. Soc. **141**, 12372–12381 (2019) https://doi.org/10.1021/jacs.9b05576

[24] K. Jiang, S. Back, A. J. Akey, C. Xia, Y. Hu et al., Highly selective oxygen reduction to hydrogen peroxide on transition metal single atom coordination. Nat. Commun. **10**, 3997 (2019) https://doi.org/10.1038/s41467-019-11992-2

[25] X. Song, N. Li, H. Zhang, L. Wang, Y. Yan et al., Graphene-supported single nickel atom catalyst for highly selective and efficient hydrogen peroxide production. ACS Appl. Mater. Interfaces **12**, 17519–17527 (2020) https://doi.org/10.1021/acsami.0c01278

[26] Y. Wang, R. Shi, L. Shang, G. I. N. Waterhouse, J. Zhao et al., High-efficiency oxygen reduction to hydrogen peroxide catalyzed by nickel single-atom catalysts with tetradentate N_2_O_2_ coordination in a three-phase flow cell. Angew. Chem. Int. Ed. **59**, 13057–13062 (2020) https://doi.org/10.1002/anie.202004841

[27] B.-Q. Li, C.-X. Zhao, J.-N. Liu, Q. Zhang, Electrosynthesis of hydrogen peroxide synergistically catalyzed by atomic Co–Nₓ–C sites and oxygen functional groups in noble-metal-free electrocatalysts. Adv. Mater. **31**, 1808173 (2019) https://doi.org/10.1002/adma.201808173

[28] H. Sheng, E. D. Hermes, X. Yang, D. Ying, A. N. Janes et al., Electrocatalytic production of H_2_O_2_ by selective oxygen reduction using earth-abundant cobalt pyrite (CoS₂). ACS Catal. **9**, 8433–8442 (2019) https://doi.org/10.1021/acscatal.9b02546

[29] C. Tang, Y. Jiao, B. Shi, J.-N. Liu, Z. Xie et al., Coordination tunes selectivity: Two-electron oxygen reduction on high-loading molybdenum single-atom catalysts. Angew. Chem. Int. Ed. **59**, 9171–9176 (2020) https://doi.org/10.1002/anie.202003842

[30] J. Gao, H. b. Yang, X. Huang, S.-F. Hung, W. Cai et al., Enabling direct H_2_O_2_ production in acidic media through rational design of transition metal single atom catalyst. Chem. **6**, 658–674 (2020) https://doi.org/10.1016/j.chempr.2019.12.008

[31] E. Jung, H. Shin, B.-H. Lee, V. Efremov, S. Lee et al., Atomic-level tuning of Co–N–C catalyst for high-performance electrochemical H_2_O_2_ production. Nat. Mater. **19**, 436–442 (2020) https://doi.org/10.1038/s41563-019-0571-5

[32] N. Li, X. Song, L. Wang, X. Geng, H. Wang et al., Single-atom cobalt catalysts for electrocatalytic hydrodechlorination and oxygen reduction reaction for the degradation of chlorinated organic compounds. ACS Appl. Mater. Interfaces **12**, 24019–24029 (2020) https://doi.org/10.1021/acsami.0c05159

[33] F. Ma, S. Wang, X. Liang, C. Wang, F. Tong et al., Ni₃B as a highly efficient and selective catalyst for the electrosynthesis of hydrogen peroxide. Appl. Catal. B: Environ. **279**, 119371 (2020) https://doi.org/10.1016/j.apcatb.2020.119371

[34] C. Tang, L. Chen, H. Li, L. Li, Y. Jiao et al., Tailoring acidic oxygen reduction selectivity on single-atom catalysts via modification of first and second coordination spheres. J. Am. Chem. Soc. **143**, 7819–7827 (2021) https://doi.org/10.1021/jacs.1c03135

[35] M. Wang, X. Dong, Z. Meng, Z. Hu, Y.-G. Lin et al., An efficient interfacial synthesis of two-dimensional metal–organic framework nanosheets for electrochemical hydrogen peroxide production. Angew. Chem. Int. Ed. **60**, 11190–11195 (2021) https://doi.org/10.1002/anie.202100897

[36] S. Chen, T. Luo, X. Li, K. Chen, J. Fu et al., Identification of the highly active Co–N₄ coordination motif for selective oxygen reduction to hydrogen peroxide. J. Am. Chem. Soc. **144**, 14505–14516 (2022) https://doi.org/10.1021/jacs.2c01194

[37] W. Liu, J. Feng, R. Yin, Y. Ni, D. Zheng et al., Tailoring oxygenated groups of monolithic cobalt-nitrogen-carbon frameworks for highly efficient hydrogen peroxide production in acidic media. Chem. Eng. J. **430**, 132990 (2022) https://doi.org/10.1016/j.cej.2021.132990

[38] Y. Tian, M. Li, Z. Wu, Q. Sun, D. Yuan et al., Edge-hosted atomic Co−N₄ sites on hierarchical porous carbon for highly selective two-electron oxygen reduction reaction. Angew. Chem. Int. Ed. **61**, e202213296 (2022) https://doi.org/10.1002/anie.202213296

[39] J. Zhang, W. Liu, F. He, M. Song, X. Huang et al., Highly dispersed Co atoms anchored in porous nitrogen-doped carbon for acidic H_2_O_2_ electrosynthesis. Chem. Eng. J. **438**, 135619 (2022) https://doi.org/10.1016/j.cej.2022.135619

[40] Q. Zhang, X. Tan, N. M. Bedford, Z. Han, L. Thomsen et al., Direct insights into the role of epoxy groups on cobalt sites for acidic H_2_O_2_ production. Nat. Commun. **11**, 4181 (2020) https://doi.org/10.1038/s41467-020-17782-5

[41] H. Sheng, A. N. Janes, R. D. Ross, D. Kaiman, J. Huang et al., Stable and selective electrosynthesis of hydrogen peroxide and the electro-Fenton process on CoSe₂ polymorph catalysts. Energy Environ. Sci. **13**, 4189–4203 (2020) https://doi.org/10.1039/D0EE01925A

[42] X. Zhao, Y. Wang, Y. Da, X. Wang, T. Wang et al., Selective electrochemical production of hydrogen peroxide at zigzag edges of exfoliated molybdenum telluride nanoflakes. Natl. Sci. Rev. **7**, 1360–1366 (2020) https://doi.org/10.1093/nsr/nwaa084

[43] J. Liang, Y. Wang, Q. Liu, Y. Luo, T. Li et al., Electrocatalytic hydrogen peroxide production in acidic media enabled by NiS₂ nanosheets. J. Mater. Chem. A **9**, 6117–6122 (2021) https://doi.org/10.1039/D0TA12008A

[44] X. Zhao, Q. Yin, X. Mao, C. Cheng, L. Zhang et al., Theory-guided design of hydrogen-bonded cobaltoporphyrin frameworks for highly selective electrochemical H_2_O_2_ production in acid. Nat. Commun. **13**, 2721 (2022) https://doi.org/10.1038/s41467-022-30523-0

[45] L. Jing, Q. Tian, P. Su, H. Li, Y. Zheng et al., Mesoporous Co–O–C nanosheets for electrochemical production of hydrogen peroxide in acidic medium. J. Mater. Chem. A **10**, 4068–4075 (2022) https://doi.org/10.1039/D1TA10416K

[46] E. Zhang, L. Tao, J. An, J. Zhang, L. Meng et al., Engineering the local atomic environments of indium single-atom catalysts for efficient electrochemical production of hydrogen peroxide. Angew. Chem. Int. Ed. **61**, e202117347 (2022) https://doi.org/10.1002/anie.202117347

[47] Y. Wang, Y. Zhou, Y. Feng, X.-Y. Yu, Synergistic electronic and pore structure modulation in open carbon nanocages enabling efficient electrocatalytic production of H_2_O_2_ in acidic medium. Adv. Funct. Mater. **32**, 2110734 (2022) https://doi.org/10.1002/adfm.202110734

[48] Z. Zhou, Y. Kong, H. Tan, Q. Huang, C. Wang et al., Cation-vacancy-enriched nickel phosphide for efficient electrosynthesis of hydrogen peroxides. Adv. Mater. **34**, 2106541 (2022) https://doi.org/10.1002/adma.202106541

[49] T. Gao, L. Qiu, M. Xie, Z. Jin, P. Li et al., Defect-stabilized and oxygen-coordinated iron single-atom sites facilitate hydrogen peroxide electrosynthesis. Mater. Horiz. **10**, 4270–4277 (2023) https://doi.org/10.1039/D3MH00882G

[50] J. Hu, W. Shang, C. Xin, J. Guo, X. Cheng et al., Uncovering dynamic edge-sites in atomic Co−N−C electrocatalyst for selective hydrogen peroxide production. Angew. Chem. Int. Ed. **62**, e202304754 (2023) https://doi.org/10.1002/anie.202304754

[51] S. Ding, B. Xia, M. Li, F. Lou, C. Cheng et al., An abnormal size effect enables ampere-level O_2_ electroreduction to hydrogen peroxide in neutral electrolytes. Energy Environ. Sci. **16**, 3363–3372 (2023) https://doi.org/10.1039/D3EE00509G

[52] D. Qi, J. Xu, Y. Zhou, H. Zhang, J. Shi et al., Cyclodextrin-supported Co(OH)_2_ clusters as electrocatalysts for efficient and selective H_2_O_2_ synthesis. Angew. Chem. Int. Ed. **62**, e202307355 (2023) https://doi.org/10.1002/anie.202307355

[53] P. Zhu, W. Feng, D. Zhao, P. Song, M. Li et al., P-block bismuth nanoclusters sites activated by atomically dispersed bismuth for tandem boosting electrocatalytic hydrogen peroxide production. Angew. Chem. Int. Ed. **62**, e202304488 (2023) https://doi.org/10.1002/anie.202304488

[54] S. Geng, Y. Ji, S. Yang, J. Su, Z. Hu et al., Phosphorus optimized metastable hexagonal-close-packed phase nickel for efficient hydrogen peroxide production in neutral media. Adv. Funct. Mater. **33**, 2300636 (2023) https://doi.org/10.1002/adfm.202300636

[55] Y. Shao, Y. Fei, G. Feng, S. Zhang, X. Peng et al., Electrochemical synthesis of hydrogen peroxide on BiNiOₓ and in situ disinfection. J. Mater. Chem. A **11**, 17661–17670 (2023) https://doi.org/10.1039/D3TA02346J

[56] Z. Zhuang, A. Huang, X. Tan, K. Sun, C. Chen et al., P-block-metal bismuth-based electrocatalysts featuring tunable selectivity for high-performance oxygen reduction reaction. Joule **7**, 1003–1015 (2023) https://doi.org/10.1016/j.joule.2023.04.005

[57] R. Lin, L. Kang, K. Lisowska, W. He, S. Zhao et al., Approaching theoretical performances of electrocatalytic hydrogen peroxide generation by cobalt-nitrogen moieties. Angew. Chem. Int. Ed. **62**, e202301433 (2023) https://doi.org/10.1002/anie.202301433

[58] Z. Liu, D. Wang, M. Zhang, H. Ma, G. Wang, Highly active and selective H_2_O_2_ electrosynthesis in O-rich ZIF-67 derived Co-N/O-C cathode for ofloxacin oxidation. Appl. Catal. B: Environ. **324**, 122252 (2023) https://doi.org/10.1016/j.apcatb.2022.122252

[59] P. Cao, X. Quan, X. Nie, K. Zhao, Y. Liu et al., Metal single-site catalyst design for electrocatalytic production of hydrogen peroxide at industrial-relevant currents. Nat. Commun. **14**, 172 (2023) https://doi.org/10.1038/s41467-023-35839-z

[60] J. Yuan, H. Yin, X. Ge, R. Pan, C. Huang et al., Superior efficiency hydrogen peroxide production in acidic media through epoxy group adjacent to Co-O/C active centers on carbon black. Chem. Eng. J. **465**, 142691 (2023) https://doi.org/10.1016/j.cej.2023.142691

[61] M. Yan, Z. Wei, Z. Gong, B. Johannessen, G. Ye et al., Sb_2_S_3_-templated synthesis of sulfur-doped Sb-N-C with hierarchical architecture and high metal loading for H_2_O_2_ electrosynthesis. Nat. Commun. **14**, 368 (2023) https://doi.org/10.1038/s41467-023-36078-y

[62] J. Liu, Z. Wei, Z. Gong, M. Yan, Y. Hu et al., Single-atom CoN₄ sites with elongated bonding induced by phosphorus doping for efficient H_2_O_2_ electrosynthesis. Appl. Catal. B: Environ. **324**, 122267 (2023) https://doi.org/10.1016/j.apcatb.2022.122267

[63] X. Zhou, Y. Min, C. Zhao, C. Chen, M.-K. Ke et al., Constructing sulfur and oxygen super-coordinated main-group electrocatalysts for selective and cumulative H_2_O_2_ production. Nat. Commun. **15**, 193 (2024) https://doi.org/10.1038/s41467-023-44585-1

[64] W. Zhang, J. Zhang, N. Wang, K. Zhu, C. Yang et al., Two-electron redox chemistry via single-atom catalyst for reversible zinc–air batteries. Nat. Sustain. **7**, 463–473 (2024) https://doi.org/10.1038/s41893-024-01300-2

[65] M. Yan, H. Yang, Z. Gong, J. Zhu, C. Allen et al., Sulfur-tuned main-group Sb−N−C catalysts for selective 2-electron and 4-electron oxygen reduction. Adv. Mater. **36**, 2402963 (2024) https://doi.org/10.1002/adma.202402963

[66] L. Liu, L. Kang, J. Feng, D. G. Hopkinson, C. S. Allen et al., Atomically dispersed asymmetric cobalt electrocatalyst for efficient hydrogen peroxide production in neutral media. Nat. Commun. **15**, 4079 (2024) https://doi.org/10.1038/s41467-024-48209-0

[67] S. Chen, T. Luo, J. Wang, J. Xiang, X. Li et al., Tuning proton affinity on Co−N−C atomic interface to disentangle activity-selectivity trade-off in acidic oxygen reduction to H_2_O_2_. Angew. Chem. Int. Ed. **64**, e202418713 (2025) https://doi.org/10.1002/anie.202418713

[68] H. Chen, C. He, H. Niu, C. Xia, F.-M. Li et al., Surface redox chemistry regulates the reaction microenvironment for efficient hydrogen peroxide generation. J. Am. Chem. Soc. **146**, 15356–15365 (2024) https://doi.org/10.1021/jacs.4c03104

[69] M. Yang, W. Song, C. Chen, X. Yang, Z. Zhuang et al., Atomically dispersed Co/Mo sites anchored on mesoporous carbon hollow spheres for highly selective oxygen reduction to hydrogen peroxide in acidic media. Adv. Mater. **37**, 2416401 (2025) https://doi.org/10.1002/adma.202416401

[70] M. J. Larsen, E. M. Skou, ESR, XPS, and thin-film RRDE characterization of nano structured carbon materials for catalyst support in PEM fuel cells. J. Power Sources **202**, 35–46 (2012) https://doi.org/10.1016/j.jpowsour.2011.11.015

[71] A. Wang, A. Bonakdarpour, D. P. Wilkinson, E. Gyenge, Novel organic redox catalyst for the electroreduction of oxygen to hydrogen peroxide. Electrochim. Acta **66**, 222–229 (2012) https://doi.org/10.1016/j.electacta.2012.01.086

[72] T.-P. Fellinger, F. Hasché, P. Strasser, M. Antonietti, Mesoporous nitrogen-doped carbon for the electrocatalytic synthesis of hydrogen peroxide. J. Am. Chem. Soc. **134**, 4072–4075 (2012) https://doi.org/10.1021/ja300038p

[73] R. B. Valim, R. M. Reis, P. S. Castro, A. S. Lima, R. S. Rocha et al., Electrogeneration of hydrogen peroxide in gas diffusion electrodes modified with tert-butyl-anthraquinone on carbon black support. Carbon **61**, 236–244 (2013) https://doi.org/10.1016/j.carbon.2013.04.100

[74] Y. Liu, X. Quan, X. Fan, H. Wang, S. Chen, High-yield electrosynthesis of hydrogen peroxide from oxygen reduction by hierarchically porous carbon. Angew. Chem. Int. Ed. **54**, 6837–6841 (2015) https://doi.org/10.1002/anie.201502396

[75] C.-Y. Chen, C. Tang, H.-F. Wang, C.-M. Chen, X. Zhang et al., Oxygen reduction reaction on graphene in an electro-Fenton system: In situ generation of H_2_O_2_ for the oxidation of organic compounds. ChemSusChem **9**, 1194–1199 (2016) https://doi.org/10.1002/cssc.201600030

[76] V. Perazzolo, C. Durante, R. Pilot, A. Paduano, J. Zheng et al., Nitrogen and sulfur doped mesoporous carbon as metal-free electrocatalysts for the in situ production of hydrogen peroxide. Carbon **95**, 949–963 (2015) https://doi.org/10.1016/j.carbon.2015.09.002

[77] Z. Chen, S. Chen, S. Siahrostami, P. Chakthranont, C. Hahn et al., Development of a reactor with carbon catalysts for modular-scale, low-cost electrochemical generation of H_2_O_2_. React. Chem. Eng. **2**, 239–245 (2017) https://doi.org/10.1039/C6RE00195E

[78] Y. Sun, I. Sinev, W. Ju, A. Bergmann, S. Dresp et al., Efficient electrochemical hydrogen peroxide production from molecular oxygen on nitrogen-doped mesoporous carbon catalysts. ACS Catal. **8**, 2844–2856 (2018) https://doi.org/10.1021/acscatal.7b03464

[79] Y. Sun, S. Li, Z. P. Jovanov, D. Bernsmeier, H. Wang et al., Structure, activity, and faradaic efficiency of nitrogen-doped porous carbon catalysts for direct electrochemical hydrogen peroxide production. ChemSusChem **11**, 3388–3395 (2018) https://doi.org/10.1002/cssc.201801583

[80] K. Zhao, Y. Su, X. Quan, Y. Liu, S. Chen et al., Enhanced H_2_O_2_ production by selective electrochemical reduction of O_2_ on fluorine-doped hierarchically porous carbon. J. Catal. **357**, 118–126 (2018) https://doi.org/10.1016/j.jcat.2017.11.008

[81] S. Chen, Z. Chen, S. Siahrostami, T. R. Kim, D. Nordlund et al., Defective carbon-based materials for the electrochemical synthesis of hydrogen peroxide. ACS Sustainable Chem. Eng. **6**, 311–317 (2018) https://doi.org/10.1021/acssuschemeng.7b02517

[82] Z. Lu, G. Chen, S. Siahrostami, Z. Chen, K. Liu et al., High-efficiency oxygen reduction to hydrogen peroxide catalysed by oxidized carbon materials. Nat. Catal. **1**, 156–162 (2018) https://doi.org/10.1038/s41929-017-0017-x

[83] H. W. Kim, M. B. Ross, N. Kornienko, L. Zhang, J. Guo et al., Efficient hydrogen peroxide generation using reduced graphene oxide-based oxygen reduction electrocatalysts. Nat. Catal. **1**, 282–290 (2018) https://doi.org/10.1038/s41929-018-0044-2

[84] D. Iglesias, A. Giuliani, M. Melchionna, S. Marchesan, A. Criado et al., N-doped graphitized carbon nanohorns as a forefront electrocatalyst in highly selective O_2_ reduction to H_2_O_2_. Chem. **4**, 106–123 (2018) https://doi.org/10.1016/j.chempr.2017.10.013

[85] L. Han, Y. Sun, S. Li, C. Cheng, C. E. Halbig et al., In-plane carbon lattice-defect regulating electrochemical oxygen reduction to hydrogen peroxide production over nitrogen-doped graphene. ACS Catal. **9**, 1283–1288 (2019) https://doi.org/10.1021/acscatal.8b03734

[86] G.-F. Han, F. Li, W. Zou, M. Karamad, J.-P. Jeon et al., Building and identifying highly active oxygenated groups in carbon materials for oxygen reduction to H_2_O_2_. Nat. Commun. **11**, 2209 (2020) https://doi.org/10.1038/s41467-020-15782-z

[87] D. Zhang, T. Liu, K. Yin, C. Liu, Y. Wei et al., Selective H_2_O_2_ production on N-doped porous carbon from direct carbonization of metal organic frameworks for electro-Fenton mineralization of antibiotics. Chem. Eng. J. **383**, 123184 (2020) https://doi.org/10.1016/j.cej.2019.123184

[88] Y. Pang, K. Wang, H. Xie, Y. Sun, M.-M. Titirici et al., Mesoporous carbon hollow spheres as efficient electrocatalysts for oxygen reduction to hydrogen peroxide in neutral electrolytes. ACS Catal. **10**, 7434–7442 (2020) https://doi.org/10.1021/acscatal.0c00584

[89] D. San Roman, D. Krishnamurthy, R. Garg, H. Hafiz, M. Lamparski et al., Engineering three-dimensional (3D) out-of-plane graphene edge sites for highly selective two-electron oxygen reduction electrocatalysis. ACS Catal. **10**, 1993–2008 (2020) https://doi.org/10.1021/acscatal.9b03919

[90] K.-H. Wu, D. Wang, X. Lu, X. Zhang, Z. Xie et al., Highly selective hydrogen peroxide electrosynthesis on carbon: in situ interface engineering with surfactants. Chem. **6**, 1443–1458 (2020) https://doi.org/10.1016/j.chempr.2020.04.002

[91] P. Cao, X. Quan, K. Zhao, X. Zhao, S. Chen et al., Durable and selective electrochemical H_2_O_2_ synthesis under a large current enabled by the cathode with highly hydrophobic three-phase architecture. ACS Catal. **11**, 13797–13808 (2021) https://doi.org/10.1021/acscatal.1c03236

[92] S. Chen, T. Luo, K. Chen, Y. Lin, J. Fu et al., Chemical identification of catalytically active sites on oxygen-doped carbon nanosheet to decipher the high activity for electro-synthesis hydrogen peroxide. Angew. Chem. Int. Ed. **60**, 16607–16614 (2021) https://doi.org/10.1002/anie.202104480

[93] J. S. Lim, J. H. Kim, J. Woo, D. S. Baek, K. Ihm et al., Designing highly active nanoporous carbon H_2_O_2_ production electrocatalysts through active site identification. Chem. **7**, 3114–3130 (2021) https://doi.org/10.1016/j.chempr.2021.08.007

[94] Y. Xia, X. Zhao, C. Xia, Z.-Y. Wu, P. Zhu et al., Highly active and selective oxygen reduction to H_2_O_2_ on boron-doped carbon for high production rates. Nat. Commun. **12**, 4225 (2021) https://doi.org/10.1038/s41467-021-24329-9

[95] Y. Zhu, M. Tian, Y. Chen, Y. Yang, X. Liu et al., 3D printed triboelectric nanogenerator self-powered electro-Fenton degradation of orange IV and crystal violet system using N-doped biomass carbon catalyst with tunable catalytic activity. Nano Energy **83**, 105824 (2021) https://doi.org/10.1016/j.nanoen.2021.105824

[96] S. Zeng, S. Wang, H. Zhuang, B. Lu, C. Li et al., Fluorine-doped carbon: A metal-free electrocatalyst for oxygen reduction to peroxide. Electrochim. Acta **420**, 140460 (2022) https://doi.org/10.1016/j.electacta.2022.140460

[97] M. Fan, Z. Wang, K. Sun, A. Wang, Y. Zhao et al., N–B–OH site-activated graphene quantum dots for boosting electrochemical hydrogen peroxide production. Adv. Mater. **35**, 2209086 (2023) https://doi.org/10.1002/adma.202209086

[98] C. Zhang, W. Shen, K. Guo, M. Xiong, J. Zhang et al., A pentagonal defect-rich metal-free carbon electrocatalyst for boosting acidic O_2_ reduction to H_2_O_2_ production. J. Am. Chem. Soc. **145**, 11589–11598 (2023) https://doi.org/10.1021/jacs.3c00689

[99] Q. Wu, H. Zou, X. Mao, J. He, Y. Shi et al., Unveiling the dynamic active site of defective carbon-based electrocatalysts for hydrogen peroxide production. Nat. Commun. **14**, 6275 (2023) https://doi.org/10.1038/s41467-023-41947-7

[100] Z. Bao, Q. Cao, Y. Shao, S. Zhang, X. Peng et al., Carbon and oxygen-doped phosphorus nitride (COPN) for continuous selective and stable H_2_O_2_ production. ACS Catal. **13**, 14492–14502 (2023) https://doi.org/10.1021/acscatal.3c03775

[101] R. Xie, C. Cheng, R. Wang, J. Li, E. Zhao et al., Maximizing thiophene–sulfur functional groups in carbon catalysts for highly selective H_2_O_2_ electrosynthesis. ACS Catal. **14**, 4471–4477 (2024) https://doi.org/10.1021/acscatal.4c00419

[102] D. Zhao, D. Jiao, L. Yi, Y. Yu, J. Zou et al., Tandem oxidation activation of carbon for enhanced electrochemical synthesis of H_2_O_2_: Unveiling the role of quinone groups and their operando derivatives. Small **20**, 2406890 (2024) https://doi.org/10.1002/smll.202406890

[103] S. Xu, Y. Yu, X. Zhang, D. Xue, Y. Wei et al., Enhanced electron delocalization induced by ferromagnetic sulfur doped C₃N₄ triggers selective H_2_O_2_ production. Angew. Chem. Int. Ed. **63**, e202407578 (2024) https://doi.org/10.1002/anie.202407578

[104] X. Wang, W. Shen, C. Zhang, Y. Huang, J. Zhang et al., A chlorine-resistant self-doped nanocarbon catalyst for boosting hydrogen peroxide synthesis in seawater. Angew. Chem. Int. Ed. **64**, e202419049 (2025) https://doi.org/10.1002/anie.202419049

[105] Z. Song, X. Chi, S. Dong, B. Meng, X. Yu et al., Carboxylated hexagonal boron nitride/graphene configuration for electrosynthesis of high-concentration neutral hydrogen peroxide. Angew. Chem. Int. Ed. **63**, e202317267 (2024) https://doi.org/10.1002/anie.202317267

[106] F. She, Z. Guo, F. Liu, Z. Yu, J. Chen et al., Curvature-dependent electrochemical hydrogen peroxide synthesis performance of oxidized carbon nanotubes. ACS Catal. **14**, 10928–10938 (2024) https://doi.org/10.1021/acscatal.4c01637

[107] T. Lu, M. Sun, F. Wang, S. Chen, Y. Li et al., Selective oxidation of sp-bonded carbon in graphdiyne/carbon nanotubes heterostructures to form dominant epoxy groups for two-electron oxygen reduction. ACS Nano **18**, 15035–15045 (2024) https://doi.org/10.1021/acsnano.4c01698

[108] W. Shen, C. Zhang, X. Wang, Y. Huang, Z. Du et al., Sulfur-doped defective nanocarbons derived from fullerenes as electrocatalysts for efficient and selective H_2_O_2_ electroproduction. ACS Materials Lett. **6**, 17–26 (2024) https://doi.org/10.1021/acsmaterialslett.3c01036

[109] W. Shen, C. Zhang, M. Alomar, Z. Du, Z. Yang et al., Fullerene-derived boron-doped defective nanocarbon for highly selective H_2_O_2_ electrosynthesis. Nano Res. **17**, 1217–1224 (2024) https://doi.org/10.1007/s12274-023-5999-x

[110] A. Yu, J. Shi, W. Yang, G. Ma, Q. Huang et al., Direct cathodic polarization preparation of ambient CO₂-derived oxygen-functionalized carbons for electro-production of H_2_O_2_. Appl. Catal. B: Environ. **367**, 125109 (2025) https://doi.org/10.1016/j.apcatb.2025.125109

[111] W. Cui, Z. Zhen, Y. Sun, X. Liu, J. Chen et al., Vacancy-activated B-doping for efficient 2e⁻ oxygen reduction through suppressing H_2_O_2_ decomposition at high overpotential. Angew. Chem. Int. Ed. **64**, e202423056 (2025) https://doi.org/10.1002/anie.202423056
